# Temperature elevation and *Vibrio cyclitrophicus* infection reduce the diversity of haemolymph microbiome of the mussel *Mytilus coruscus*

**DOI:** 10.1038/s41598-019-52752-y

**Published:** 2019-11-08

**Authors:** Yi-Feng Li, Yan-Wen Chen, Jia-Kang Xu, Wen-Yang Ding, An-Qi Shao, You-Ting Zhu, Chong Wang, Xiao Liang, Jin-Long Yang

**Affiliations:** 1 International Research Center for Marine Biosciences, Ministry of Science and Technology, Shanghai Ocean University, Shanghai, China; 2 Key Laboratory of Exploration and Utilization of Aquatic Genetic Resources, Ministry of Education, Shanghai Ocean University, Shanghai, China; 3 National Demonstration Center for Experimental Fisheries Science Education, Shanghai Ocean University, Shanghai, China; 4 Ocean and Fisheries Research Institute of Binzhou, Binzhou, China

**Keywords:** Animal physiology, Microbial ecology

## Abstract

Haemolymph microbiome was considered to be unique to healthy invertebrates and beneficial to the host against external pathogens, including disease resistance and maintenance of homeostasis. Here, we investigated the effects of elevated water temperature on infection of haemolymph microbiome of the hard-shelled mussel (*Mytilus coruscus*). Exposure to *Vibrio*. *cyclitrophicus* resulted in high mortality of mussels on day nine at 27 °C. The haemolymph was collected to determine the microbiota by 16 S rRNA gene sequencing. Exposure to waterborne *V*. *cyclitrophicus* increased the mortality of mussels that was associated with a reduction in the diversity of their microbial community. Principal coordinate analysis (PCoA) revealed that temperature was an essential factor in shaping microbial communities in mussel haemolymph. *Vibrio* exposure promoted the proliferation of opportunistic pathogens (e.g., *Arcobacter* and *Francisella*) at a lower temperature. A high abundance of *Vibrio* present in live and dead mussels, at 27 °C might contribute greatly to mortality, as indicated by linear discriminant analysis effect size (LEfSe). These data suggested that the dynamics of microbial community have unique biomarker species in mussel haemolymph that could be used as health indicators. An elevated temperature may reduce the ability of bacterial elimination function against infection in mussel haemolymph.

## Introduction

Microbial communities that colonize various tissues are fundamentally essential factors for maintaining homeostasis, metabolism and development in the host^1–3^. In vertebrates, the circulatory system is considered “sterile” without the proliferation of microorganisms in healthy animals^4^. Unlike vertebrates, some aquatic invertebrates have an open circulatory system where their blood or haemolymph spread through the entire body^5^. Increasing evidence has revealed the presence of viable microbes in the haemolymph of aquatic invertebrates^3^. The bacteria abundance in the haemolymph of healthy invertebrates can reach to 10^3^ CFU/mL, which were detected by culture-dependent approaches^3^. For example, the haemolymph of horse mussel (*Modiolus modiolus*) and Pacific oyster (*Crassostrea gigas*) harbour the same amounts of bacteria flora with the dominant genera of *Vibrio*, *Pseudomonas*, *Alteromonas* and *Aeromonas*^6^.

Disturbance of microbial communities in haemolymph reflected the host fitness and had growth inhibition or even lethal effects on the host^7^. The biotic and abiotic factors shape the dynamics of indigenous haemolymph microbiome as well as the adjustments and managements of microbial communities functioning^8,9^. Microbiota in haemolymph may be involved in the host immune defence to prevent pathogen infection and colonization by producing antimicrobial compounds^10,11^. Shifts from symbiotic bacteria to pathogen-dominated communities, due to environmental factors and stress, highlight the importance of crosstalk between environment and host-associated microbial communities^8,12^.

Temperature as an abiotic factor has a crucial impact on the haemolymph microbiome in many invertebrates^4–6,8^. Moreover, warm temperature altered microbial communities in coral mucus and favoured proliferation of pathogen-dominated microbes as well as increased the susceptibility of the host to diseases^12,13^. Exceeding the limit of thermal tolerance has specific adverse effects on bivalves^14–17^. In the case of the Pacific oyster (*C*. *gigas*), temperature stress reduced microbial diversity in haemolymph and moribund oysters were dominated by a few strains^8^. The lethal effects caused by heat stress highlights the importance of the host-bacteria interaction in haemolymph, which may indicate the health status of the host^4,8,16,17^. Previously we demonstrated that high temperature modified the gut microbiome and facilitated the opportunistic bacteria proliferation of the hard-shelled mussel (*Mytilus coruscus*)^16^.

Vibrios are ubiquitous in the marine environment, and they are characterized as opportunistic pathogens resulting in disease outbreaks in bivalve species such as *C*. *gigas* and *M*. *galloprovincialis*^18–20^. The beneficial animal-bacterial interactions were observed in the symbionts *Vibrio fischeri*, which inhabit of the light organ crypts in the squid *Euprymna scolopes*^21^. *V*. *coralliilyticus* has been reported as a threat to mass mortality of bivalve associated with vibriosis outbreaks^22^. Vibriosis infection events frequently occurred in larval rearing stage of shellfish production hatcheries in the United States, Canada and Mexico, leading to shortages in seed oysters^23,24^. Summer mortality syndrome (SMS) of bivalve species was influenced by high water temperature in summer, which was associated with the vibriosis outbreaks^25^. The bivalves as marine poikilotherms and their haemolymph microbiome could be used as bioindicators to mirror the host fitness when they suffered environmental stress^8^.

In the current study, we have isolated a *Vibrio* strain from marine biofilms in the natural habitat of *M*. *coruscus*, and this isolate showed lethal consequences in larval settlement and metamorphosis trials. To explain the effects of infection and temperature on mussel survival and the dynamics of bacterial communities in haemolymph, the hard-shelled mussel (*M*. *coruscus*) were challenged with this *Vibrio* strain and exposed to a different temperature. The disturbances in haemolymph microbiome were determined by Illumina Hiseq sequencing of 16S rRNA gene.

## Results

### Haemolymph microbiome analysis

Globally 1947 operational taxonomic units (OTUs) were identified from the haemolymph samples. At a 3% dissimilarity level, Good’s coverage estimator showing 99.5% to 99.7% of the OTUs were identified for all the groups and the rarefaction curve of haemolymph samples tended to approach the saturation plateau (Fig. S1).

### Mussel haemolymph microbiome at phylum level

**Figure 1:**
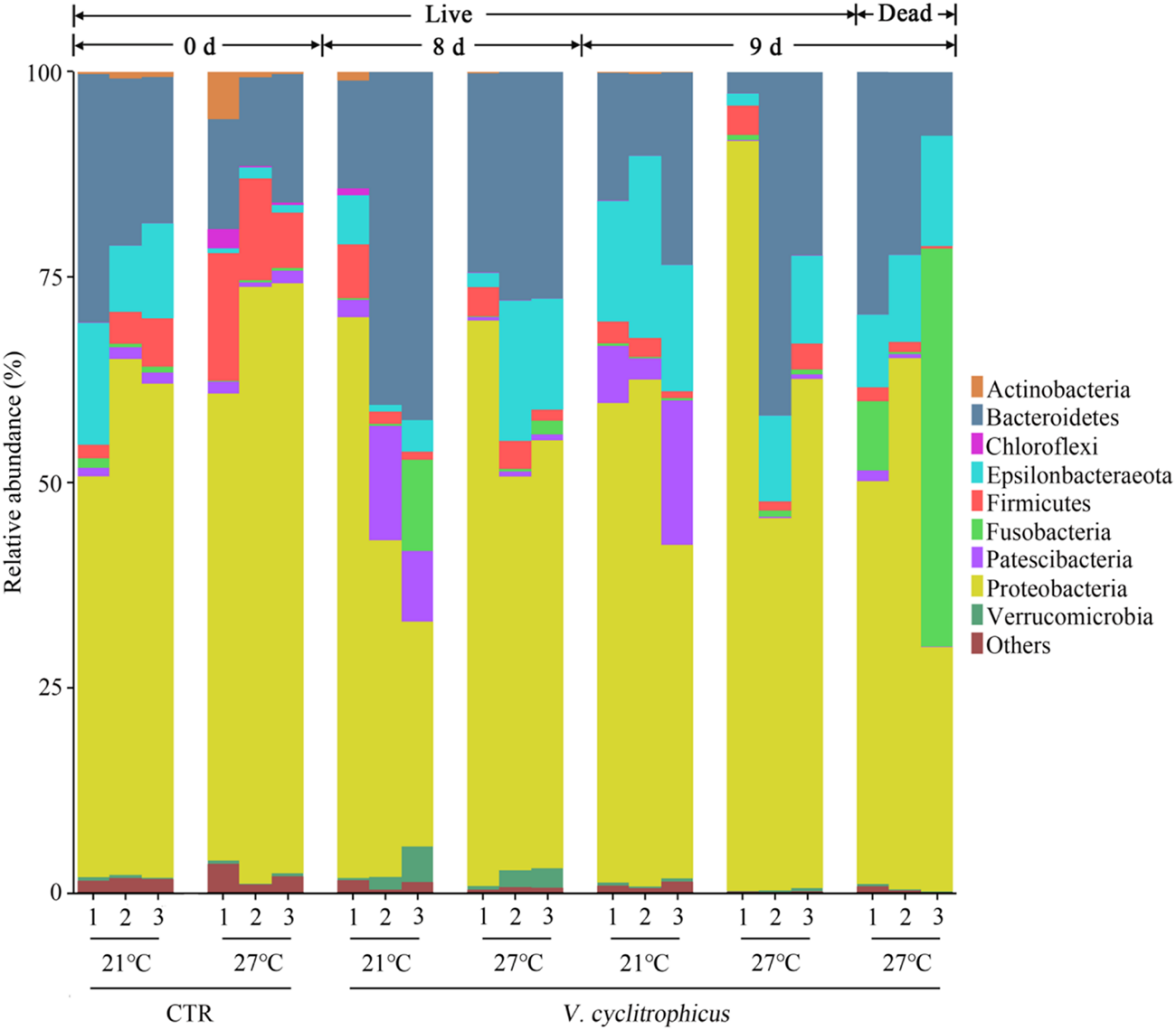
The relative abundance of bacterial communities at the phylum level of haemolymph samples. Three replicates are labelled with the numbers 1, 2 and 3.

A total of 9 different phyla with an abundance of >1% was identified, and the abundance of 27 phyla < 1% was all classified as “others” (Fig. 1). Proteobacteria, Epsilonbacteraeota and Bacteroidetes were the three dominant phyla which accounted for 51.0%–97.5% of the total reads (Fig. 1; Table S1). In Control (CTR) groups, higher temperature (27 °C) significantly increased the relative abundance of Chloroflexi and Firmicutes. A significant reduction was observed in the relative abundance of Bacteroidetes, Epsilonbacteraeota and Fusobacteria on day 0 (*P* < 0.05, Table S1). At 21 °C, the relative abundance of Patescibacteria significantly increased in the treatment groups on day 8 and 9 relative to CTR groups (day 0) (*P* < 0.05, Table S1). In the live mussels, higher temperature (27 °C) caused a significant decrease in the relative abundance of Actinobacteria, Chloroflexi and Firmicutes in treatment groups on day 8 and day 9 relative to CTR groups (day 0), accompanied by a significant increase in the relative abundance of Epsilonbacteraeota (*P* < 0.05, Table S1). At 27 °C, the bacterial phylum Chloroflexi significantly decreased in the dead mussels relative to live mussels on day 9 (*P* < 0.05, Table S1).

### Mussel haemolymph microbiome at genus level

**Figure 2:**
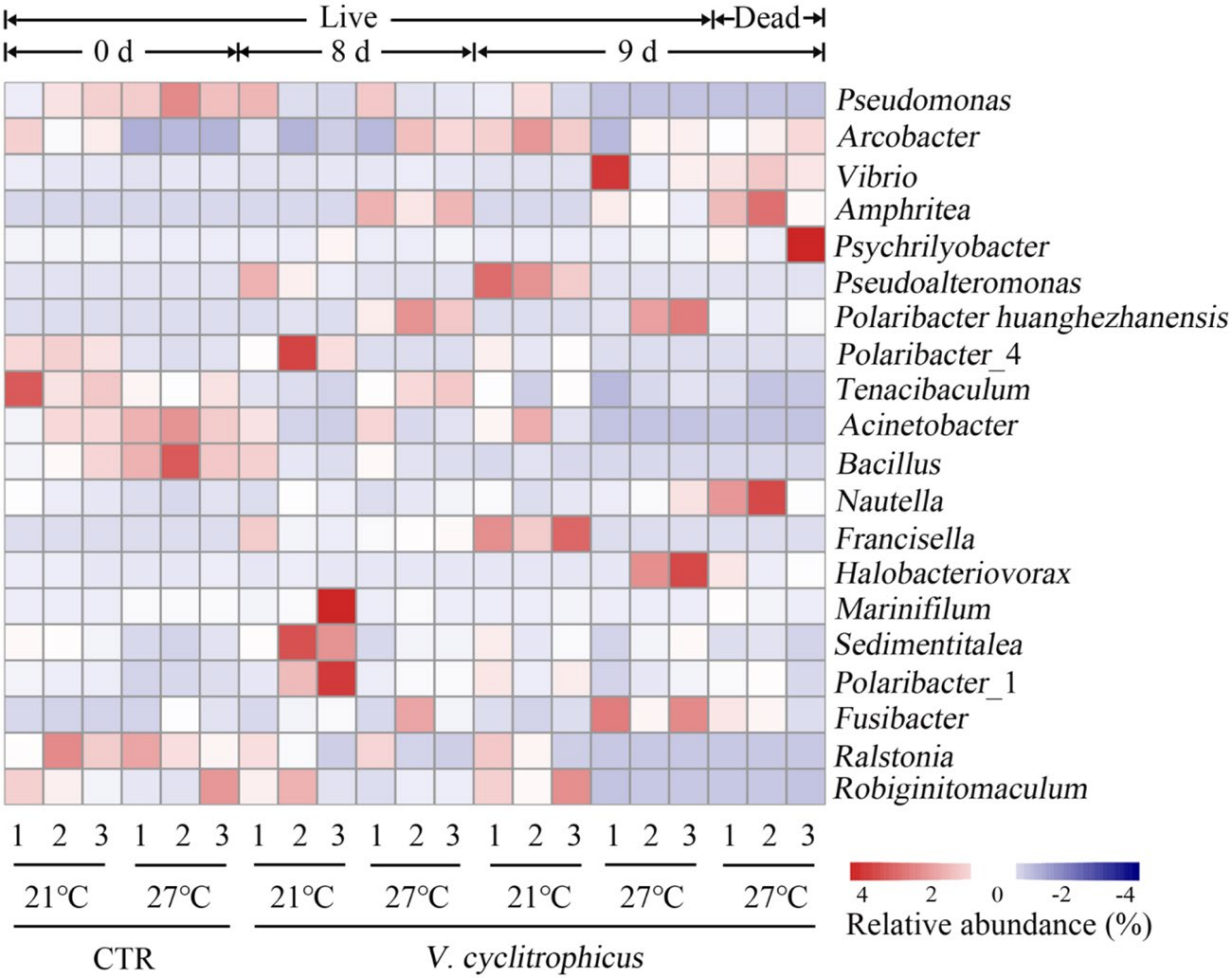
Heatmap is revealing the top 20 bacterial genera (%) of haemolymph samples. Three replicates are labelled with the numbers 1, 2 and 3 (red colours indicate higher abundance; blue colours indicate lower abundance).

The top 20 abundant genera were selected for comparative analysis (Fig. 2). The genus *Pseudomonas* dominated the CTR groups, and its abundance increased significantly in CTR groups at a higher temperature (27 °C) (*P* < 0.05, Table S2). Compared to CTR groups (day 0), *Pseudoalteromonas* and *Francisella* were much more abundant in treatment groups on day 8 and day 9 at 21 °C (*P* < 0.05, Table S2). At elevated temperature (27 °C), the relative abundance of *Arcobacter*, *Amphritea*, *Pseudoalteromonas* and *Polaribacter huanghezhanensis* increased significantly in treatment groups on day 8 and day 9 compared to CTR groups (day 0), while a significant decrease was observed for *Acinetobacter*, *Bacillus* and *Marinifilum* (*P* < 0.05, Table S2). However, there was no significant difference between the live and dead mussels at 27 °C on genus level (*P* > 0.05, Table S2). The treatment groups on day 8 were clustered with both two CTR groups (21 and 27 °C). In contrast, the live and dead mussels from treatment groups on day 9 shared high similarity and formed a cluster.

**Figure 3:**
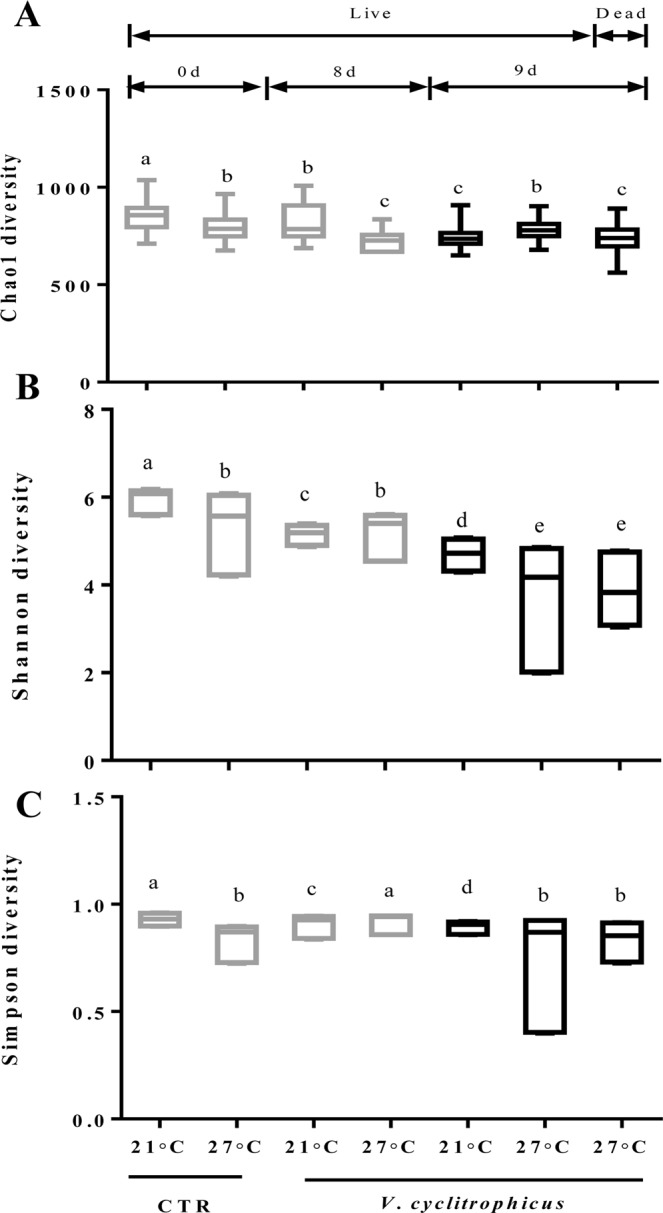
Microbial diversity indices of Chao1 (**A**), Shannon (**B**) and Simpson (**C**). Data are the mean ± SE (n = 3). Different letters represent significant differences (*P* < 0.05).

Alpha diversity metrics of the total species abundance index (Chao1) and species diversity indices (Shannon and Simpson) were presented in Fig. 3. Higher temperature largely reduced the Chao1, Shannon and Simpson indices in CTR groups (*P* < 0.05). At 21 °C, the infection by *V*. *cyclitrophicus* significantly decreased the Chao1, Shannon and Simpson indices in treatment groups relative to CTR groups (*P* < 0.05). At 27 °C, a significant reduction was observed in treatments groups on day 8 (Chao1) and day 9 (Shannon) in comparison to CTR groups, respectively (*P* < 0.05). However, the Simpson index increased significantly in the treatment groups on day 8 relative to CTR groups (*P* < 0.05). Chao1 index showed a significant difference between live and dead mussel at 27 °C (*P* < 0.05), while no difference was observed for Shannon and Simpson (*P* > 0.05).

**Figure 4:**
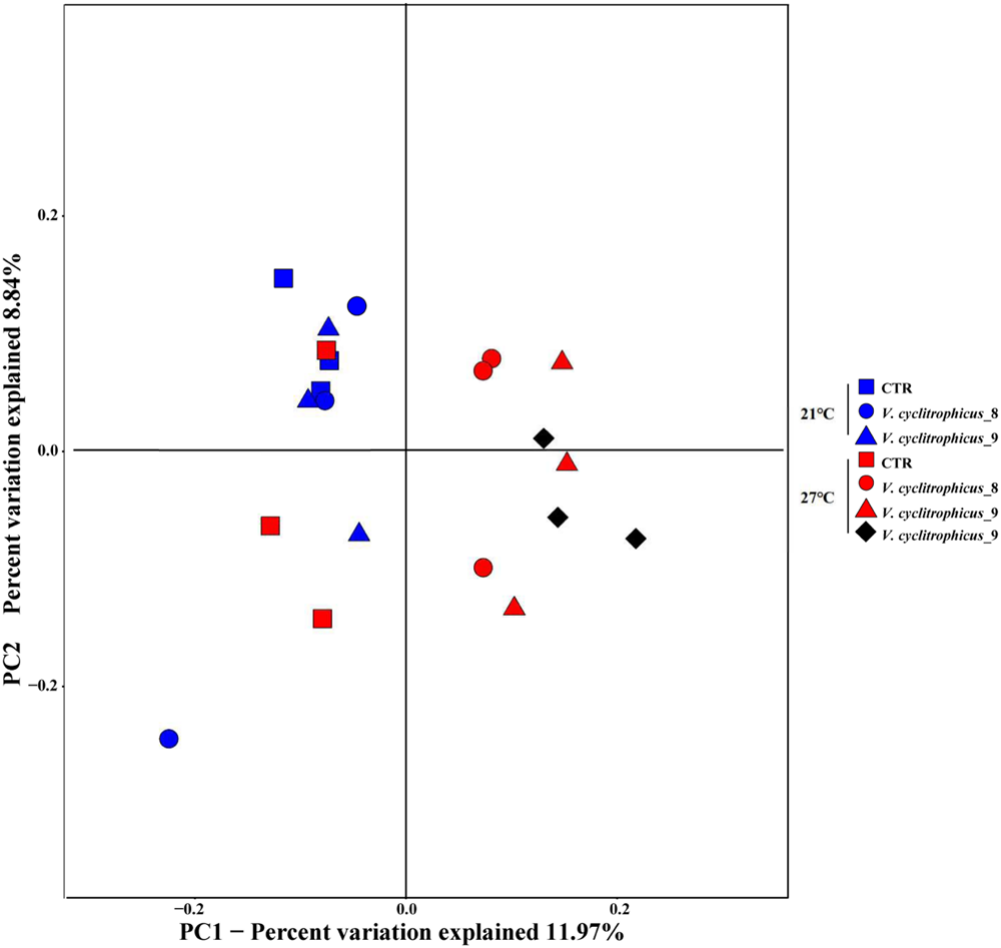
Principal components analysis of haemolymph microbiome. Blue and red means live mussel samples. Squares mean control mussels sampled on day 0; Spots mean mussels exposed to *V*. *cyclitrophicus* and sampled on day 8; Triangles represent mussels exposed to *V*. *cyclitrophicus* and sampled on day 9; Black and rhombus represent dead mussel sampled on day 9.

Principal coordinate analysis (PCoA) revealed that temperature shaped the bacterial community composition by separating the treatment groups at 21 °C from the groups at 27 °C by PC1, which contributed to >11.97% of the variance (Fig. 4). At 27 °C, a separation by PC1 was found between treatment groups and CTR groups. The live and dead mussels in treatment groups on day 8 and day 9 at 27 °C formed a cluster. The relations between samples and environmental variables were investigated by Canonical correspondence analysis (CCA) (Fig. S2). The results indicated that the first and second axes together explained 23.45% of the cumulative variance of the samples-environment factor correlation. The temperature and infection significantly affected the haemolymph microbiome with the Pr value of 0.001 and 0.002, respectively.

### Unique biomarkers detected in the mussel haemolymph

**Figure 5:**
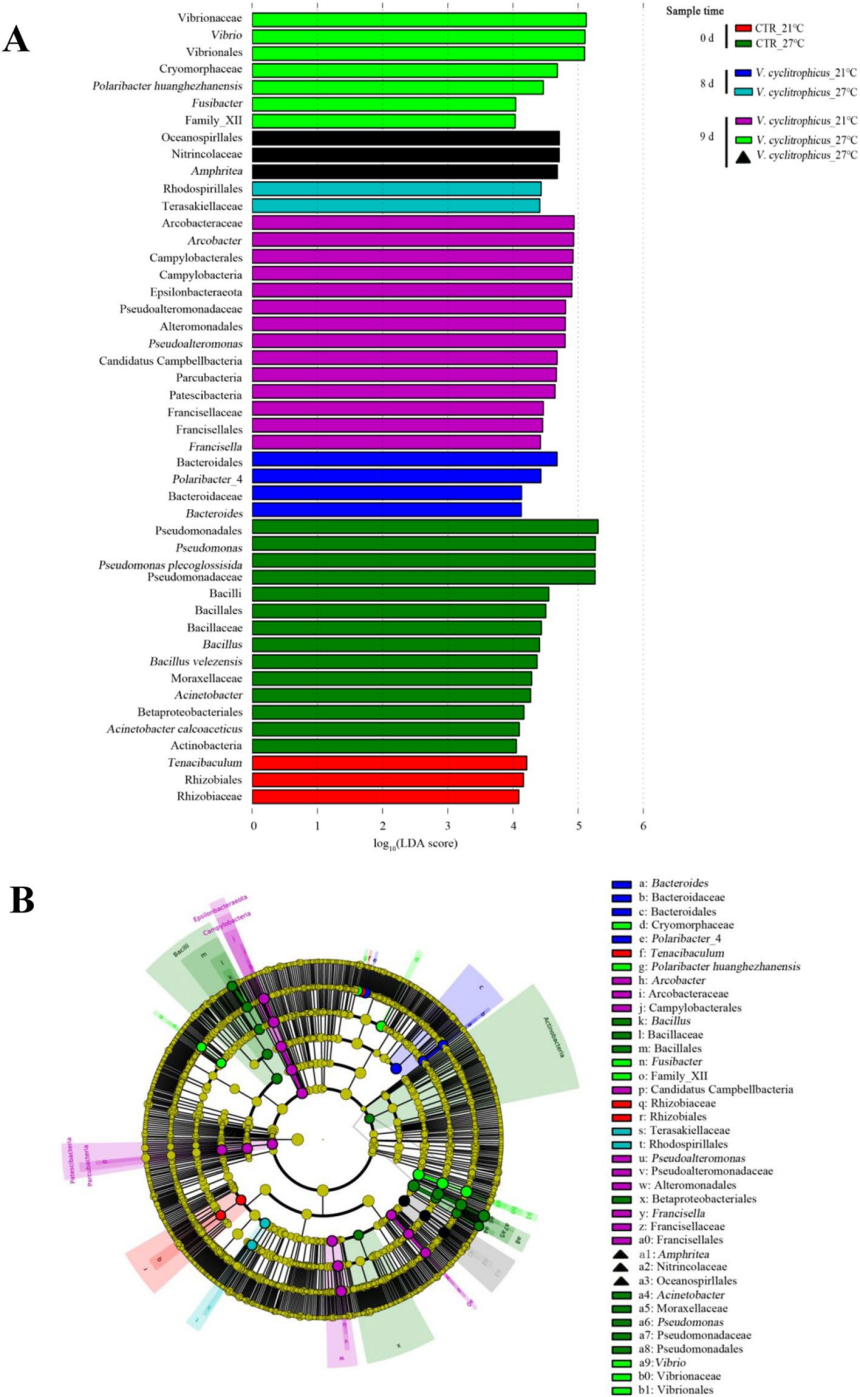
Unique community composition of biomarkers in mussel haemolymph. (**A**) Bar chart showing the log-transformed LDA scores of bacterial taxa identified by LEfSe analysis. A log-transformed LDA score of 2 was used as a threshold for identification of significant taxa, 47 taxa were identified by LEfSe analysis and are shown (Supplementary Table 3). (**B)** Cladogram showing the phylogenetic relationships of 47 bacterial taxa revealed by LEfSe. The squares mean live mussels; Triangle represents dead mussels.

LEfSe analysis revealed that *Pseudomonas* and *Bacillus* were the top genus-level biomarkers that distinguished the CTR groups at 27 °C from all other groups (Fig. 5; Table S3). At 21 °C, exposure to waterborne *V*. *cyclitrophicus* on day 8 and day 9 was distinguished from all other host groups by the genera of *Arcobacter*, *Francisella* and *Polaribacter*_4. At 27 °C, *Vibrio* and *Amphritea* were the top genus-level biomarkers in treatment groups of live and dead mussels, respectively.

## Discussion

Haemolymph microbiome is considered to be essential for healthy animals to maintain homeostasis and disease resistant in invertebrates^4^. The present study showed that the exposure to waterborne *V*. *cyclitrophicus* resulted in high mortality of mussels on day 9 with increasing seawater temperature from 21 to 27 °C. Elevated water temperature reduced microbial diversity of mussel haemolymph. The increased mussel mortality upon infection was associated with a decline of microbial diversity, as indicated by the Shannon index. Principal coordinate analysis (PCoA) revealed that temperature was an important factor in shaping microbial communities in mussel haemolymph between the *V*. *cyclitrophicus* exposed and CTR mussels. Unique biomarker species in mussel haemolymph assessed by LEfSe analysis could be health indicators in changing environments.

The elevated temperature has been proposed to be a critical factor in regulating bacteria virulence^26,27^ and innate immunity in bivalves^28^. Elevated temperature and exposure to waterborne *V*. *cyclitrophicus* resulted in high mortality, but temperature alone did not affect mortality. The mortalities in the exposed groups at 21 °C occurring earlier than 27 °C might suggest that 21 °C could be the optimum condition for the growth of *V*. *cyclitrophicus*. Furthermore, *V*. *cyclitrophicus* exposed groups at 27 °C may cause unfavourable physiological conditions of mussels, which resulted in a significant increase in mortality on day 9. A previous study in oyster showed that elevated temperature increased oyster mortality after *Vibrio* strain injection^8^. However, it should be noted that the injection does not reflect the natural route of infection compared to immersion. The normal route of exposure of mussels to bacteria and pathogens is through natural filtering. Many bivalves can close their shell and stop filtering when exposed to high concentrations of pathogenic bacteria. Besides, the increment of energy consumption caused by the combined effects of higher temperature and bacterial infection could influence the immune defence of the host^29^.

Our data revealed that Proteobacteria, Epsilonbacteraeota and Bacteroidetes were dominant in mussel haemolymph, and this was consistent with previous results of in oyster haemolymph^8^. Proteobacteria was abundantly found in the haemolymph of marine invertebrates such as Atlantic blue crab (*Callinectes sapidus*)^30^, Pacific oysters (*C*. *gigas*)^8^ and in the Coelomic fluid (equivalent to the haemolymph) of starfish (*Patiria pectinifera* and *Asterias amurensis*)^31^. The Epsilonbacteraeota (ε - Proteobacteria) was rarely present in coastal seawater^32,33^ and oyster gill microbiota^34^. Epsilonproteobacteria is a dominant symbiont, usually living in the shrimp *Rimicaris exoculata* from hydrothermal vents^35^. Therefore, high abundance of Epsilonbacteraeota harbour in mussel *M*. *coruscus* haemolymph might suggest that it represents the symbiotic bacterial populations maintaining homeostasis.

Microbial community stability can be identified as the persistence of populations over time, which is vital for community functioning^36^. Generally, the host is capable of balancing community composition to achieve stability^36,37^. Lower microbial diversity coincided with few dominant strains in unhealthy oysters and was largely affected by heat stress, while infection did not show an apparent effect^8^. Similarly, our study showed that elevated temperature rather than infection impacted microbial diversity in the mussel haemolymph. Resident microbes may be involved in host protection by competing with pathogen colonization through direct participation in the host immune system^11,38,39^. Excessive levels of interaction disturbances among environment, host and microbiota may destabilize the equilibrium and lead to shifting to alternative stable states or even lethal consequences for the host^7,36^.

In the present study, PCoA analysis revealed that the temperature is a crucial factor influencing microbial community that separated the treatment groups at 21 °C with treatment groups at 27 °C. A clustering of treatment groups at 27 °C was largely separated with the mussels at 21 °C suggested that the disturbance of microbial community may be an indicator of unhealthy status. This was consistent with the previous study in the oyster which demonstrated that microbial dynamics and community composition in haemolymph were altered by heat stress and infection, thus resulting in high mortality rate^8^. Furthermore, CCA analysis showed that the temperature and infection significantly affected the haemolymph microbiome of *M*. *coruscus*. Clearly, our data showed that higher temperature destabilized the haemolymph community.

The high abundance of *Pseudomonas* and *Bacillus* spp. strains in warm temperature exposed mussels may suggest an important role of the microbiome buffering the influence of temperature elevation. *Pseudomonas* spp. (*P*. *synxantha* and *P*. *aeruginosa*) has been used as probiotics supplement in the formulated feed, which showed an improvement of healthier effect in the haemolymph of juvenile western king prawns^40^. The majority species of *Bacillus* are harmlessness, and they are used as probiotics in shrimp aquaculture for improving growth performance and disease protection^41,42^. On the other hand, an increase of antibacterial activity was found in shrimp haemolymph when fed a species of *Bacillus*^41^. Previous report in oyster have shown that the higher abundances of *Arcobacter* species may act as opportunistic pathogens in haemolymph and the high densities of *Arcobacter* could be a contributing factor causing mortality^8^. The abundances of *Arcobacter* shifted in response to infection at 21 °C, possibly reflecting the decline of health. In the giant abalone (*Haliotis gigantea*) farm, high mass mortality accounting for 84% of the cumulative mortality rate was identified as the results of infection by a *Francisella* strain in the haemolymph^43^. The relative abundance of *Francisella* was high in treatment groups at 21 °C, suggesting the unhealthy status of the host. The haemolymph of various healthy marine organisms is the natural habitat of the vibrios population^5,44,45^. However, a high abundance of *Vibrio* present in live and dead treatment groups at 27 °C may contribute greatly to mortality, as indicated by LEfSe analysis. The visceral tissue in bivalves can eliminate invading microorganisms through an active phagocytic process conducted by haemocytes^5,46^. Thus, the proliferation of vibrios population might suggest that the elimination mechanisms are ineffective or the community shifts towards an alternative stable state^36^.

Our study showed that (i) elevated temperature together with exposure to *V*. *cyclitrophicus* led to high mortality, (ii) infection promoted the proliferation of opportunistic pathogens (e.g., *Arcobacter* and *Francisella*) at a lower temperature, (iii) elevated temperature might reduce the ability of bacterial elimination function against infection in the haemolymph. Taken together, the dynamics of microbial community and unique biomarker species in mussel haemolymph could be used as health indicators under changing environments.

## Material and Methods

### Ethics statement

The mussel acclimation and experimentation was approved by the Animal Ethics Committee of Shanghai Ocean University, China.

### Biological material

Adult *M*. *coruscus* were collected from Gouqi Island (30°72′N; 122°77′E), Zhoushan, Zhejiang Province, China, on October 2018. Five hundred mussels were immediately transferred to the laboratory, and kept in 10 L polycarbonate tanks (30 mussels/tank). The tanks were filled with seawater (salinity: 30), and the mussels were rinsed in seawater at 21 °C (the average seawater temperature at the collection site) to remove materials attached on the shell surfaces. Food source for mussels was the same as described previously^16^. The seawater in the tanks was replaced by seawater collected from the mussel’s habitat (Zhoushan, China) every other day. The mussels were kept for one week acclimation before the start of the experiment at 21 °C. The switch of temperature from 21 to 27 °C was achieved by gradually increasing with a rate of 1 °C/day in the incubators (Sanyo, Japan) for minimizing heat shock^47^. The average shell length and width of the mussels used in this experiment were 9.7 ± 0.52 cm and 4.7 ± 0.27 cm, respectively.

We infected the mussels using bacteria *V*. *cyclitrophicus* isolated from the marine biofilms in the natural habitat of *M*. *coruscus* at Gouqi Island (30°72′N; 122°77′E), Zhoushan, Zhejiang Province, China. This isolate showed low inductive activity for larval settlement and metamorphosis of *M*. *coruscus* (<20%) and mortality occurs after induction. The pellets of bacteria were obtained by centrifugation for 15 min at 1300 *g*, and the remaining culture medium was removed by rinsing three times with autoclaved filtered seawater (AFSW).

### Experimental setup and haemolymph sampling

The experiment was conducted to investigate the effects of temperature and infection on the mussel *M*. *coruscus* for nine days. A total of 62 mussels for each treatment groups were kept in duplicate 10 L polycarbonate tanks at 21 ± 1 °C and 27 ± 1 °C and fed daily with cell density of 8 × 10^4^ cells/mL algae. Two treatment groups included: (i) the control mussels at 21 and 27 °C (CTR) and (ii) the mussels exposed to the bacteria, *V*. *cyclitrophicus* at 21 and 27 °C (Table 1). The CTR groups were sampled at day 0 of the trial (9 individuals) to assess the microbiota at the beginning of the experiment. In the other treatment groups, the mussels were exposed to *V*. *cyclitrophicus* which was added to the filtered sterile seawater in the tank (a final concentration of 10^8^ CFU in the seawater tank). During the experiment, six mussels were collected from two tanks (3 mussels per tank) for haemolymph microbiome identification on day 8 and day 9, respectively. The chosen sampling intervals were made in function of the pre-experiment since it showed that high mortality of mussels occurred on day 9 at 27 °C challenged with *V*. *cyclitrophicus* (Fig. S3). The haemolymph was extracted from the adductor muscle by introducing a fine needle and gently withdrawing the fluid. The haemolymph used for bacterial DNA extraction were combined from pools of two individual mussels due to the low abundance of bacteria. Samples were immediately placed on ice and then stored at −80 °C.

**Table 1:** The experimental set-up for the mussel *M*. *coruscus*.

Assays	Temperature (°C)	Exposed	Sample time (day)	Mussel condition	
Control	21	Seawater	0	live	—
	27	Seawater	0	live	—
Treatment	21	*Vibro cyclitrophicus*	8	live	—
			9	live	—
	27	*Vibro cyclitrophicus*	8	live	—
			9	live	dead

### DNA extraction and PCR amplification

Total bacterial DNA was extracted (n = 3) using a MOBIO PowerSoil DNA Isolation Kit (MOBIO Laboratories, Carlsbad, CA, USA), following the manufacturer protocol. The DNA concentration and purity were measured using the NanoDrop One (Thermo Fisher Scientific, MA, USA). PCR amplifcation method referred to Li *et al*.^17^. The universal bacterial primers 338 F (5′-ACTCCTACGGGAGGCAGCA-3′) and 806 R (5′-GGACTACHVGGGTWTCTAAT-3′) with 12 bp barcode were used to amplify the 16S ribosomal RNA gene (V3-V4 region). PCR reactions, containing 25 μL 2x Premix Taq (Takara Biotechnology, Dalian Co. Ltd., China), 1 μL each primer (10 mM) and 3 μl DNA (20 ng/μL) template in a volume of 50 µL, were amplified by thermocycling: 5 min at 94 °C for initialization; 30 cycles of 30 s denaturation at 94 °C, 30 s annealing at 52 °C, and 30 s extension at 72 °C; followed by 10 min final elongation at 72 °C.

### Illumina HiSeq sequencing

The PCR products were detected by 1% agarose gel electrophoresis. PCR products were mixed in equidensity ratios according to the GeneTools analysis software (Version 4.03.05.0, SynGene). Mixture PCR products purified method and sequencing libraries construction was referred to Li *et al*.^17^. Raw sequences obtained in this study have been submitted to the NCBI database (SRP196510).

### Bioinformatics and statistical analysis

The bioinformatics analysis was referred to Li *et al*.^17^. Raw reads were filtered to remove low quality sequences (Base call quality value < 30), adaptors sequence, reads with N > 10% of the sequences and sequence < 150 bp long by Trimmomatic (V0.33, http://www.usadellab.org/cms/?page = trimmomatic). The filtered sequences were merged using FLASH (V1.2.11, https://ccb.jhu.edu/software/FLASH/) according to the relationship of the overlap between the paired-end reads. The sequences were assigned to each sample based on their unique barcodes and primers using Mothur software (V1.35.1, http://www.mothur.org) and followed by removing barcodes, ambiguous bases and primers to obtain the clean Tags. The Usearch software (Version 10.0. http://www.drive5.com/usearch/) was used to determine the (Operational Taxonomic Units, OTU), OTUs using a similarity threshold of 97%. Taxonomic annotation of 16S rRNA gene sequence was determined with the RDP classifier (http://rdp.cme.msu.edu/) against the database of Silva (Release132; http://www.arb-silva.de) with a confidence threshold of 0.5. A normalized (subsampled) OTU table was obtained based on the sample with the least sequences. The histogram, the richness index of Chao1, the diversity indices of Simpson’s and Shannon were performed by QIIME (V1.9.1) based on OTU table and displayed with R software (V2.15.3).

Heatmap was constructed showing 20 genera with significant differences in abundance between samples by R software. Principal Coordinate Analysis (PCoA) was performed to compare the changes of bacterial community between samples based on the unweighted UniFrac distances. The significant environmental variables were identified by Monte Carlo permutations (999 permutations with a *P* value <0.05). The differences of the relative abundance of bacteria between two or more groups were assessed by linear discriminant analysis effect size (LEfSe) analysis. The Kruskal-Wallis test identifies bacterial taxa that are significantly different in relative abundance among samples. The linear discriminant analysis (LDA) was used to identify the effect size with which these taxa differentiate among samples with thresholds of a log-transformed LDA score of 2.0.

The differences of microbial diversity indices (Chao1, Shannon and Simpson) among samples were firstly tested for normality (Shapiro-Wilk test) and homogeneity (O’Brien test)^16^. Wilcoxon/Kruskal-Wallis test has been applied when normality and homogeneity of Chao1, Shannon and Simpson were not satisfied. Data were analyzed for statistical significance using JMP software (SAS Institute, Shanghai, China) with a significance level set at 0.05.

## Supplementary information

Supplementary Figure
