# Supplementary material for: Temperature elevation and Vibrio cyclitrophicus infection reduce the diversity of haemolymph microbiome of the mussel Mytilus coruscus
Source: Sci Rep. 2019 Nov 8;9:16391. doi: 10.1038/s41598-019-52752-y (PMC6841970; doi:10.1038/s41598-019-52752-y)
Supplement: Supplementary file 1 — Supplementary Figure [file 41598_2019_52752_MOESM1_ESM.docx]

**Supplementary Information**

**Temperature elevation and *Vibrio cyclitrophicus* infection reduce the diversity of haemolymph microbiome of the mussel *Mytilus coruscus***

Yi-Feng Li^1,2,3+^, Yan-Wen Chen^1,2+^, Jia-Kang Xu^1,2^, Wen-Yang Ding^1,2^, An-Qi Shao^1,2^, You-Ting Zhu^1,2,3^, Chong Wang^4^, Xiao Liang^1,2,3*^, Jin-Long Yang^1,2,3*^

^a^ International Research Center for Marine Biosciences, Ministry of Science and Technology, Shanghai Ocean University, Shanghai, China

^b^ Key Laboratory of Exploration and Utilization of Aquatic Genetic Resources, Ministry of Education, Shanghai Ocean University, Shanghai, China

^c^ National Demonstration Center for Experimental Fisheries Science Education, Shanghai Ocean University, Shanghai, China

^d^ Ocean and Fisheries Research Institute of Binzhou, Binzhou, China

^*^Correspondence:

Xiao Liang, x-liang@shou.edu.cn

Address: College of Fisheries and Life Science, Shanghai Ocean University, 999 Hucheng Huan Road, Shanghai 201306, China

Jin-Long Yang, jlyang@shou.edu.cn

Address: College of Fisheries and Life Science, Shanghai Ocean University, 999 Hucheng Huan Road, Shanghai 201306, China

^+^ These authors contributed equally to this work.

**Supplementary Figures**

**
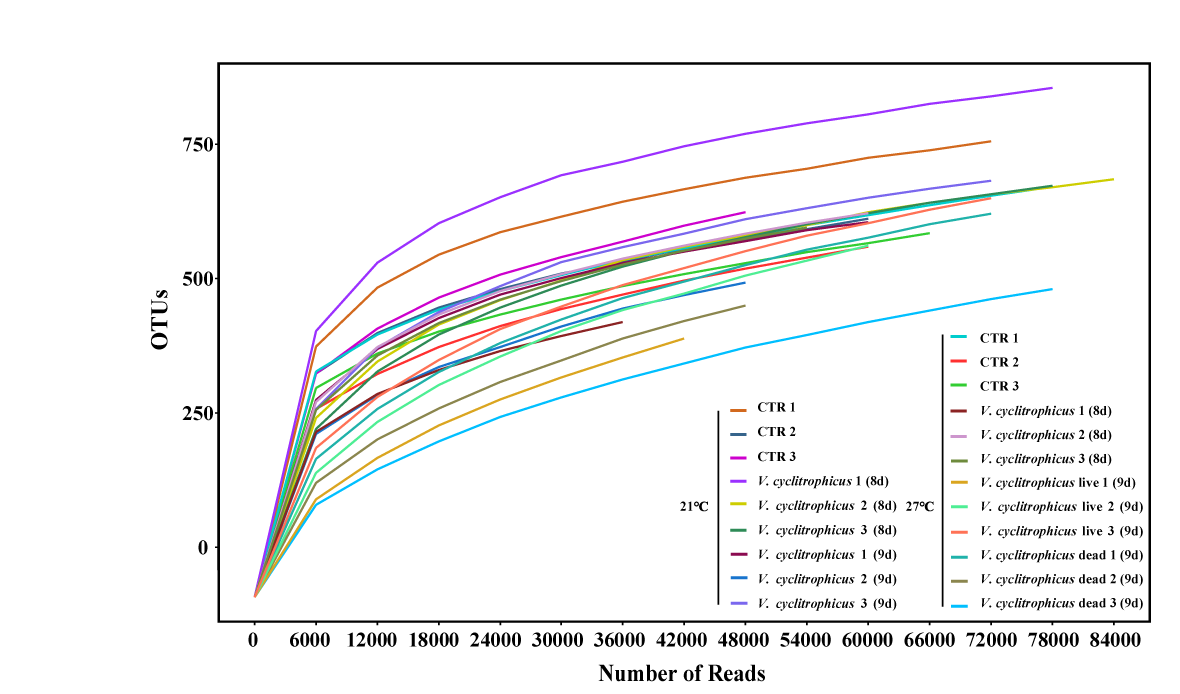
**

**Figure S1.** Observed species rarefaction curve. Different colours represent different groups, the horizontal axis represents the number of sequences, and the vertical axis represents the number of OTU.


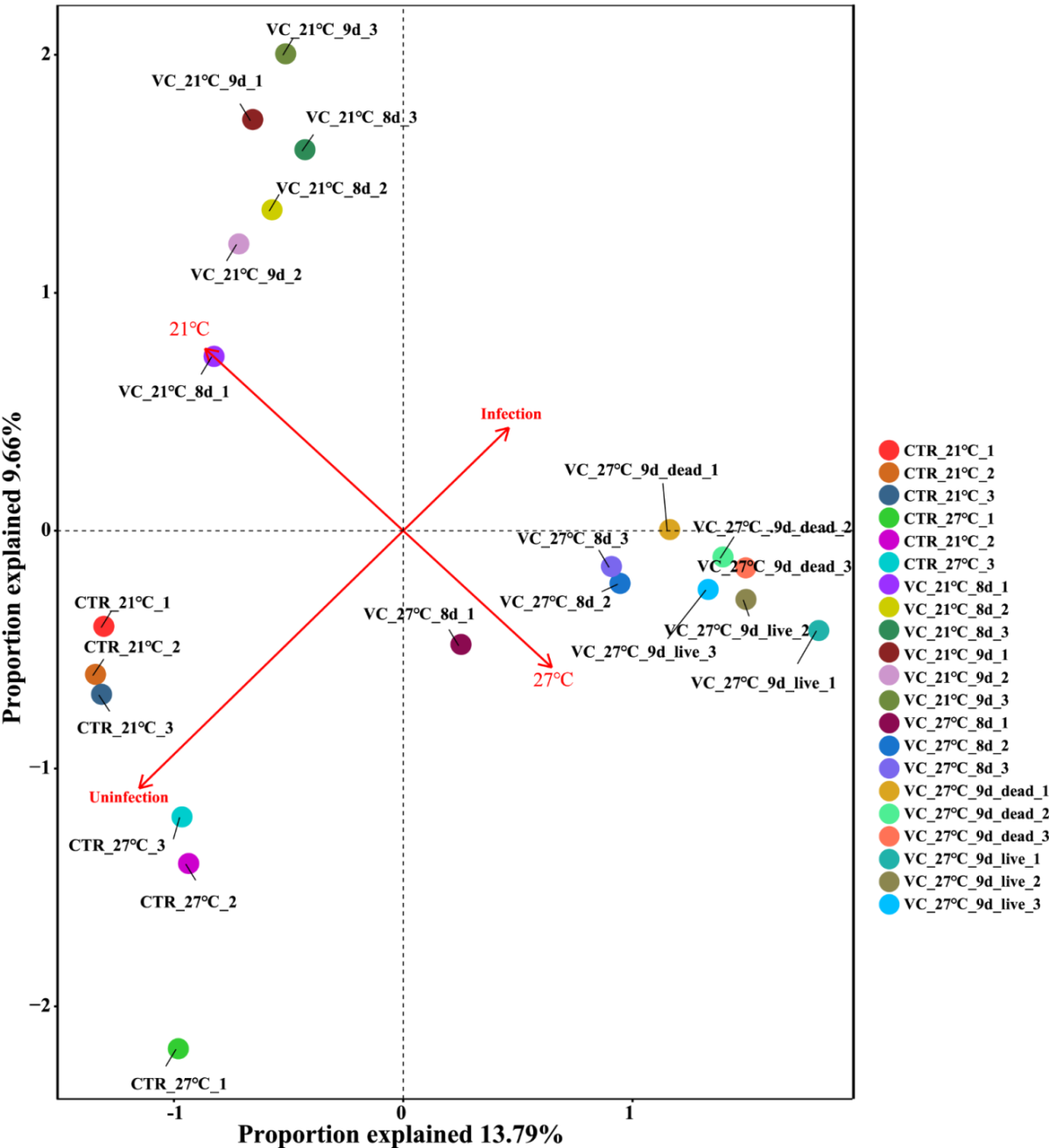


**Figure S2.** Canonical correspondence analysis (CCA) of the haemolymph microbiome data and environmental variables, including temperature and infection.

**
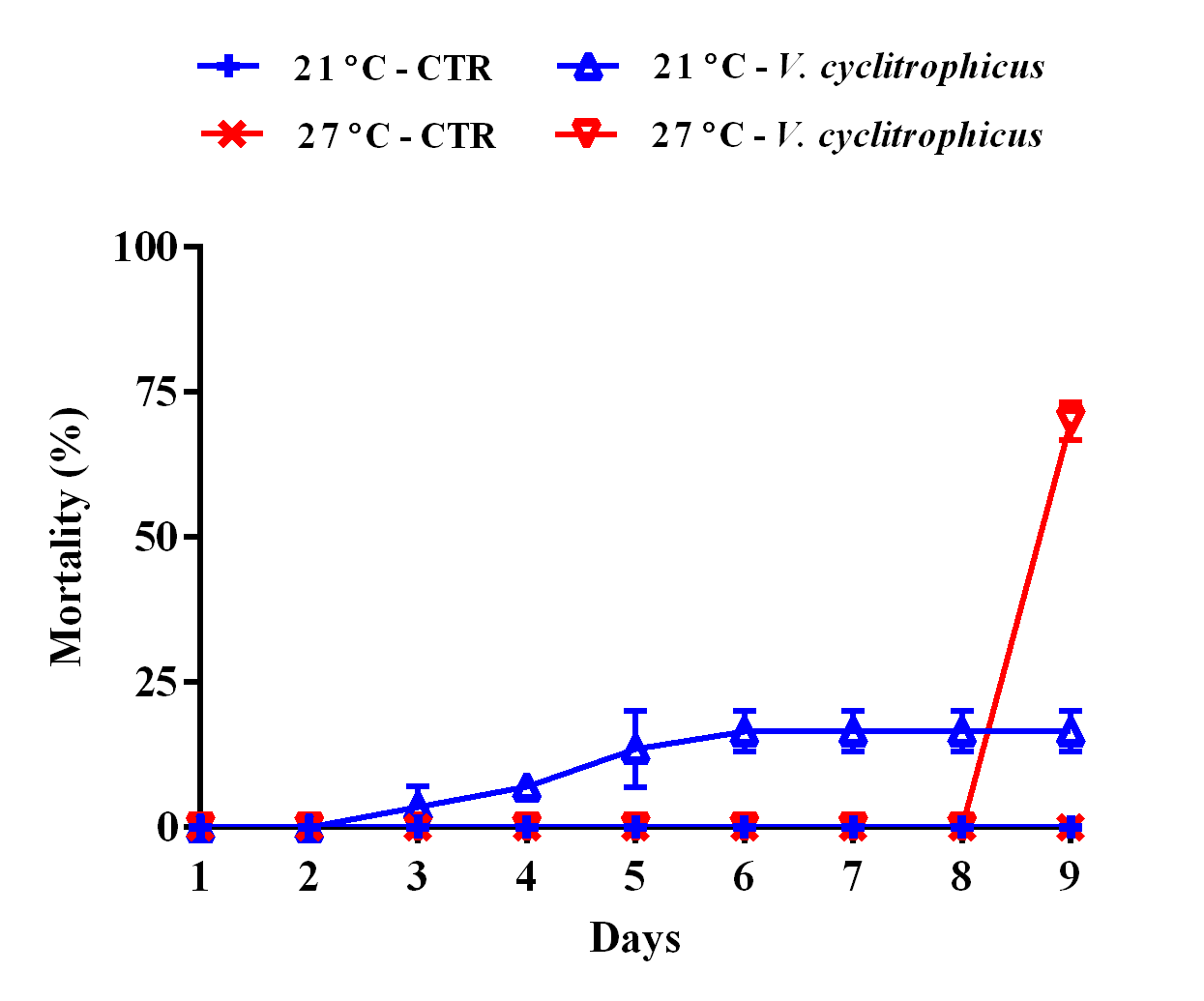
**

**Figure S3.** Mortality rates for *M. coruscus*. CTR: control groups.

**Supplementary Tables**

**Table S1. Relative abundance (in %) of the sequences of haemolymph assigned to different phyla.**

|  | **Live** | | | | | | | | | | | | | | | | | | **Dead** | | |
| --- | --- | --- | --- | --- | --- | --- | --- | --- | --- | --- | --- | --- | --- | --- | --- | --- | --- | --- | --- | --- | --- |
|  | **0d** | | | | | | **8d** | | | | | | **9d** | | | | | | | | |
|  | **21℃** | | | **27℃** | | | **21℃** | | | **27℃** | | | **21℃** | | | **27℃** | | | **27℃** | | |
| **Phylum** | **CTR.1** | **CTR.2** | **CTR.3** | **CTR.1** | **CTR.2** | **CTR.3** | **V. cyclitrophicus.1** | **V. cyclitrophicus.2** | **V. cyclitrophicus.3** | **V. cyclitrophicus.1** | **V. cyclitrophicus.2** | **V. cyclitrophicus.3** | **V. cyclitrophicus.1** | **V. cyclitrophicus.2** | **V. cyclitrophicus.3** | **V. cyclitrophicus.1** | **V. cyclitrophicus.2** | **V. cyclitrophicus.3** | **V. cyclitrophicus.1** | **V. cyclitrophicus.2** | **V. cyclitrophicus.3** |
| Acidobacteria | 0.02 | 0.05 | 0.03 | 0.63 | 0.21 | 0.26 | 0.34 | 0.14 | 0.02 | 0.01 | 0.11 | 0.01 | 0.06 | 0.02 | 0.01 | 0.02 | 0.02 | 0.05 | 0.01 | 0.02 | 0.01 |
| Actinobacteria | 0.35 | 0.89 | 0.70 | 5.83 | 0.72 | 0.33 | 1.11 | 0.09 | 0.08 | 0.24 | 0.06 | 0.06 | 0.19 | 0.30 | 0.14 | 0.02 | 0.02 | 0.02 | 0.05 | 0.02 | 0.01 |
| Armatimonadetes | 0.00 | 0.00 | 0.00 | 0.00 | 0.00 | 0.00 | 0.00 | 0.00 | 0.00 | 0.00 | 0.02 | 0.00 | 0.00 | 0.00 | 0.00 | 0.00 | 0.00 | 0.00 | 0.00 | 0.00 | 0.00 |
| BRC1 | 0.00 | 0.00 | 0.00 | 0.00 | 0.00 | 0.00 | 0.00 | 0.00 | 0.01 | 0.00 | 0.00 | 0.00 | 0.00 | 0.00 | 0.00 | 0.00 | 0.00 | 0.00 | 0.00 | 0.00 | 0.00 |
| Bacteroidetes | 30.20 | 20.29 | 17.77 | 13.36 | 10.78 | 15.63 | 13.14 | 40.49 | 42.33 | 24.22 | 27.78 | 27.57 | 15.52 | 9.95 | 23.37 | 2.66 | 41.84 | 22.34 | 29.52 | 22.31 | 7.82 |
| Caldiserica | 0.01 | 0.00 | 0.01 | 0.01 | 0.01 | 0.01 | 0.00 | 0.01 | 0.00 | 0.00 | 0.00 | 0.01 | 0.00 | 0.01 | 0.01 | 0.01 | 0.01 | 0.00 | 0.00 | 0.01 | 0.00 |
| Chlamydiae | 0.40 | 0.35 | 0.21 | 0.35 | 0.03 | 0.70 | 0.23 | 0.12 | 0.07 | 0.22 | 0.08 | 0.14 | 0.50 | 0.31 | 0.87 | 0.02 | 0.02 | 0.04 | 0.06 | 0.04 | 0.01 |
| Chloroflexi | 0.04 | 0.03 | 0.03 | 2.36 | 0.19 | 0.29 | 0.83 | 0.01 | 0.01 | 0.05 | 0.05 | 0.03 | 0.06 | 0.03 | 0.03 | 0.03 | 0.03 | 0.05 | 0.03 | 0.02 | 0.02 |
| Cyanobacteria | 0.29 | 0.11 | 0.07 | 0.98 | 0.01 | 0.01 | 0.31 | 0.03 | 0.18 | 0.00 | 0.05 | 0.06 | 0.04 | 0.09 | 0.04 | 0.01 | 0.00 | 0.01 | 0.01 | 0.01 | 0.00 |
| Deinococcus-Thermus | 0.00 | 0.05 | 0.05 | 0.00 | 0.00 | 0.09 | 0.00 | 0.00 | 0.00 | 0.00 | 0.00 | 0.00 | 0.00 | 0.00 | 0.05 | 0.00 | 0.00 | 0.00 | 0.00 | 0.00 | 0.00 |
| Dependentiae | 0.34 | 0.17 | 0.24 | 0.35 | 0.19 | 0.34 | 0.01 | 0.01 | 0.02 | 0.12 | 0.03 | 0.09 | 0.04 | 0.03 | 0.21 | 0.01 | 0.01 | 0.01 | 0.04 | 0.00 | 0.01 |
| Elusimicrobia | 0.00 | 0.00 | 0.00 | 0.00 | 0.00 | 0.00 | 0.00 | 0.00 | 0.00 | 0.00 | 0.00 | 0.00 | 0.00 | 0.00 | 0.00 | 0.00 | 0.00 | 0.00 | 0.00 | 0.00 | 0.00 |
| Entotheonellaeota | 0.00 | 0.00 | 0.00 | 0.00 | 0.00 | 0.00 | 0.03 | 0.00 | 0.00 | 0.00 | 0.00 | 0.00 | 0.00 | 0.00 | 0.00 | 0.00 | 0.00 | 0.00 | 0.00 | 0.00 | 0.00 |
| Epsilonbacteraeota | 14.83 | 8.05 | 11.54 | 0.58 | 1.34 | 0.95 | 5.99 | 0.82 | 3.83 | 1.74 | 17.07 | 13.50 | 14.67 | 22.16 | 15.38 | 1.47 | 10.44 | 10.71 | 8.84 | 10.59 | 13.42 |
| Fibrobacteres | 0.01 | 0.00 | 0.05 | 0.25 | 0.04 | 0.00 | 0.00 | 0.00 | 0.00 | 0.00 | 0.00 | 0.04 | 0.00 | 0.00 | 0.02 | 0.00 | 0.00 | 0.00 | 0.00 | 0.00 | 0.00 |
| Firmicutes | 1.67 | 3.90 | 5.88 | 15.54 | 12.38 | 6.72 | 6.54 | 1.50 | 1.00 | 3.53 | 3.40 | 1.34 | 2.67 | 2.31 | 0.85 | 3.56 | 1.09 | 3.15 | 1.70 | 1.22 | 0.29 |
| Fusobacteria | 1.15 | 0.41 | 0.73 | 0.10 | 0.26 | 0.33 | 0.19 | 0.24 | 11.09 | 0.11 | 0.32 | 1.67 | 0.30 | 0.16 | 0.26 | 0.63 | 0.78 | 0.58 | 8.42 | 0.25 | 48.40 |
| GAL15 | 0.00 | 0.00 | 0.00 | 0.17 | 0.00 | 0.00 | 0.00 | 0.00 | 0.00 | 0.00 | 0.00 | 0.00 | 0.00 | 0.00 | 0.00 | 0.00 | 0.00 | 0.00 | 0.00 | 0.00 | 0.00 |
| Gemmatimonadetes | 0.01 | 0.00 | 0.02 | 0.00 | 0.02 | 0.01 | 0.05 | 0.00 | 0.01 | 0.00 | 0.01 | 0.01 | 0.02 | 0.02 | 0.01 | 0.02 | 0.01 | 0.02 | 0.00 | 0.01 | 0.01 |
| Hydrogenedentes | 0.00 | 0.00 | 0.00 | 0.00 | 0.00 | 0.00 | 0.01 | 0.00 | 0.00 | 0.00 | 0.00 | 0.00 | 0.00 | 0.00 | 0.00 | 0.00 | 0.00 | 0.00 | 0.00 | 0.00 | 0.00 |
| Latescibacteria | 0.00 | 0.00 | 0.00 | 0.00 | 0.00 | 0.00 | 0.00 | 0.00 | 0.00 | 0.00 | 0.00 | 0.00 | 0.00 | 0.00 | 0.00 | 0.00 | 0.00 | 0.00 | 0.00 | 0.00 | 0.00 |
| Lentisphaerae | 0.00 | 0.00 | 0.00 | 0.00 | 0.03 | 0.00 | 0.00 | 0.00 | 0.00 | 0.00 | 0.00 | 0.00 | 0.00 | 0.00 | 0.00 | 0.00 | 0.00 | 0.00 | 0.00 | 0.00 | 0.00 |
| Margulisbacteria | 0.01 | 0.00 | 0.00 | 0.00 | 0.00 | 0.00 | 0.00 | 0.00 | 0.00 | 0.00 | 0.00 | 0.00 | 0.00 | 0.00 | 0.00 | 0.00 | 0.00 | 0.00 | 0.00 | 0.00 | 0.00 |
| Nitrospinae | 0.00 | 0.00 | 0.00 | 0.00 | 0.00 | 0.00 | 0.00 | 0.00 | 0.00 | 0.00 | 0.00 | 0.00 | 0.02 | 0.00 | 0.05 | 0.00 | 0.00 | 0.00 | 0.00 | 0.00 | 0.00 |
| Nitrospirae | 0.01 | 0.01 | 0.01 | 0.22 | 0.00 | 0.00 | 0.03 | 0.00 | 0.00 | 0.00 | 0.02 | 0.01 | 0.00 | 0.01 | 0.00 | 0.01 | 0.00 | 0.00 | 0.01 | 0.00 | 0.00 |
| Omnitrophicaeota | 0.00 | 0.00 | 0.00 | 0.00 | 0.00 | 0.00 | 0.00 | 0.00 | 0.00 | 0.00 | 0.00 | 0.00 | 0.00 | 0.00 | 0.00 | 0.00 | 0.00 | 0.00 | 0.00 | 0.00 | 0.00 |
| Patescibacteria | 1.03 | 1.43 | 1.34 | 1.44 | 0.54 | 1.52 | 2.12 | 13.90 | 8.59 | 0.40 | 0.60 | 0.73 | 6.96 | 2.58 | 17.59 | 0.12 | 0.16 | 0.59 | 1.30 | 0.49 | 0.09 |
| Planctomycetes | 0.04 | 0.03 | 0.08 | 0.05 | 0.34 | 0.19 | 0.22 | 0.05 | 0.09 | 0.01 | 0.19 | 0.15 | 0.02 | 0.10 | 0.05 | 0.01 | 0.03 | 0.03 | 0.02 | 0.02 | 0.01 |
| Proteobacteria | 48.77 | 62.78 | 60.13 | 56.82 | 72.63 | 71.78 | 68.20 | 40.96 | 27.38 | 68.80 | 47.92 | 52.04 | 58.34 | 61.66 | 40.55 | 91.24 | 45.30 | 61.92 | 48.99 | 64.60 | 29.73 |
| Spirochaetes | 0.02 | 0.00 | 0.02 | 0.33 | 0.03 | 0.05 | 0.07 | 0.00 | 0.02 | 0.06 | 0.02 | 0.01 | 0.01 | 0.00 | 0.02 | 0.07 | 0.01 | 0.01 | 0.64 | 0.19 | 0.03 |
| Synergistetes | 0.00 | 0.00 | 0.08 | 0.00 | 0.00 | 0.00 | 0.00 | 0.00 | 0.00 | 0.00 | 0.00 | 0.00 | 0.00 | 0.00 | 0.00 | 0.00 | 0.00 | 0.00 | 0.00 | 0.00 | 0.00 |
| Tenericutes | 0.13 | 0.29 | 0.08 | 0.08 | 0.09 | 0.15 | 0.08 | 0.02 | 0.03 | 0.03 | 0.02 | 0.02 | 0.02 | 0.06 | 0.02 | 0.05 | 0.04 | 0.03 | 0.03 | 0.03 | 0.03 |
| Verrucomicrobia | 0.44 | 0.35 | 0.15 | 0.38 | 0.07 | 0.38 | 0.28 | 1.53 | 4.30 | 0.42 | 2.03 | 2.38 | 0.37 | 0.19 | 0.40 | 0.05 | 0.16 | 0.39 | 0.27 | 0.15 | 0.09 |
| WPS-2 | 0.05 | 0.01 | 0.11 | 0.00 | 0.00 | 0.17 | 0.07 | 0.05 | 0.11 | 0.00 | 0.13 | 0.07 | 0.06 | 0.00 | 0.00 | 0.00 | 0.00 | 0.00 | 0.02 | 0.00 | 0.00 |
| Zixibacteria | 0.00 | 0.00 | 0.00 | 0.00 | 0.00 | 0.00 | 0.01 | 0.00 | 0.00 | 0.00 | 0.00 | 0.00 | 0.00 | 0.00 | 0.00 | 0.00 | 0.00 | 0.00 | 0.00 | 0.00 | 0.00 |
| Other | 0.20 | 0.80 | 0.71 | 0.18 | 0.07 | 0.06 | 0.13 | 0.03 | 0.80 | 0.01 | 0.10 | 0.07 | 0.14 | 0.00 | 0.07 | 0.01 | 0.03 | 0.03 | 0.02 | 0.00 | 0.01 |

**Table S2. Relative abundance (in %) of the sequences of haemolymph assigned to different genera.**

|  | **Live** | | | | | | | | | | | | | | | | | | **Dead** | | |
| --- | --- | --- | --- | --- | --- | --- | --- | --- | --- | --- | --- | --- | --- | --- | --- | --- | --- | --- | --- | --- | --- |
|  | **0d** | | | | | | **8d** | | | | | | **9d** | | | | | | | | |
|  | **21℃** | | | **27℃** | | | **21℃** | | | **27℃** | | | **21℃** | | | **27℃** | | | **27℃** | | |
| **Genus** | **CTR.1** | **CTR.2** | **CTR.3** | **CTR.1** | **CTR.2** | **CTR.3** | **V. cyclitrophicus.1** | **V. cyclitrophicus.2** | **V. cyclitrophicus.3** | **V. cyclitrophicus.1** | **V. cyclitrophicus.2** | **V. cyclitrophicus.3** | **V. cyclitrophicus.1** | **V. cyclitrophicus.2** | **V. cyclitrophicus.3** | **V. cyclitrophicus.1** | **V. cyclitrophicus.2** | **V. cyclitrophicus.3** | **V. cyclitrophicus.1** | **V. cyclitrophicus.2** | **V. cyclitrophicus.3** |
| Other | 9.30 | 9.24 | 6.86 | 6.01 | 4.29 | 8.02 | 5.53 | 23.36 | 11.56 | 16.66 | 12.29 | 16.02 | 10.52 | 7.61 | 19.73 | 1.79 | 19.18 | 22.58 | 23.76 | 20.72 | 5.69 |
| unclassified_Acidobacteriales | 0.00 | 0.00 | 0.00 | 0.00 | 0.00 | 0.00 | 0.00 | 0.00 | 0.00 | 0.00 | 0.00 | 0.00 | 0.00 | 0.00 | 0.00 | 0.00 | 0.00 | 0.00 | 0.00 | 0.00 | 0.00 |
| unclassified__Acidobacteriaceae_Subgroup_1 | 0.00 | 0.00 | 0.00 | 0.00 | 0.00 | 0.00 | 0.00 | 0.00 | 0.00 | 0.00 | 0.00 | 0.00 | 0.00 | 0.00 | 0.00 | 0.00 | 0.00 | 0.00 | 0.00 | 0.00 | 0.00 |
| Candidatus_Koribacter | 0.00 | 0.00 | 0.00 | 0.00 | 0.00 | 0.00 | 0.00 | 0.00 | 0.00 | 0.00 | 0.00 | 0.00 | 0.00 | 0.00 | 0.00 | 0.00 | 0.00 | 0.00 | 0.00 | 0.00 | 0.00 |
| Candidatus_Solibacter | 0.00 | 0.00 | 0.00 | 0.00 | 0.00 | 0.00 | 0.00 | 0.00 | 0.00 | 0.00 | 0.00 | 0.00 | 0.00 | 0.01 | 0.00 | 0.01 | 0.00 | 0.00 | 0.00 | 0.00 | 0.00 |
| Paludibaculum | 0.00 | 0.00 | 0.00 | 0.12 | 0.00 | 0.00 | 0.00 | 0.00 | 0.00 | 0.00 | 0.00 | 0.00 | 0.00 | 0.00 | 0.00 | 0.00 | 0.00 | 0.00 | 0.00 | 0.00 | 0.00 |
| unclassified_Acidobacteriia_Subgroup_2 | 0.00 | 0.00 | 0.01 | 0.00 | 0.00 | 0.00 | 0.00 | 0.00 | 0.00 | 0.00 | 0.00 | 0.00 | 0.00 | 0.00 | 0.00 | 0.00 | 0.00 | 0.00 | 0.00 | 0.00 | 0.00 |
| unclassified_Aminicenantales | 0.00 | 0.00 | 0.00 | 0.00 | 0.01 | 0.00 | 0.07 | 0.00 | 0.00 | 0.00 | 0.05 | 0.00 | 0.00 | 0.00 | 0.00 | 0.00 | 0.00 | 0.03 | 0.00 | 0.00 | 0.00 |
| Blastocatella | 0.01 | 0.04 | 0.00 | 0.15 | 0.19 | 0.25 | 0.00 | 0.12 | 0.01 | 0.00 | 0.04 | 0.00 | 0.02 | 0.00 | 0.00 | 0.00 | 0.00 | 0.01 | 0.00 | 0.00 | 0.00 |
| JGI_0001001-H03 | 0.00 | 0.00 | 0.00 | 0.00 | 0.00 | 0.00 | 0.00 | 0.00 | 0.00 | 0.00 | 0.00 | 0.00 | 0.00 | 0.00 | 0.00 | 0.00 | 0.00 | 0.00 | 0.00 | 0.00 | 0.00 |
| unclassified_Elev-16S-573 | 0.00 | 0.00 | 0.00 | 0.00 | 0.00 | 0.00 | 0.00 | 0.00 | 0.00 | 0.00 | 0.00 | 0.00 | 0.00 | 0.00 | 0.00 | 0.00 | 0.00 | 0.00 | 0.00 | 0.00 | 0.00 |
| RB41 | 0.01 | 0.00 | 0.01 | 0.00 | 0.00 | 0.00 | 0.00 | 0.00 | 0.00 | 0.00 | 0.00 | 0.00 | 0.00 | 0.00 | 0.00 | 0.01 | 0.00 | 0.00 | 0.00 | 0.00 | 0.00 |
| unclassified_Holophagae_Subgroup_7 | 0.00 | 0.00 | 0.00 | 0.00 | 0.00 | 0.00 | 0.00 | 0.00 | 0.00 | 0.00 | 0.00 | 0.00 | 0.00 | 0.00 | 0.00 | 0.00 | 0.00 | 0.00 | 0.00 | 0.00 | 0.00 |
| unclassified_Acidobacteria_Subgroup_17 | 0.00 | 0.00 | 0.00 | 0.00 | 0.00 | 0.00 | 0.01 | 0.00 | 0.00 | 0.00 | 0.00 | 0.00 | 0.00 | 0.01 | 0.00 | 0.00 | 0.00 | 0.00 | 0.00 | 0.00 | 0.00 |
| unclassified_Acidobacteria_Subgroup_18 | 0.00 | 0.00 | 0.00 | 0.00 | 0.00 | 0.00 | 0.02 | 0.00 | 0.00 | 0.00 | 0.00 | 0.00 | 0.00 | 0.00 | 0.00 | 0.00 | 0.00 | 0.00 | 0.00 | 0.00 | 0.00 |
| unclassified_Acidobacteria_Subgroup_21 | 0.00 | 0.00 | 0.00 | 0.00 | 0.00 | 0.00 | 0.02 | 0.00 | 0.00 | 0.00 | 0.00 | 0.00 | 0.00 | 0.00 | 0.00 | 0.00 | 0.00 | 0.00 | 0.00 | 0.00 | 0.00 |
| unclassified_Acidobacteria_Subgroup_22 | 0.00 | 0.00 | 0.00 | 0.00 | 0.00 | 0.00 | 0.04 | 0.00 | 0.00 | 0.00 | 0.00 | 0.00 | 0.00 | 0.00 | 0.00 | 0.00 | 0.00 | 0.00 | 0.00 | 0.00 | 0.00 |
| unclassified_Acidobacteria_Subgroup_5 | 0.00 | 0.00 | 0.00 | 0.00 | 0.00 | 0.00 | 0.07 | 0.00 | 0.00 | 0.00 | 0.00 | 0.00 | 0.00 | 0.00 | 0.00 | 0.00 | 0.00 | 0.00 | 0.00 | 0.00 | 0.00 |
| unclassified_Acidobacteria_Subgroup_6 | 0.00 | 0.00 | 0.00 | 0.35 | 0.00 | 0.00 | 0.03 | 0.00 | 0.00 | 0.00 | 0.00 | 0.00 | 0.02 | 0.00 | 0.00 | 0.00 | 0.00 | 0.00 | 0.00 | 0.00 | 0.00 |
| unclassified_Thermoanaerobaculaceae_Subgroup_10 | 0.00 | 0.00 | 0.00 | 0.00 | 0.00 | 0.00 | 0.03 | 0.00 | 0.00 | 0.00 | 0.00 | 0.00 | 0.00 | 0.00 | 0.00 | 0.00 | 0.00 | 0.00 | 0.00 | 0.00 | 0.00 |
| unclassified_Thermoanaerobaculaceae_Subgroup_23 | 0.00 | 0.00 | 0.00 | 0.00 | 0.00 | 0.00 | 0.05 | 0.00 | 0.00 | 0.00 | 0.00 | 0.00 | 0.00 | 0.00 | 0.00 | 0.00 | 0.00 | 0.00 | 0.00 | 0.00 | 0.00 |
| unclassified_Actinomarinales | 0.03 | 0.00 | 0.00 | 0.00 | 0.00 | 0.00 | 0.23 | 0.00 | 0.01 | 0.00 | 0.00 | 0.00 | 0.00 | 0.00 | 0.00 | 0.00 | 0.00 | 0.00 | 0.01 | 0.00 | 0.00 |
| unclassified_IMCC26256 | 0.00 | 0.00 | 0.00 | 0.00 | 0.00 | 0.00 | 0.02 | 0.00 | 0.00 | 0.00 | 0.00 | 0.00 | 0.00 | 0.00 | 0.00 | 0.00 | 0.00 | 0.00 | 0.00 | 0.00 | 0.00 |
| unclassified_Microtrichales | 0.00 | 0.00 | 0.00 | 0.33 | 0.00 | 0.00 | 0.00 | 0.00 | 0.00 | 0.00 | 0.00 | 0.00 | 0.00 | 0.00 | 0.00 | 0.00 | 0.00 | 0.00 | 0.00 | 0.00 | 0.00 |
| bacterium_YC-LK-LKJ22 | 0.00 | 0.00 | 0.00 | 0.00 | 0.00 | 0.00 | 0.01 | 0.00 | 0.00 | 0.00 | 0.00 | 0.00 | 0.00 | 0.00 | 0.00 | 0.00 | 0.00 | 0.00 | 0.00 | 0.00 | 0.00 |
| Iamia | 0.01 | 0.00 | 0.00 | 0.00 | 0.00 | 0.00 | 0.00 | 0.00 | 0.00 | 0.00 | 0.00 | 0.00 | 0.00 | 0.00 | 0.00 | 0.00 | 0.00 | 0.00 | 0.00 | 0.00 | 0.00 |
| Ilumatobacter | 0.00 | 0.00 | 0.00 | 0.00 | 0.00 | 0.00 | 0.01 | 0.00 | 0.00 | 0.00 | 0.00 | 0.00 | 0.00 | 0.00 | 0.00 | 0.00 | 0.00 | 0.00 | 0.00 | 0.00 | 0.00 |
| unclassified_Microtrichaceae | 0.05 | 0.08 | 0.00 | 0.00 | 0.00 | 0.00 | 0.04 | 0.00 | 0.02 | 0.00 | 0.01 | 0.00 | 0.06 | 0.00 | 0.00 | 0.00 | 0.00 | 0.00 | 0.01 | 0.00 | 0.00 |
| Sva0996_marine_group | 0.01 | 0.11 | 0.26 | 0.00 | 0.00 | 0.00 | 0.09 | 0.01 | 0.00 | 0.00 | 0.00 | 0.00 | 0.00 | 0.01 | 0.00 | 0.00 | 0.00 | 0.00 | 0.00 | 0.00 | 0.00 |
| Actinomyces | 0.00 | 0.06 | 0.02 | 0.06 | 0.00 | 0.00 | 0.05 | 0.00 | 0.00 | 0.00 | 0.00 | 0.00 | 0.01 | 0.00 | 0.00 | 0.00 | 0.00 | 0.00 | 0.00 | 0.00 | 0.00 |
| Bifidobacterium | 0.02 | 0.00 | 0.00 | 0.00 | 0.00 | 0.00 | 0.00 | 0.00 | 0.00 | 0.00 | 0.00 | 0.00 | 0.00 | 0.00 | 0.00 | 0.00 | 0.00 | 0.00 | 0.00 | 0.00 | 0.00 |
| Gardnerella | 0.00 | 0.00 | 0.00 | 0.00 | 0.00 | 0.00 | 0.01 | 0.00 | 0.00 | 0.00 | 0.00 | 0.00 | 0.00 | 0.00 | 0.00 | 0.00 | 0.00 | 0.00 | 0.00 | 0.00 | 0.00 |
| Corynebacterium | 0.00 | 0.02 | 0.00 | 0.00 | 0.00 | 0.00 | 0.00 | 0.00 | 0.00 | 0.00 | 0.00 | 0.00 | 0.00 | 0.00 | 0.01 | 0.00 | 0.00 | 0.00 | 0.00 | 0.00 | 0.00 |
| Corynebacterium_1 | 0.00 | 0.00 | 0.08 | 0.07 | 0.05 | 0.00 | 0.02 | 0.00 | 0.00 | 0.03 | 0.00 | 0.00 | 0.01 | 0.09 | 0.10 | 0.00 | 0.00 | 0.00 | 0.00 | 0.00 | 0.00 |
| Lawsonella | 0.00 | 0.04 | 0.00 | 0.00 | 0.00 | 0.00 | 0.00 | 0.00 | 0.00 | 0.00 | 0.00 | 0.00 | 0.00 | 0.00 | 0.00 | 0.00 | 0.00 | 0.00 | 0.00 | 0.00 | 0.00 |
| Dietzia | 0.00 | 0.02 | 0.00 | 0.00 | 0.00 | 0.00 | 0.00 | 0.00 | 0.00 | 0.00 | 0.00 | 0.00 | 0.00 | 0.00 | 0.00 | 0.00 | 0.00 | 0.00 | 0.00 | 0.00 | 0.00 |
| Mycobacterium | 0.00 | 0.01 | 0.00 | 0.00 | 0.00 | 0.00 | 0.00 | 0.00 | 0.00 | 0.00 | 0.00 | 0.00 | 0.00 | 0.04 | 0.00 | 0.00 | 0.00 | 0.00 | 0.00 | 0.00 | 0.00 |
| Gordonia | 0.00 | 0.00 | 0.00 | 0.00 | 0.00 | 0.00 | 0.00 | 0.00 | 0.00 | 0.00 | 0.00 | 0.00 | 0.00 | 0.00 | 0.00 | 0.00 | 0.00 | 0.00 | 0.00 | 0.00 | 0.00 |
| Nocardia | 0.00 | 0.00 | 0.00 | 0.00 | 0.00 | 0.00 | 0.02 | 0.00 | 0.00 | 0.00 | 0.00 | 0.00 | 0.00 | 0.00 | 0.00 | 0.00 | 0.00 | 0.00 | 0.00 | 0.00 | 0.00 |
| Rhodococcus | 0.00 | 0.00 | 0.00 | 0.03 | 0.00 | 0.01 | 0.00 | 0.00 | 0.00 | 0.00 | 0.00 | 0.00 | 0.01 | 0.00 | 0.00 | 0.00 | 0.00 | 0.00 | 0.00 | 0.00 | 0.00 |
| unclassified_Frankiales | 0.00 | 0.10 | 0.00 | 0.00 | 0.00 | 0.00 | 0.00 | 0.00 | 0.00 | 0.00 | 0.00 | 0.00 | 0.00 | 0.00 | 0.00 | 0.00 | 0.00 | 0.00 | 0.00 | 0.00 | 0.00 |
| Acidothermus | 0.00 | 0.00 | 0.01 | 0.00 | 0.00 | 0.00 | 0.00 | 0.00 | 0.00 | 0.00 | 0.00 | 0.00 | 0.00 | 0.00 | 0.00 | 0.00 | 0.00 | 0.00 | 0.00 | 0.00 | 0.00 |
| Fodinicola | 0.00 | 0.00 | 0.00 | 0.13 | 0.00 | 0.00 | 0.00 | 0.00 | 0.00 | 0.00 | 0.00 | 0.00 | 0.00 | 0.00 | 0.00 | 0.00 | 0.00 | 0.00 | 0.00 | 0.00 | 0.00 |
| Blastococcus | 0.01 | 0.00 | 0.00 | 0.00 | 0.00 | 0.00 | 0.00 | 0.00 | 0.00 | 0.00 | 0.00 | 0.00 | 0.00 | 0.00 | 0.00 | 0.00 | 0.00 | 0.00 | 0.00 | 0.00 | 0.00 |
| Geodermatophilus | 0.00 | 0.00 | 0.00 | 0.00 | 0.00 | 0.01 | 0.00 | 0.00 | 0.00 | 0.00 | 0.00 | 0.00 | 0.00 | 0.00 | 0.00 | 0.00 | 0.00 | 0.00 | 0.00 | 0.00 | 0.00 |
| hgcI_clade | 0.00 | 0.00 | 0.00 | 0.00 | 0.00 | 0.00 | 0.00 | 0.00 | 0.01 | 0.00 | 0.00 | 0.00 | 0.00 | 0.00 | 0.00 | 0.00 | 0.00 | 0.00 | 0.00 | 0.00 | 0.00 |
| Brevibacterium | 0.00 | 0.00 | 0.00 | 0.01 | 0.00 | 0.00 | 0.00 | 0.00 | 0.00 | 0.00 | 0.00 | 0.01 | 0.03 | 0.00 | 0.02 | 0.00 | 0.00 | 0.00 | 0.00 | 0.00 | 0.00 |
| Brachybacterium | 0.00 | 0.00 | 0.00 | 0.08 | 0.00 | 0.00 | 0.00 | 0.00 | 0.00 | 0.00 | 0.00 | 0.00 | 0.01 | 0.04 | 0.00 | 0.00 | 0.00 | 0.00 | 0.00 | 0.00 | 0.00 |
| Dermacoccus | 0.03 | 0.00 | 0.00 | 0.00 | 0.00 | 0.00 | 0.00 | 0.00 | 0.00 | 0.05 | 0.00 | 0.00 | 0.00 | 0.00 | 0.00 | 0.00 | 0.00 | 0.00 | 0.00 | 0.00 | 0.00 |
| Tetrasphaera | 0.00 | 0.00 | 0.00 | 0.45 | 0.00 | 0.00 | 0.02 | 0.00 | 0.00 | 0.00 | 0.00 | 0.00 | 0.00 | 0.00 | 0.00 | 0.00 | 0.00 | 0.00 | 0.00 | 0.00 | 0.00 |
| unclassified_Microbacteriaceae | 0.02 | 0.00 | 0.13 | 0.01 | 0.08 | 0.00 | 0.00 | 0.00 | 0.00 | 0.00 | 0.00 | 0.00 | 0.00 | 0.00 | 0.00 | 0.00 | 0.00 | 0.00 | 0.00 | 0.00 | 0.00 |
| Candidatus_Aquiluna | 0.00 | 0.00 | 0.00 | 0.00 | 0.07 | 0.00 | 0.00 | 0.00 | 0.00 | 0.00 | 0.00 | 0.00 | 0.00 | 0.00 | 0.00 | 0.00 | 0.00 | 0.00 | 0.00 | 0.00 | 0.00 |
| unclassified_Micrococcaceae | 0.02 | 0.23 | 0.05 | 0.16 | 0.35 | 0.00 | 0.03 | 0.00 | 0.00 | 0.00 | 0.00 | 0.00 | 0.00 | 0.02 | 0.00 | 0.01 | 0.00 | 0.00 | 0.00 | 0.00 | 0.00 |
| Garicola | 0.00 | 0.03 | 0.00 | 0.00 | 0.00 | 0.00 | 0.00 | 0.00 | 0.00 | 0.00 | 0.00 | 0.00 | 0.00 | 0.00 | 0.00 | 0.00 | 0.00 | 0.00 | 0.00 | 0.00 | 0.00 |
| Glutamicibacter | 0.00 | 0.00 | 0.00 | 0.48 | 0.00 | 0.07 | 0.14 | 0.01 | 0.00 | 0.00 | 0.00 | 0.00 | 0.00 | 0.00 | 0.00 | 0.00 | 0.00 | 0.00 | 0.00 | 0.00 | 0.00 |
| Kocuria | 0.00 | 0.01 | 0.05 | 0.00 | 0.00 | 0.00 | 0.00 | 0.00 | 0.00 | 0.00 | 0.02 | 0.02 | 0.00 | 0.00 | 0.00 | 0.00 | 0.00 | 0.00 | 0.00 | 0.00 | 0.00 |
| Micrococcus | 0.00 | 0.10 | 0.00 | 0.00 | 0.00 | 0.00 | 0.00 | 0.00 | 0.00 | 0.01 | 0.00 | 0.00 | 0.01 | 0.05 | 0.00 | 0.00 | 0.00 | 0.00 | 0.00 | 0.00 | 0.00 |
| Rothia | 0.01 | 0.02 | 0.00 | 0.17 | 0.04 | 0.00 | 0.04 | 0.00 | 0.00 | 0.03 | 0.00 | 0.00 | 0.00 | 0.00 | 0.00 | 0.00 | 0.00 | 0.00 | 0.00 | 0.00 | 0.00 |
| Micromonospora | 0.00 | 0.00 | 0.00 | 1.29 | 0.00 | 0.00 | 0.00 | 0.00 | 0.00 | 0.00 | 0.00 | 0.00 | 0.00 | 0.00 | 0.00 | 0.00 | 0.01 | 0.00 | 0.00 | 0.00 | 0.00 |
| unclassified_PeM15 | 0.08 | 0.02 | 0.00 | 0.00 | 0.03 | 0.06 | 0.00 | 0.00 | 0.00 | 0.00 | 0.00 | 0.00 | 0.00 | 0.00 | 0.00 | 0.00 | 0.00 | 0.00 | 0.00 | 0.00 | 0.00 |
| Marmoricola | 0.00 | 0.00 | 0.00 | 0.39 | 0.00 | 0.00 | 0.05 | 0.00 | 0.00 | 0.00 | 0.00 | 0.00 | 0.00 | 0.00 | 0.00 | 0.00 | 0.00 | 0.00 | 0.00 | 0.00 | 0.00 |
| Nocardioides | 0.00 | 0.00 | 0.01 | 0.05 | 0.00 | 0.00 | 0.01 | 0.00 | 0.00 | 0.00 | 0.00 | 0.00 | 0.02 | 0.00 | 0.00 | 0.00 | 0.00 | 0.00 | 0.00 | 0.00 | 0.00 |
| Cutibacterium | 0.00 | 0.01 | 0.01 | 0.00 | 0.06 | 0.06 | 0.01 | 0.00 | 0.00 | 0.00 | 0.00 | 0.00 | 0.00 | 0.02 | 0.00 | 0.00 | 0.00 | 0.00 | 0.00 | 0.00 | 0.00 |
| Propioniciclava | 0.00 | 0.00 | 0.00 | 0.00 | 0.00 | 0.00 | 0.03 | 0.00 | 0.00 | 0.00 | 0.00 | 0.00 | 0.00 | 0.00 | 0.00 | 0.00 | 0.00 | 0.00 | 0.00 | 0.00 | 0.00 |
| Actinomycetospora | 0.00 | 0.00 | 0.00 | 0.00 | 0.00 | 0.00 | 0.00 | 0.00 | 0.00 | 0.00 | 0.00 | 0.01 | 0.00 | 0.00 | 0.00 | 0.00 | 0.00 | 0.00 | 0.00 | 0.00 | 0.00 |
| Saccharopolyspora | 0.00 | 0.00 | 0.00 | 0.00 | 0.00 | 0.00 | 0.01 | 0.00 | 0.00 | 0.00 | 0.00 | 0.00 | 0.00 | 0.00 | 0.00 | 0.00 | 0.00 | 0.00 | 0.00 | 0.00 | 0.00 |
| unclassified_Streptomycetaceae | 0.02 | 0.03 | 0.06 | 0.25 | 0.00 | 0.12 | 0.04 | 0.05 | 0.00 | 0.08 | 0.02 | 0.00 | 0.03 | 0.01 | 0.00 | 0.00 | 0.00 | 0.00 | 0.01 | 0.00 | 0.00 |
| Nocardiopsis | 0.00 | 0.00 | 0.00 | 0.00 | 0.00 | 0.00 | 0.00 | 0.00 | 0.00 | 0.00 | 0.00 | 0.00 | 0.00 | 0.00 | 0.00 | 0.00 | 0.00 | 0.00 | 0.00 | 0.00 | 0.00 |
| unclassified_Coriobacteriales | 0.00 | 0.00 | 0.00 | 0.00 | 0.00 | 0.00 | 0.00 | 0.00 | 0.00 | 0.04 | 0.00 | 0.00 | 0.00 | 0.00 | 0.00 | 0.00 | 0.00 | 0.00 | 0.00 | 0.00 | 0.00 |
| Atopobium | 0.00 | 0.00 | 0.00 | 0.00 | 0.00 | 0.00 | 0.00 | 0.00 | 0.00 | 0.00 | 0.00 | 0.00 | 0.00 | 0.00 | 0.00 | 0.00 | 0.00 | 0.00 | 0.00 | 0.00 | 0.00 |
| unclassified_OPB41 | 0.00 | 0.00 | 0.00 | 0.41 | 0.00 | 0.00 | 0.04 | 0.00 | 0.00 | 0.00 | 0.00 | 0.00 | 0.00 | 0.00 | 0.00 | 0.00 | 0.00 | 0.00 | 0.00 | 0.00 | 0.00 |
| unclassified_MB-A2-108 | 0.00 | 0.00 | 0.00 | 0.46 | 0.00 | 0.00 | 0.00 | 0.00 | 0.00 | 0.00 | 0.00 | 0.00 | 0.00 | 0.00 | 0.00 | 0.00 | 0.00 | 0.00 | 0.00 | 0.00 | 0.00 |
| unclassified_Gaiellales | 0.00 | 0.00 | 0.00 | 0.32 | 0.00 | 0.00 | 0.09 | 0.00 | 0.00 | 0.00 | 0.00 | 0.00 | 0.00 | 0.00 | 0.00 | 0.00 | 0.00 | 0.00 | 0.00 | 0.00 | 0.00 |
| Gaiella | 0.00 | 0.00 | 0.00 | 0.32 | 0.00 | 0.00 | 0.00 | 0.00 | 0.00 | 0.00 | 0.00 | 0.00 | 0.00 | 0.00 | 0.00 | 0.00 | 0.00 | 0.00 | 0.00 | 0.00 | 0.00 |
| Fimbriimonas | 0.00 | 0.00 | 0.00 | 0.00 | 0.00 | 0.00 | 0.00 | 0.00 | 0.00 | 0.00 | 0.02 | 0.00 | 0.00 | 0.00 | 0.00 | 0.00 | 0.00 | 0.00 | 0.00 | 0.00 | 0.00 |
| unclassified_BRC1 | 0.00 | 0.00 | 0.00 | 0.00 | 0.00 | 0.00 | 0.00 | 0.00 | 0.01 | 0.00 | 0.00 | 0.00 | 0.00 | 0.00 | 0.00 | 0.00 | 0.00 | 0.00 | 0.00 | 0.00 | 0.00 |
| Bacteroides | 0.61 | 0.49 | 0.20 | 0.39 | 0.03 | 0.28 | 2.09 | 0.59 | 6.16 | 0.15 | 0.01 | 0.03 | 0.07 | 0.08 | 0.01 | 0.01 | 0.02 | 0.02 | 0.51 | 0.08 | 0.04 |
| unclassified_Bacteroidetes_BD2-2 | 0.00 | 0.00 | 0.00 | 0.00 | 0.03 | 0.00 | 0.03 | 0.00 | 0.00 | 0.00 | 0.00 | 0.00 | 0.00 | 0.00 | 0.00 | 0.00 | 0.00 | 0.00 | 0.00 | 0.00 | 0.00 |
| unclassified_Bacteroidetes_vadinHA17 | 0.01 | 0.01 | 0.03 | 0.01 | 0.02 | 0.01 | 0.02 | 0.01 | 0.01 | 0.01 | 0.01 | 0.02 | 0.01 | 0.02 | 0.01 | 0.02 | 0.02 | 0.01 | 0.02 | 0.02 | 0.02 |
| unclassified_Marinifilaceae | 6.41 | 2.26 | 2.63 | 1.46 | 0.41 | 1.46 | 0.16 | 0.21 | 1.32 | 0.10 | 0.13 | 0.14 | 0.17 | 0.08 | 0.17 | 0.11 | 0.72 | 1.10 | 2.24 | 0.43 | 0.26 |
| Marinifilum | 0.12 | 0.25 | 0.06 | 0.80 | 0.81 | 0.78 | 0.45 | 0.84 | 16.46 | 0.03 | 0.67 | 0.17 | 0.25 | 0.23 | 0.26 | 0.23 | 0.17 | 0.20 | 1.45 | 0.29 | 0.05 |
| Odoribacter | 0.00 | 0.00 | 0.00 | 0.00 | 0.00 | 0.00 | 0.00 | 0.00 | 0.00 | 0.00 | 0.00 | 0.00 | 0.00 | 0.03 | 0.00 | 0.00 | 0.00 | 0.00 | 0.00 | 0.00 | 0.00 |
| unclassified_Marinilabiliaceae | 0.00 | 0.00 | 0.00 | 0.00 | 0.00 | 0.00 | 0.00 | 0.00 | 0.00 | 0.00 | 0.00 | 0.00 | 0.00 | 0.00 | 0.00 | 0.01 | 0.00 | 0.00 | 0.00 | 0.00 | 0.00 |
| Labilibacter | 0.00 | 0.00 | 0.00 | 0.00 | 0.00 | 0.00 | 0.01 | 0.00 | 0.00 | 0.00 | 0.00 | 0.00 | 0.00 | 0.00 | 0.00 | 0.00 | 0.00 | 0.00 | 0.00 | 0.00 | 0.00 |
| Saccharicrinis | 0.00 | 0.00 | 0.00 | 0.00 | 0.03 | 0.00 | 0.00 | 0.00 | 0.00 | 0.00 | 0.00 | 0.00 | 0.00 | 0.00 | 0.00 | 0.00 | 0.00 | 0.00 | 0.00 | 0.00 | 0.00 |
| unclassified_Muribaculaceae | 0.09 | 0.03 | 0.33 | 0.59 | 0.18 | 0.24 | 1.26 | 0.05 | 0.02 | 0.16 | 0.04 | 0.03 | 0.07 | 0.17 | 0.04 | 0.00 | 0.00 | 0.00 | 0.00 | 0.01 | 0.00 |
| Muribaculum | 0.00 | 0.00 | 0.02 | 0.00 | 0.00 | 0.00 | 0.03 | 0.00 | 0.00 | 0.03 | 0.00 | 0.00 | 0.00 | 0.00 | 0.00 | 0.00 | 0.00 | 0.00 | 0.00 | 0.00 | 0.00 |
| mouse_gut_metagenome | 0.00 | 0.00 | 0.00 | 0.00 | 0.00 | 0.00 | 0.05 | 0.00 | 0.00 | 0.00 | 0.00 | 0.00 | 0.00 | 0.00 | 0.00 | 0.00 | 0.00 | 0.00 | 0.00 | 0.00 | 0.00 |
| unclassified_Paludibacteraceae | 0.03 | 0.00 | 0.00 | 0.10 | 0.00 | 0.07 | 0.09 | 0.05 | 0.04 | 0.00 | 0.00 | 0.00 | 0.02 | 0.00 | 0.03 | 0.00 | 0.00 | 0.00 | 0.00 | 0.00 | 0.00 |
| Porphyromonas | 0.01 | 0.04 | 0.00 | 0.00 | 0.00 | 0.00 | 0.00 | 0.00 | 0.00 | 0.00 | 0.00 | 0.00 | 0.00 | 0.06 | 0.01 | 0.00 | 0.00 | 0.00 | 0.00 | 0.00 | 0.00 |
| Alloprevotella | 0.00 | 0.08 | 0.09 | 0.00 | 0.00 | 0.13 | 0.02 | 0.00 | 0.01 | 0.03 | 0.01 | 0.01 | 0.04 | 0.03 | 0.01 | 0.00 | 0.00 | 0.00 | 0.00 | 0.00 | 0.00 |
| Prevotella | 0.06 | 0.00 | 0.00 | 0.08 | 0.00 | 0.00 | 0.00 | 0.01 | 0.00 | 0.05 | 0.00 | 0.00 | 0.00 | 0.00 | 0.00 | 0.00 | 0.00 | 0.00 | 0.00 | 0.00 | 0.00 |
| Prevotella_2 | 0.03 | 0.00 | 0.00 | 0.00 | 0.00 | 0.00 | 0.00 | 0.00 | 0.00 | 0.00 | 0.00 | 0.00 | 0.02 | 0.00 | 0.00 | 0.00 | 0.00 | 0.00 | 0.00 | 0.00 | 0.00 |
| Prevotella_6 | 0.00 | 0.00 | 0.00 | 0.24 | 0.00 | 0.00 | 0.00 | 0.00 | 0.00 | 0.00 | 0.00 | 0.00 | 0.00 | 0.00 | 0.00 | 0.00 | 0.00 | 0.00 | 0.00 | 0.00 | 0.00 |
| Prevotella_7 | 0.02 | 0.00 | 0.00 | 0.20 | 0.00 | 0.03 | 0.03 | 0.00 | 0.02 | 0.00 | 0.00 | 0.00 | 0.00 | 0.00 | 0.01 | 0.00 | 0.00 | 0.00 | 0.00 | 0.00 | 0.00 |
| Prevotella_9 | 0.04 | 0.00 | 0.00 | 0.01 | 0.01 | 0.00 | 0.00 | 0.00 | 0.01 | 0.00 | 0.01 | 0.00 | 0.01 | 0.02 | 0.01 | 0.00 | 0.00 | 0.00 | 0.01 | 0.00 | 0.00 |
| Prevotellaceae_Ga6A1_group | 0.00 | 0.00 | 0.00 | 0.00 | 0.09 | 0.00 | 0.00 | 0.00 | 0.00 | 0.00 | 0.00 | 0.00 | 0.00 | 0.09 | 0.00 | 0.00 | 0.00 | 0.00 | 0.00 | 0.00 | 0.00 |
| Prevotellaceae_UCG-001 | 0.00 | 0.00 | 0.00 | 0.00 | 0.00 | 0.00 | 0.00 | 0.00 | 0.00 | 0.03 | 0.00 | 0.00 | 0.00 | 0.00 | 0.00 | 0.00 | 0.00 | 0.00 | 0.00 | 0.00 | 0.00 |
| Mangrovibacterium | 0.00 | 0.00 | 0.00 | 0.00 | 0.00 | 0.00 | 0.00 | 0.00 | 0.00 | 0.00 | 0.00 | 0.00 | 0.00 | 0.00 | 0.00 | 0.00 | 0.00 | 0.00 | 0.01 | 0.00 | 0.00 |
| Roseimarinus | 1.82 | 0.75 | 0.38 | 0.01 | 0.01 | 0.01 | 0.01 | 0.01 | 0.02 | 0.01 | 0.01 | 0.01 | 0.01 | 0.01 | 0.02 | 0.02 | 0.04 | 0.03 | 0.58 | 0.01 | 0.02 |
| Alistipes | 0.00 | 0.01 | 0.00 | 0.00 | 0.05 | 0.04 | 0.53 | 0.00 | 0.00 | 0.05 | 0.00 | 0.00 | 0.02 | 0.10 | 0.00 | 0.00 | 0.00 | 0.00 | 0.00 | 0.00 | 0.00 |
| Rikenella | 0.00 | 0.01 | 0.00 | 0.00 | 0.00 | 0.00 | 0.00 | 0.00 | 0.00 | 0.07 | 0.00 | 0.00 | 0.00 | 0.00 | 0.00 | 0.00 | 0.00 | 0.00 | 0.00 | 0.00 | 0.00 |
| unclassified_SB-5 | 0.00 | 0.00 | 0.00 | 0.00 | 0.00 | 0.00 | 0.03 | 0.00 | 0.00 | 0.00 | 0.00 | 0.00 | 0.00 | 0.00 | 0.00 | 0.00 | 0.00 | 0.00 | 0.00 | 0.00 | 0.00 |
| Parabacteroides | 0.04 | 0.00 | 0.00 | 0.00 | 0.00 | 0.00 | 0.10 | 0.00 | 0.00 | 0.00 | 0.00 | 0.00 | 0.00 | 0.00 | 0.00 | 0.00 | 0.00 | 0.00 | 0.00 | 0.00 | 0.00 |
| unclassified_Bacteroidetes_VC2.1_Bac22 | 0.00 | 0.01 | 0.00 | 0.00 | 0.00 | 0.00 | 0.00 | 0.00 | 0.00 | 0.00 | 0.00 | 0.00 | 0.00 | 0.00 | 0.00 | 0.00 | 0.00 | 0.01 | 0.00 | 0.00 | 0.00 |
| unclassified_Chitinophagales | 0.17 | 0.03 | 0.07 | 0.01 | 0.05 | 0.02 | 0.03 | 0.05 | 0.11 | 0.00 | 0.06 | 0.03 | 0.01 | 0.03 | 0.01 | 0.01 | 0.02 | 0.02 | 0.01 | 0.01 | 0.01 |
| unclassified_37-13 | 0.00 | 0.00 | 0.00 | 0.00 | 0.00 | 0.00 | 0.00 | 0.00 | 0.00 | 0.00 | 0.00 | 0.00 | 0.00 | 0.00 | 0.00 | 0.00 | 0.00 | 0.00 | 0.00 | 0.00 | 0.00 |
| unclassified_Chitinophagaceae | 0.09 | 0.04 | 0.00 | 0.15 | 0.03 | 0.11 | 0.00 | 0.02 | 0.02 | 0.00 | 0.01 | 0.00 | 0.09 | 0.01 | 0.02 | 0.00 | 0.00 | 0.00 | 0.01 | 0.00 | 0.01 |
| Asinibacterium | 0.04 | 0.03 | 0.01 | 0.00 | 0.00 | 0.00 | 0.01 | 0.00 | 0.00 | 0.00 | 0.00 | 0.01 | 0.00 | 0.04 | 0.00 | 0.00 | 0.00 | 0.00 | 0.00 | 0.00 | 0.00 |
| Ferruginibacter | 0.00 | 0.00 | 0.00 | 0.00 | 0.00 | 0.00 | 0.00 | 0.00 | 0.00 | 0.00 | 0.00 | 0.00 | 0.00 | 0.00 | 0.00 | 0.00 | 0.00 | 0.00 | 0.00 | 0.00 | 0.00 |
| Flavihumibacter | 0.00 | 0.12 | 0.00 | 0.00 | 0.00 | 0.00 | 0.00 | 0.00 | 0.00 | 0.00 | 0.00 | 0.00 | 0.00 | 0.00 | 0.00 | 0.00 | 0.00 | 0.00 | 0.00 | 0.00 | 0.00 |
| Flavisolibacter | 0.00 | 0.00 | 0.01 | 0.00 | 0.00 | 0.00 | 0.00 | 0.00 | 0.00 | 0.00 | 0.00 | 0.00 | 0.00 | 0.00 | 0.00 | 0.00 | 0.01 | 0.01 | 0.00 | 0.00 | 0.00 |
| Flavitalea | 0.00 | 0.00 | 0.00 | 0.00 | 0.00 | 0.00 | 0.00 | 0.00 | 0.00 | 0.00 | 0.00 | 0.00 | 0.00 | 0.00 | 0.00 | 0.00 | 0.00 | 0.00 | 0.00 | 0.00 | 0.00 |
| Niabella | 0.00 | 0.00 | 0.00 | 0.04 | 0.00 | 0.00 | 0.00 | 0.00 | 0.00 | 0.00 | 0.00 | 0.00 | 0.00 | 0.00 | 0.00 | 0.00 | 0.00 | 0.00 | 0.00 | 0.00 | 0.00 |
| Sediminibacterium | 0.00 | 0.00 | 0.00 | 0.00 | 0.00 | 0.00 | 0.02 | 0.00 | 0.00 | 0.00 | 0.00 | 0.00 | 0.00 | 0.00 | 0.00 | 0.00 | 0.00 | 0.00 | 0.00 | 0.00 | 0.00 |
| Taibaiella | 0.00 | 0.04 | 0.00 | 0.00 | 0.00 | 0.00 | 0.00 | 0.02 | 0.00 | 0.00 | 0.00 | 0.00 | 0.02 | 0.00 | 0.01 | 0.00 | 0.00 | 0.00 | 0.00 | 0.00 | 0.00 |
| unclassified_Saprospiraceae | 0.25 | 0.31 | 0.25 | 0.02 | 0.14 | 0.15 | 0.09 | 0.07 | 0.07 | 0.01 | 0.09 | 0.02 | 0.04 | 0.01 | 0.07 | 0.01 | 0.04 | 0.05 | 0.03 | 0.02 | 0.02 |
| Lewinella | 0.00 | 0.00 | 0.00 | 0.00 | 0.00 | 0.00 | 0.04 | 0.01 | 0.01 | 0.00 | 0.00 | 0.01 | 0.00 | 0.00 | 0.00 | 0.00 | 0.01 | 0.00 | 0.00 | 0.00 | 0.00 |
| Bernardetia | 0.00 | 0.00 | 0.00 | 0.00 | 0.00 | 0.00 | 0.00 | 0.00 | 0.01 | 0.00 | 0.00 | 0.00 | 0.00 | 0.00 | 0.00 | 0.00 | 0.00 | 0.00 | 0.00 | 0.00 | 0.00 |
| unclassified_Cyclobacteriaceae | 0.01 | 0.00 | 0.00 | 0.00 | 0.00 | 0.00 | 0.00 | 0.02 | 0.00 | 0.00 | 0.01 | 0.01 | 0.00 | 0.01 | 0.00 | 0.00 | 0.00 | 0.00 | 0.01 | 0.00 | 0.00 |
| Algoriphagus | 0.00 | 0.00 | 0.00 | 0.00 | 0.00 | 0.00 | 0.00 | 0.00 | 0.00 | 0.00 | 0.00 | 0.00 | 0.00 | 0.00 | 0.00 | 0.00 | 0.00 | 0.00 | 0.01 | 0.00 | 0.00 |
| Cyclobacterium | 0.00 | 0.00 | 0.00 | 0.00 | 0.00 | 0.00 | 0.00 | 0.00 | 0.00 | 0.00 | 0.00 | 0.01 | 0.00 | 0.00 | 0.00 | 0.00 | 0.00 | 0.00 | 0.00 | 0.00 | 0.00 |
| Fabibacter | 0.00 | 0.00 | 0.00 | 0.00 | 0.00 | 0.00 | 0.00 | 0.00 | 0.00 | 0.00 | 0.00 | 0.00 | 0.00 | 0.00 | 0.00 | 0.00 | 0.00 | 0.02 | 0.01 | 0.00 | 0.00 |
| Reichenbachiella | 0.00 | 0.00 | 0.00 | 0.13 | 0.00 | 0.00 | 0.00 | 0.00 | 0.00 | 0.00 | 0.00 | 0.00 | 0.00 | 0.00 | 0.00 | 0.00 | 0.00 | 0.00 | 0.00 | 0.00 | 0.00 |
| Cytophaga | 0.00 | 0.00 | 0.00 | 0.00 | 0.00 | 0.00 | 0.00 | 0.00 | 0.00 | 0.00 | 0.00 | 0.00 | 0.00 | 0.00 | 0.00 | 0.00 | 0.00 | 0.00 | 0.00 | 0.00 | 0.00 |
| Flammeovirga | 0.02 | 0.11 | 0.00 | 0.00 | 0.00 | 0.00 | 0.00 | 0.00 | 0.00 | 0.00 | 0.00 | 0.00 | 0.00 | 0.00 | 0.00 | 0.00 | 0.00 | 0.01 | 0.00 | 0.00 | 0.00 |
| Hymenobacter | 0.00 | 0.00 | 0.00 | 0.00 | 0.03 | 0.01 | 0.00 | 0.00 | 0.00 | 0.00 | 0.00 | 0.00 | 0.00 | 0.00 | 0.00 | 0.00 | 0.00 | 0.00 | 0.00 | 0.00 | 0.00 |
| unclassified_Microscillaceae | 0.03 | 0.00 | 0.00 | 0.00 | 0.00 | 0.00 | 0.00 | 0.00 | 0.00 | 0.00 | 0.00 | 0.00 | 0.00 | 0.00 | 0.00 | 0.00 | 0.00 | 0.00 | 0.00 | 0.00 | 0.00 |
| Ohtaekwangia | 0.00 | 0.00 | 0.00 | 0.00 | 0.00 | 0.00 | 0.00 | 0.00 | 0.00 | 0.00 | 0.00 | 0.00 | 0.03 | 0.00 | 0.00 | 0.00 | 0.00 | 0.00 | 0.00 | 0.00 | 0.00 |
| unclassified_Spirosomaceae | 0.00 | 0.00 | 0.00 | 0.00 | 0.00 | 0.00 | 0.00 | 0.00 | 0.00 | 0.03 | 0.00 | 0.00 | 0.00 | 0.00 | 0.00 | 0.00 | 0.00 | 0.00 | 0.00 | 0.00 | 0.00 |
| Emticicia | 0.00 | 0.00 | 0.00 | 0.00 | 0.07 | 0.00 | 0.00 | 0.00 | 0.00 | 0.00 | 0.00 | 0.00 | 0.00 | 0.00 | 0.00 | 0.00 | 0.00 | 0.00 | 0.00 | 0.00 | 0.00 |
| Runella | 0.00 | 0.00 | 0.00 | 0.00 | 0.00 | 0.00 | 0.00 | 0.00 | 0.00 | 0.00 | 0.00 | 0.00 | 0.00 | 0.00 | 0.00 | 0.00 | 0.00 | 0.00 | 0.00 | 0.00 | 0.00 |
| Taeseokella | 0.02 | 0.00 | 0.00 | 0.00 | 0.00 | 0.07 | 0.00 | 0.00 | 0.00 | 0.00 | 0.00 | 0.00 | 0.02 | 0.00 | 0.00 | 0.00 | 0.00 | 0.00 | 0.00 | 0.00 | 0.00 |
| Crocinitomix | 0.09 | 0.00 | 0.01 | 0.00 | 0.00 | 0.00 | 0.00 | 0.00 | 0.00 | 0.00 | 0.02 | 0.00 | 0.00 | 0.00 | 0.00 | 0.00 | 0.00 | 0.01 | 0.00 | 0.00 | 0.00 |
| Salinirepens | 0.19 | 0.11 | 0.14 | 0.00 | 0.00 | 0.00 | 0.00 | 0.00 | 0.00 | 0.00 | 0.00 | 0.00 | 0.00 | 0.00 | 0.00 | 0.00 | 0.00 | 0.00 | 0.00 | 0.00 | 0.00 |
| unclassified_Cryomorphaceae | 0.09 | 0.22 | 0.22 | 0.22 | 0.14 | 0.32 | 0.02 | 0.05 | 0.07 | 2.81 | 3.63 | 3.61 | 0.04 | 0.02 | 0.04 | 0.06 | 28.75 | 3.30 | 0.08 | 0.04 | 0.03 |
| NS10_marine_group | 0.02 | 0.01 | 0.03 | 0.00 | 0.00 | 0.06 | 0.00 | 0.00 | 0.00 | 0.00 | 0.01 | 0.00 | 0.00 | 0.00 | 0.01 | 0.00 | 0.00 | 0.00 | 0.00 | 0.00 | 0.00 |
| Owenweeksia | 0.00 | 0.00 | 0.03 | 0.01 | 0.00 | 0.11 | 0.00 | 0.00 | 0.00 | 0.00 | 0.00 | 0.01 | 0.01 | 0.00 | 0.01 | 0.00 | 0.00 | 0.00 | 0.00 | 0.00 | 0.01 |
| Phaeocystidibacter | 0.00 | 0.00 | 0.00 | 0.00 | 0.00 | 0.00 | 0.00 | 0.00 | 0.01 | 0.00 | 0.00 | 0.00 | 0.00 | 0.00 | 0.00 | 0.00 | 0.00 | 0.00 | 0.00 | 0.00 | 0.00 |
| unclassified_Flavobacteriaceae | 0.00 | 0.01 | 0.00 | 0.00 | 0.00 | 0.00 | 0.00 | 0.01 | 0.00 | 0.00 | 0.00 | 0.00 | 0.00 | 0.00 | 0.00 | 0.00 | 0.00 | 0.00 | 0.00 | 0.00 | 0.00 |
| Algibacter | 0.05 | 0.06 | 0.01 | 0.00 | 0.00 | 0.00 | 0.00 | 0.01 | 0.02 | 0.01 | 0.01 | 0.00 | 0.00 | 0.00 | 0.01 | 0.00 | 0.00 | 0.00 | 0.00 | 0.00 | 0.00 |
| Aquimarina | 0.00 | 0.01 | 0.02 | 0.02 | 0.03 | 0.04 | 0.00 | 0.03 | 0.01 | 0.00 | 0.00 | 0.01 | 0.02 | 0.00 | 0.01 | 0.00 | 0.00 | 0.01 | 0.00 | 0.00 | 0.00 |
| Arenibacter | 0.02 | 0.00 | 0.00 | 0.00 | 0.00 | 0.00 | 0.00 | 0.00 | 0.00 | 0.00 | 0.00 | 0.00 | 0.00 | 0.00 | 0.00 | 0.00 | 0.01 | 0.01 | 0.00 | 0.00 | 0.00 |
| Aurantivirga | 1.03 | 0.25 | 0.19 | 1.05 | 0.20 | 0.21 | 0.08 | 0.34 | 0.05 | 0.14 | 0.19 | 0.05 | 0.07 | 0.05 | 0.10 | 0.00 | 0.03 | 0.05 | 0.01 | 0.02 | 0.01 |
| Corallibacter | 0.03 | 0.06 | 0.03 | 0.00 | 0.01 | 0.00 | 0.00 | 0.01 | 0.04 | 0.02 | 0.05 | 0.04 | 0.00 | 0.01 | 0.01 | 0.00 | 0.02 | 0.01 | 0.01 | 0.02 | 0.00 |
| Dokdonia | 0.01 | 0.06 | 0.00 | 0.00 | 0.00 | 0.01 | 0.01 | 0.12 | 0.02 | 0.04 | 0.06 | 0.03 | 0.01 | 0.02 | 0.01 | 0.00 | 0.00 | 0.01 | 0.00 | 0.00 | 0.00 |
| Flaviramulus | 0.11 | 0.23 | 0.04 | 0.26 | 0.22 | 0.55 | 0.01 | 0.14 | 0.08 | 0.07 | 0.20 | 0.05 | 0.09 | 0.04 | 0.03 | 0.01 | 0.02 | 0.03 | 0.02 | 0.01 | 0.01 |
| Flavirhabdus | 0.00 | 0.00 | 0.00 | 0.00 | 0.00 | 0.00 | 0.00 | 0.01 | 0.01 | 0.02 | 0.01 | 0.01 | 0.01 | 0.00 | 0.00 | 0.00 | 0.00 | 0.01 | 0.01 | 0.01 | 0.00 |
| Flavobacterium | 0.04 | 0.00 | 0.08 | 0.07 | 0.09 | 0.06 | 0.00 | 0.01 | 0.01 | 0.00 | 0.00 | 0.00 | 0.03 | 0.07 | 0.01 | 0.00 | 0.00 | 0.00 | 0.00 | 0.00 | 0.00 |
| Jejudonia | 0.15 | 0.08 | 0.06 | 0.04 | 0.03 | 0.25 | 0.01 | 0.10 | 0.15 | 0.04 | 0.33 | 0.23 | 0.05 | 0.05 | 0.04 | 0.03 | 0.04 | 0.12 | 0.05 | 0.03 | 0.02 |
| Jejuia | 0.00 | 0.00 | 0.00 | 0.00 | 0.00 | 0.02 | 0.00 | 0.00 | 0.00 | 0.00 | 0.00 | 0.00 | 0.00 | 0.00 | 0.02 | 0.00 | 0.00 | 0.00 | 0.00 | 0.00 | 0.00 |
| Kriegella | 0.00 | 0.00 | 0.00 | 0.00 | 0.00 | 0.00 | 0.00 | 0.00 | 0.00 | 0.00 | 0.00 | 0.00 | 0.00 | 0.00 | 0.00 | 0.00 | 0.00 | 0.00 | 0.00 | 0.00 | 0.00 |
| Lutibacter | 0.03 | 1.01 | 0.08 | 0.15 | 1.07 | 0.84 | 0.00 | 0.20 | 0.00 | 0.00 | 0.06 | 0.02 | 0.07 | 0.00 | 0.02 | 0.00 | 0.00 | 0.02 | 0.02 | 0.01 | 0.00 |
| Lutimonas | 0.04 | 0.02 | 0.01 | 0.00 | 0.00 | 0.09 | 0.00 | 0.01 | 0.00 | 0.00 | 0.05 | 0.00 | 0.00 | 0.00 | 0.00 | 0.00 | 0.00 | 0.01 | 0.00 | 0.00 | 0.00 |
| Maribacter | 0.04 | 0.14 | 0.11 | 0.01 | 0.02 | 0.21 | 0.01 | 0.10 | 0.07 | 0.00 | 0.04 | 0.02 | 0.01 | 0.01 | 0.04 | 0.02 | 0.01 | 0.01 | 0.02 | 0.01 | 0.02 |
| Maritimimonas | 0.02 | 0.01 | 0.03 | 0.00 | 0.06 | 0.02 | 0.00 | 0.02 | 0.00 | 0.00 | 0.00 | 0.00 | 0.00 | 0.00 | 0.00 | 0.00 | 0.00 | 0.00 | 0.00 | 0.00 | 0.00 |
| Marixanthomonas | 0.00 | 0.00 | 0.00 | 0.00 | 0.00 | 0.00 | 0.00 | 0.00 | 0.01 | 0.00 | 0.00 | 0.00 | 0.00 | 0.00 | 0.00 | 0.00 | 0.00 | 0.00 | 0.00 | 0.00 | 0.00 |
| Mesonia | 0.00 | 0.00 | 0.00 | 0.00 | 0.00 | 0.00 | 0.00 | 0.01 | 0.00 | 0.00 | 0.00 | 0.00 | 0.01 | 0.02 | 0.02 | 0.00 | 0.00 | 0.00 | 0.00 | 0.00 | 0.00 |
| Muricauda | 0.01 | 0.00 | 0.00 | 0.00 | 0.00 | 0.02 | 0.00 | 0.01 | 0.01 | 0.00 | 0.02 | 0.00 | 0.01 | 0.00 | 0.00 | 0.00 | 0.00 | 0.00 | 0.01 | 0.00 | 0.00 |
| Myroides | 0.00 | 0.00 | 0.00 | 0.01 | 0.00 | 0.00 | 0.00 | 0.00 | 0.00 | 0.00 | 0.00 | 0.00 | 0.00 | 0.00 | 0.00 | 0.00 | 0.00 | 0.00 | 0.00 | 0.00 | 0.00 |
| NS3a_marine_group | 0.17 | 0.04 | 0.02 | 0.00 | 0.00 | 0.00 | 0.00 | 0.00 | 0.00 | 0.00 | 0.00 | 0.00 | 0.00 | 0.00 | 0.00 | 0.00 | 0.00 | 0.00 | 0.00 | 0.00 | 0.00 |
| Nonlabens | 0.01 | 0.00 | 0.00 | 0.00 | 0.00 | 0.00 | 0.00 | 0.00 | 0.00 | 0.00 | 0.00 | 0.00 | 0.00 | 0.00 | 0.00 | 0.00 | 0.00 | 0.00 | 0.00 | 0.00 | 0.00 |
| Olleya | 0.00 | 0.00 | 0.00 | 0.00 | 0.00 | 0.00 | 0.00 | 0.01 | 0.00 | 0.00 | 0.00 | 0.00 | 0.00 | 0.00 | 0.00 | 0.00 | 0.00 | 0.00 | 0.00 | 0.00 | 0.01 |
| Polaribacter_1 | 0.64 | 0.59 | 0.59 | 0.28 | 0.32 | 0.36 | 0.53 | 1.87 | 3.82 | 0.57 | 0.71 | 0.70 | 1.21 | 0.62 | 1.16 | 0.27 | 0.54 | 0.63 | 0.76 | 0.84 | 0.35 |
| Polaribacter_4 | 4.38 | 4.84 | 3.77 | 0.45 | 0.14 | 0.50 | 2.24 | 13.78 | 4.16 | 0.23 | 0.24 | 0.26 | 2.88 | 0.61 | 2.25 | 0.07 | 0.14 | 0.18 | 0.24 | 0.17 | 0.09 |
| Pseudofulvibacter | 0.00 | 0.00 | 0.00 | 0.00 | 0.00 | 0.00 | 0.00 | 0.00 | 0.00 | 0.00 | 0.00 | 0.00 | 0.01 | 0.00 | 0.01 | 0.00 | 0.00 | 0.00 | 0.00 | 0.00 | 0.00 |
| Spongiivirga | 0.02 | 0.00 | 0.00 | 0.00 | 0.00 | 0.00 | 0.00 | 0.00 | 0.00 | 0.00 | 0.00 | 0.00 | 0.00 | 0.00 | 0.00 | 0.00 | 0.00 | 0.00 | 0.00 | 0.00 | 0.00 |
| Tamlana | 0.00 | 0.00 | 0.00 | 0.00 | 0.00 | 0.00 | 0.00 | 0.00 | 0.00 | 0.00 | 0.00 | 0.01 | 0.00 | 0.00 | 0.00 | 0.00 | 0.00 | 0.00 | 0.01 | 0.00 | 0.00 |
| Tenacibaculum | 6.68 | 2.72 | 3.45 | 2.17 | 1.83 | 2.70 | 1.09 | 0.93 | 0.69 | 1.95 | 2.93 | 3.45 | 1.74 | 0.56 | 1.88 | 0.10 | 0.88 | 1.07 | 0.99 | 0.38 | 0.42 |
| Ulvibacter | 0.00 | 0.00 | 0.00 | 0.00 | 0.00 | 0.00 | 0.00 | 0.01 | 0.00 | 0.00 | 0.02 | 0.00 | 0.00 | 0.00 | 0.00 | 0.00 | 0.00 | 0.00 | 0.00 | 0.00 | 0.00 |
| Winogradskyella | 0.00 | 0.01 | 0.02 | 0.00 | 0.00 | 0.00 | 0.00 | 0.03 | 0.01 | 0.00 | 0.00 | 0.01 | 0.00 | 0.01 | 0.01 | 0.01 | 0.00 | 0.01 | 0.01 | 0.00 | 0.00 |
| Polaribacter huanghezhanensis | 0.16 | 0.09 | 0.19 | 0.13 | 0.09 | 0.22 | 0.05 | 0.32 | 0.20 | 3.45 | 8.96 | 5.47 | 0.17 | 0.08 | 0.21 | 0.29 | 8.21 | 10.56 | 1.15 | 0.63 | 1.43 |
| unclassified_NS9_marine_group | 0.04 | 0.00 | 0.00 | 0.00 | 0.00 | 0.00 | 0.00 | 0.01 | 0.00 | 0.00 | 0.00 | 0.00 | 0.00 | 0.00 | 0.00 | 0.00 | 0.01 | 0.03 | 0.01 | 0.00 | 0.00 |
| unclassified_Weeksellaceae | 0.00 | 0.00 | 0.00 | 0.01 | 0.00 | 0.00 | 0.00 | 0.00 | 0.00 | 0.00 | 0.00 | 0.00 | 0.00 | 0.00 | 0.00 | 0.00 | 0.00 | 0.00 | 0.00 | 0.00 | 0.00 |
| Chryseobacterium | 0.23 | 0.58 | 0.63 | 0.96 | 0.97 | 0.61 | 0.45 | 0.11 | 0.06 | 0.33 | 0.08 | 0.10 | 0.24 | 0.46 | 0.11 | 0.01 | 0.01 | 0.01 | 0.01 | 0.01 | 0.01 |
| Cloacibacterium | 0.00 | 0.00 | 0.00 | 0.00 | 0.00 | 0.00 | 0.00 | 0.00 | 0.00 | 0.00 | 0.00 | 0.00 | 0.01 | 0.00 | 0.00 | 0.00 | 0.00 | 0.00 | 0.00 | 0.00 | 0.00 |
| Elizabethkingia | 0.00 | 0.00 | 0.00 | 0.00 | 0.03 | 0.00 | 0.00 | 0.00 | 0.00 | 0.01 | 0.00 | 0.00 | 0.00 | 0.00 | 0.00 | 0.00 | 0.00 | 0.00 | 0.00 | 0.00 | 0.00 |
| Moheibacter | 0.01 | 0.00 | 0.00 | 0.00 | 0.00 | 0.00 | 0.02 | 0.00 | 0.00 | 0.00 | 0.00 | 0.00 | 0.00 | 0.00 | 0.00 | 0.00 | 0.00 | 0.00 | 0.00 | 0.00 | 0.00 |
| unclassified_Lentimicrobiaceae | 0.00 | 0.04 | 0.00 | 0.00 | 0.11 | 0.00 | 0.00 | 0.00 | 0.00 | 0.00 | 0.00 | 0.01 | 0.00 | 0.00 | 0.00 | 0.00 | 0.00 | 0.00 | 0.00 | 0.00 | 0.00 |
| unclassified_NS11-12_marine_group | 0.00 | 0.00 | 0.00 | 0.00 | 0.00 | 0.00 | 0.00 | 0.01 | 0.00 | 0.07 | 0.01 | 0.00 | 0.02 | 0.00 | 0.00 | 0.00 | 0.01 | 0.00 | 0.00 | 0.00 | 0.00 |
| Mucilaginibacter | 0.00 | 0.00 | 0.00 | 0.00 | 0.00 | 0.00 | 0.05 | 0.01 | 0.00 | 0.00 | 0.00 | 0.00 | 0.00 | 0.00 | 0.00 | 0.00 | 0.00 | 0.00 | 0.00 | 0.00 | 0.00 |
| Nubsella | 0.12 | 0.19 | 0.10 | 0.34 | 0.27 | 0.29 | 0.14 | 0.02 | 0.00 | 0.08 | 0.02 | 0.01 | 0.00 | 0.06 | 0.02 | 0.00 | 0.00 | 0.01 | 0.02 | 0.00 | 0.00 |
| Pedobacter | 0.01 | 0.00 | 0.00 | 0.00 | 0.00 | 0.00 | 0.01 | 0.00 | 0.00 | 0.00 | 0.00 | 0.00 | 0.00 | 0.01 | 0.01 | 0.00 | 0.00 | 0.00 | 0.01 | 0.00 | 0.00 |
| Sphingobacterium | 0.00 | 0.00 | 0.00 | 0.00 | 0.00 | 0.00 | 0.00 | 0.00 | 0.00 | 0.00 | 0.00 | 0.00 | 0.00 | 0.00 | 0.01 | 0.00 | 0.00 | 0.00 | 0.00 | 0.00 | 0.00 |
| unclassified_env.OPS_17 | 0.00 | 0.00 | 0.00 | 0.00 | 0.15 | 0.00 | 0.00 | 0.00 | 0.00 | 0.00 | 0.00 | 0.00 | 0.00 | 0.00 | 0.00 | 0.00 | 0.00 | 0.00 | 0.00 | 0.00 | 0.00 |
| Ignavibacterium | 0.00 | 0.00 | 0.00 | 0.00 | 0.00 | 0.00 | 0.03 | 0.00 | 0.00 | 0.00 | 0.00 | 0.00 | 0.00 | 0.00 | 0.00 | 0.00 | 0.00 | 0.00 | 0.00 | 0.00 | 0.00 |
| IheB3-7 | 0.00 | 0.00 | 0.00 | 0.00 | 0.06 | 0.00 | 0.03 | 0.00 | 0.00 | 0.00 | 0.03 | 0.02 | 0.00 | 0.00 | 0.00 | 0.00 | 0.00 | 0.00 | 0.00 | 0.00 | 0.00 |
| unclassified_PHOS-HE36 | 0.00 | 0.00 | 0.00 | 0.00 | 0.00 | 0.00 | 0.01 | 0.00 | 0.00 | 0.00 | 0.00 | 0.00 | 0.00 | 0.00 | 0.00 | 0.00 | 0.00 | 0.00 | 0.00 | 0.00 | 0.00 |
| unclassified_OPB56 | 0.03 | 0.00 | 0.08 | 0.00 | 0.00 | 0.00 | 0.00 | 0.06 | 0.15 | 0.02 | 0.10 | 0.09 | 0.10 | 0.00 | 0.07 | 0.00 | 0.00 | 0.04 | 0.01 | 0.02 | 0.00 |
| unclassified_SJA-28 | 0.00 | 0.00 | 0.00 | 0.00 | 0.23 | 0.00 | 0.00 | 0.00 | 0.00 | 0.00 | 0.00 | 0.00 | 0.00 | 0.00 | 0.00 | 0.00 | 0.00 | 0.00 | 0.00 | 0.00 | 0.00 |
| Balneola | 0.00 | 0.00 | 0.00 | 0.00 | 0.00 | 0.00 | 0.00 | 0.01 | 0.00 | 0.00 | 0.00 | 0.00 | 0.00 | 0.00 | 0.02 | 0.00 | 0.00 | 0.00 | 0.00 | 0.00 | 0.00 |
| Gracilimonas | 0.01 | 0.00 | 0.00 | 0.00 | 0.00 | 0.00 | 0.00 | 0.00 | 0.00 | 0.00 | 0.00 | 0.00 | 0.00 | 0.00 | 0.00 | 0.00 | 0.00 | 0.00 | 0.00 | 0.00 | 0.00 |
| Rubrivirga | 0.00 | 0.00 | 0.01 | 0.00 | 0.00 | 0.00 | 0.00 | 0.02 | 0.01 | 0.00 | 0.00 | 0.00 | 0.00 | 0.00 | 0.01 | 0.00 | 0.00 | 0.00 | 0.00 | 0.00 | 0.00 |
| Caldisericum | 0.01 | 0.00 | 0.00 | 0.01 | 0.00 | 0.01 | 0.00 | 0.01 | 0.00 | 0.00 | 0.00 | 0.01 | 0.00 | 0.01 | 0.01 | 0.00 | 0.00 | 0.00 | 0.00 | 0.01 | 0.00 |
| unclassified_LF045 | 0.00 | 0.00 | 0.00 | 0.00 | 0.00 | 0.00 | 0.00 | 0.00 | 0.00 | 0.00 | 0.00 | 0.00 | 0.00 | 0.00 | 0.00 | 0.00 | 0.00 | 0.00 | 0.00 | 0.00 | 0.00 |
| unclassified_WCHB1-02 | 0.00 | 0.00 | 0.00 | 0.00 | 0.00 | 0.00 | 0.00 | 0.00 | 0.00 | 0.00 | 0.00 | 0.00 | 0.00 | 0.00 | 0.00 | 0.00 | 0.00 | 0.00 | 0.00 | 0.00 | 0.00 |
| unclassified_Chlamydiaceae | 0.27 | 0.29 | 0.18 | 0.14 | 0.03 | 0.49 | 0.07 | 0.05 | 0.02 | 0.09 | 0.04 | 0.05 | 0.23 | 0.14 | 0.48 | 0.01 | 0.01 | 0.03 | 0.03 | 0.03 | 0.01 |
| unclassified_Criblamydiaceae | 0.00 | 0.00 | 0.00 | 0.01 | 0.00 | 0.00 | 0.00 | 0.00 | 0.00 | 0.00 | 0.00 | 0.00 | 0.00 | 0.00 | 0.00 | 0.00 | 0.00 | 0.00 | 0.00 | 0.00 | 0.00 |
| unclassified_Parachlamydiaceae | 0.00 | 0.00 | 0.00 | 0.00 | 0.00 | 0.00 | 0.00 | 0.00 | 0.00 | 0.00 | 0.00 | 0.00 | 0.00 | 0.00 | 0.03 | 0.00 | 0.00 | 0.00 | 0.00 | 0.00 | 0.00 |
| Neochlamydia | 0.00 | 0.00 | 0.00 | 0.00 | 0.00 | 0.00 | 0.00 | 0.00 | 0.00 | 0.00 | 0.00 | 0.02 | 0.00 | 0.00 | 0.01 | 0.00 | 0.00 | 0.00 | 0.00 | 0.00 | 0.00 |
| unclassified_Simkaniaceae | 0.00 | 0.00 | 0.00 | 0.00 | 0.00 | 0.00 | 0.00 | 0.00 | 0.00 | 0.00 | 0.01 | 0.00 | 0.00 | 0.00 | 0.00 | 0.00 | 0.00 | 0.00 | 0.00 | 0.00 | 0.00 |
| Candidatus_Fritschea | 0.07 | 0.04 | 0.00 | 0.05 | 0.00 | 0.00 | 0.00 | 0.01 | 0.01 | 0.00 | 0.00 | 0.00 | 0.00 | 0.01 | 0.01 | 0.00 | 0.00 | 0.00 | 0.03 | 0.01 | 0.00 |
| unclassified_1-20 | 0.00 | 0.00 | 0.00 | 0.00 | 0.00 | 0.00 | 0.01 | 0.00 | 0.00 | 0.00 | 0.00 | 0.00 | 0.00 | 0.00 | 0.00 | 0.00 | 0.00 | 0.00 | 0.00 | 0.00 | 0.00 |
| unclassified_Anaerolineaceae | 0.03 | 0.01 | 0.00 | 0.80 | 0.01 | 0.00 | 0.15 | 0.00 | 0.00 | 0.03 | 0.00 | 0.00 | 0.00 | 0.01 | 0.01 | 0.00 | 0.01 | 0.01 | 0.01 | 0.00 | 0.00 |
| Anaerolinea | 0.00 | 0.00 | 0.00 | 0.00 | 0.00 | 0.00 | 0.05 | 0.00 | 0.00 | 0.00 | 0.00 | 0.00 | 0.00 | 0.00 | 0.00 | 0.00 | 0.00 | 0.00 | 0.00 | 0.00 | 0.00 |
| Bellilinea | 0.00 | 0.00 | 0.00 | 0.00 | 0.00 | 0.00 | 0.00 | 0.00 | 0.00 | 0.00 | 0.00 | 0.00 | 0.00 | 0.00 | 0.00 | 0.00 | 0.00 | 0.00 | 0.00 | 0.00 | 0.00 |
| Leptolinea | 0.00 | 0.00 | 0.00 | 0.00 | 0.00 | 0.00 | 0.00 | 0.00 | 0.00 | 0.00 | 0.00 | 0.01 | 0.00 | 0.00 | 0.01 | 0.01 | 0.01 | 0.00 | 0.00 | 0.00 | 0.00 |
| Longilinea | 0.00 | 0.00 | 0.00 | 0.14 | 0.00 | 0.00 | 0.05 | 0.00 | 0.00 | 0.00 | 0.00 | 0.00 | 0.00 | 0.00 | 0.00 | 0.00 | 0.00 | 0.00 | 0.00 | 0.00 | 0.00 |
| unclassified_Ardenticatenales | 0.00 | 0.00 | 0.00 | 0.00 | 0.00 | 0.00 | 0.03 | 0.00 | 0.00 | 0.00 | 0.00 | 0.00 | 0.00 | 0.00 | 0.00 | 0.00 | 0.00 | 0.00 | 0.00 | 0.00 | 0.00 |
| unclassified_Caldilineaceae | 0.00 | 0.00 | 0.00 | 0.00 | 0.00 | 0.00 | 0.01 | 0.00 | 0.00 | 0.00 | 0.00 | 0.00 | 0.00 | 0.00 | 0.00 | 0.00 | 0.00 | 0.00 | 0.00 | 0.00 | 0.00 |
| unclassified_RBG-13-54-9 | 0.01 | 0.00 | 0.00 | 0.00 | 0.10 | 0.01 | 0.00 | 0.00 | 0.00 | 0.00 | 0.00 | 0.00 | 0.00 | 0.01 | 0.00 | 0.00 | 0.00 | 0.01 | 0.00 | 0.00 | 0.00 |
| unclassified_SBR1031 | 0.00 | 0.00 | 0.00 | 0.00 | 0.00 | 0.00 | 0.02 | 0.00 | 0.00 | 0.00 | 0.00 | 0.00 | 0.00 | 0.00 | 0.00 | 0.00 | 0.00 | 0.00 | 0.00 | 0.00 | 0.00 |
| unclassified_A4b | 0.00 | 0.00 | 0.00 | 0.01 | 0.00 | 0.28 | 0.06 | 0.00 | 0.00 | 0.00 | 0.02 | 0.02 | 0.00 | 0.00 | 0.00 | 0.01 | 0.00 | 0.01 | 0.01 | 0.00 | 0.00 |
| anaerobic_bacterium_MO-CFX2 | 0.00 | 0.00 | 0.00 | 0.00 | 0.00 | 0.00 | 0.12 | 0.00 | 0.00 | 0.00 | 0.00 | 0.00 | 0.00 | 0.00 | 0.00 | 0.00 | 0.00 | 0.00 | 0.00 | 0.00 | 0.00 |
| wastewater_metagenome | 0.00 | 0.00 | 0.00 | 0.13 | 0.00 | 0.00 | 0.00 | 0.00 | 0.00 | 0.00 | 0.00 | 0.00 | 0.00 | 0.00 | 0.00 | 0.00 | 0.00 | 0.00 | 0.00 | 0.00 | 0.00 |
| unclassified_SJA-15 | 0.00 | 0.00 | 0.00 | 0.24 | 0.00 | 0.00 | 0.28 | 0.00 | 0.00 | 0.00 | 0.00 | 0.00 | 0.00 | 0.00 | 0.00 | 0.00 | 0.00 | 0.00 | 0.00 | 0.00 | 0.00 |
| unclassified_MSBL5 | 0.00 | 0.00 | 0.01 | 0.00 | 0.00 | 0.00 | 0.00 | 0.00 | 0.00 | 0.00 | 0.00 | 0.00 | 0.00 | 0.00 | 0.00 | 0.00 | 0.00 | 0.00 | 0.00 | 0.00 | 0.00 |
| unclassified_JG30-KF-CM66 | 0.00 | 0.00 | 0.00 | 0.17 | 0.07 | 0.00 | 0.00 | 0.00 | 0.00 | 0.00 | 0.00 | 0.00 | 0.05 | 0.00 | 0.00 | 0.00 | 0.00 | 0.00 | 0.00 | 0.00 | 0.00 |
| unclassified_KD4-96 | 0.00 | 0.00 | 0.01 | 0.57 | 0.00 | 0.00 | 0.00 | 0.00 | 0.00 | 0.00 | 0.01 | 0.00 | 0.00 | 0.00 | 0.00 | 0.00 | 0.00 | 0.00 | 0.00 | 0.00 | 0.00 |
| unclassified_JG30-KF-AS9 | 0.00 | 0.00 | 0.00 | 0.00 | 0.00 | 0.00 | 0.00 | 0.00 | 0.00 | 0.00 | 0.00 | 0.00 | 0.00 | 0.00 | 0.01 | 0.00 | 0.00 | 0.00 | 0.00 | 0.00 | 0.00 |
| unclassified_Ktedonobacteraceae | 0.00 | 0.00 | 0.00 | 0.00 | 0.00 | 0.00 | 0.00 | 0.00 | 0.00 | 0.00 | 0.00 | 0.00 | 0.00 | 0.00 | 0.00 | 0.00 | 0.00 | 0.00 | 0.00 | 0.00 | 0.00 |
| BacC-u-018 | 0.00 | 0.00 | 0.00 | 0.00 | 0.00 | 0.00 | 0.00 | 0.00 | 0.00 | 0.00 | 0.00 | 0.00 | 0.00 | 0.00 | 0.00 | 0.00 | 0.00 | 0.00 | 0.00 | 0.00 | 0.00 |
| Ktedonobacter | 0.00 | 0.00 | 0.00 | 0.28 | 0.00 | 0.00 | 0.00 | 0.00 | 0.00 | 0.00 | 0.00 | 0.00 | 0.00 | 0.00 | 0.00 | 0.00 | 0.00 | 0.00 | 0.00 | 0.00 | 0.00 |
| unclassified_TK10 | 0.00 | 0.00 | 0.00 | 0.00 | 0.00 | 0.00 | 0.00 | 0.00 | 0.00 | 0.00 | 0.00 | 0.00 | 0.00 | 0.00 | 0.00 | 0.00 | 0.00 | 0.00 | 0.00 | 0.00 | 0.00 |
| unclassified_Caenarcaniphilales | 0.03 | 0.00 | 0.00 | 0.00 | 0.00 | 0.00 | 0.00 | 0.01 | 0.08 | 0.00 | 0.03 | 0.06 | 0.02 | 0.00 | 0.02 | 0.00 | 0.00 | 0.01 | 0.00 | 0.00 | 0.00 |
| unclassified_Gastranaerophilales | 0.00 | 0.00 | 0.00 | 0.00 | 0.00 | 0.00 | 0.06 | 0.00 | 0.00 | 0.00 | 0.00 | 0.00 | 0.00 | 0.00 | 0.00 | 0.00 | 0.00 | 0.00 | 0.00 | 0.00 | 0.00 |
| Candidatus_Melainabacteria_bacterium_RIFOXYA2_FULL_32_9 | 0.00 | 0.00 | 0.00 | 0.45 | 0.00 | 0.00 | 0.00 | 0.00 | 0.00 | 0.00 | 0.00 | 0.00 | 0.00 | 0.00 | 0.00 | 0.00 | 0.00 | 0.00 | 0.00 | 0.00 | 0.00 |
| unclassified_Obscuribacterales | 0.00 | 0.00 | 0.00 | 0.00 | 0.00 | 0.00 | 0.00 | 0.00 | 0.00 | 0.00 | 0.01 | 0.00 | 0.00 | 0.00 | 0.00 | 0.00 | 0.00 | 0.00 | 0.00 | 0.00 | 0.00 |
| unclassified_Chroococcidiopsaceae | 0.00 | 0.00 | 0.00 | 0.00 | 0.00 | 0.00 | 0.00 | 0.00 | 0.00 | 0.00 | 0.00 | 0.00 | 0.00 | 0.04 | 0.00 | 0.00 | 0.00 | 0.00 | 0.00 | 0.00 | 0.00 |
| Aliterella_CENA595 | 0.00 | 0.00 | 0.00 | 0.00 | 0.00 | 0.00 | 0.00 | 0.00 | 0.00 | 0.00 | 0.00 | 0.00 | 0.01 | 0.00 | 0.00 | 0.00 | 0.00 | 0.00 | 0.00 | 0.00 | 0.00 |
| Chroococcidiopsis_PCC_7203 | 0.00 | 0.00 | 0.00 | 0.00 | 0.00 | 0.00 | 0.07 | 0.00 | 0.00 | 0.00 | 0.00 | 0.00 | 0.00 | 0.00 | 0.00 | 0.00 | 0.00 | 0.00 | 0.00 | 0.00 | 0.00 |
| unclassified_Nostocaceae | 0.00 | 0.00 | 0.00 | 0.00 | 0.00 | 0.00 | 0.03 | 0.00 | 0.00 | 0.00 | 0.00 | 0.00 | 0.00 | 0.00 | 0.00 | 0.00 | 0.00 | 0.00 | 0.00 | 0.00 | 0.00 |
| Nodularia_PCC-9350 | 0.00 | 0.00 | 0.00 | 0.13 | 0.00 | 0.00 | 0.00 | 0.00 | 0.00 | 0.00 | 0.00 | 0.00 | 0.00 | 0.00 | 0.00 | 0.00 | 0.00 | 0.00 | 0.00 | 0.00 | 0.00 |
| Planktothrix_NIVA-CYA_15 | 0.01 | 0.00 | 0.00 | 0.00 | 0.00 | 0.00 | 0.01 | 0.00 | 0.00 | 0.00 | 0.00 | 0.00 | 0.00 | 0.00 | 0.00 | 0.00 | 0.00 | 0.00 | 0.00 | 0.00 | 0.00 |
| Cyanobium_PCC-6307 | 0.00 | 0.00 | 0.00 | 0.00 | 0.00 | 0.00 | 0.05 | 0.00 | 0.00 | 0.00 | 0.00 | 0.00 | 0.00 | 0.00 | 0.00 | 0.00 | 0.00 | 0.00 | 0.00 | 0.00 | 0.00 |
| unclassified_Sericytochromatia | 0.02 | 0.00 | 0.00 | 0.27 | 0.00 | 0.00 | 0.00 | 0.00 | 0.00 | 0.00 | 0.00 | 0.00 | 0.00 | 0.00 | 0.00 | 0.00 | 0.00 | 0.00 | 0.00 | 0.00 | 0.00 |
| Hyaloperonospora_arabidopsidis | 0.23 | 0.11 | 0.06 | 0.01 | 0.00 | 0.00 | 0.07 | 0.02 | 0.10 | 0.00 | 0.00 | 0.01 | 0.01 | 0.04 | 0.02 | 0.01 | 0.00 | 0.00 | 0.00 | 0.01 | 0.00 |
| Deinococcus | 0.00 | 0.05 | 0.05 | 0.00 | 0.00 | 0.09 | 0.00 | 0.00 | 0.00 | 0.00 | 0.00 | 0.00 | 0.00 | 0.00 | 0.00 | 0.00 | 0.00 | 0.00 | 0.00 | 0.00 | 0.00 |
| Thermus | 0.00 | 0.00 | 0.00 | 0.00 | 0.00 | 0.00 | 0.00 | 0.00 | 0.00 | 0.00 | 0.00 | 0.00 | 0.00 | 0.00 | 0.05 | 0.00 | 0.00 | 0.00 | 0.00 | 0.00 | 0.00 |
| unclassified_Babeliales | 0.10 | 0.01 | 0.05 | 0.00 | 0.02 | 0.09 | 0.01 | 0.00 | 0.00 | 0.04 | 0.00 | 0.00 | 0.04 | 0.03 | 0.13 | 0.00 | 0.01 | 0.00 | 0.00 | 0.00 | 0.01 |
| unclassified_UBA12409 | 0.00 | 0.00 | 0.00 | 0.00 | 0.00 | 0.00 | 0.00 | 0.00 | 0.00 | 0.00 | 0.00 | 0.00 | 0.00 | 0.00 | 0.01 | 0.00 | 0.00 | 0.00 | 0.00 | 0.00 | 0.00 |
| unclassified_Vermiphilaceae | 0.01 | 0.00 | 0.00 | 0.00 | 0.10 | 0.09 | 0.00 | 0.01 | 0.02 | 0.08 | 0.03 | 0.08 | 0.00 | 0.00 | 0.05 | 0.00 | 0.00 | 0.01 | 0.04 | 0.00 | 0.00 |
| unclassified_Lineage_IIb | 0.00 | 0.00 | 0.00 | 0.00 | 0.00 | 0.00 | 0.00 | 0.00 | 0.00 | 0.00 | 0.00 | 0.00 | 0.00 | 0.00 | 0.00 | 0.00 | 0.00 | 0.00 | 0.00 | 0.00 | 0.00 |
| Candidatus_Entotheonella | 0.00 | 0.00 | 0.00 | 0.00 | 0.00 | 0.00 | 0.03 | 0.00 | 0.00 | 0.00 | 0.00 | 0.00 | 0.00 | 0.00 | 0.00 | 0.00 | 0.00 | 0.00 | 0.00 | 0.00 | 0.00 |
| Arcobacter | 14.81 | 8.03 | 11.51 | 0.54 | 1.31 | 0.91 | 5.61 | 0.80 | 3.82 | 1.65 | 17.06 | 13.49 | 14.61 | 22.15 | 15.37 | 1.45 | 10.43 | 10.70 | 8.82 | 10.57 | 13.40 |
| Helicobacter | 0.00 | 0.00 | 0.00 | 0.00 | 0.00 | 0.00 | 0.33 | 0.00 | 0.00 | 0.00 | 0.00 | 0.00 | 0.04 | 0.00 | 0.00 | 0.00 | 0.00 | 0.00 | 0.00 | 0.00 | 0.00 |
| Sulfurospirillum | 0.00 | 0.00 | 0.00 | 0.00 | 0.00 | 0.00 | 0.00 | 0.00 | 0.00 | 0.06 | 0.00 | 0.00 | 0.00 | 0.00 | 0.00 | 0.00 | 0.00 | 0.00 | 0.00 | 0.00 | 0.00 |
| Sulfurovum | 0.01 | 0.01 | 0.01 | 0.01 | 0.01 | 0.02 | 0.01 | 0.01 | 0.01 | 0.01 | 0.01 | 0.01 | 0.01 | 0.01 | 0.00 | 0.00 | 0.00 | 0.00 | 0.01 | 0.01 | 0.01 |
| Sulfuricurvum | 0.01 | 0.00 | 0.01 | 0.00 | 0.00 | 0.02 | 0.00 | 0.01 | 0.00 | 0.01 | 0.00 | 0.01 | 0.00 | 0.01 | 0.01 | 0.01 | 0.00 | 0.01 | 0.00 | 0.01 | 0.01 |
| Sulfurimonas | 0.00 | 0.00 | 0.00 | 0.02 | 0.02 | 0.00 | 0.03 | 0.00 | 0.00 | 0.00 | 0.00 | 0.00 | 0.00 | 0.00 | 0.00 | 0.02 | 0.00 | 0.00 | 0.01 | 0.00 | 0.00 |
| possible_genus_03 | 0.00 | 0.00 | 0.00 | 0.00 | 0.04 | 0.00 | 0.00 | 0.00 | 0.00 | 0.00 | 0.00 | 0.00 | 0.00 | 0.00 | 0.00 | 0.00 | 0.00 | 0.00 | 0.00 | 0.00 | 0.00 |
| unclassified_B122 | 0.00 | 0.00 | 0.00 | 0.25 | 0.00 | 0.00 | 0.00 | 0.00 | 0.00 | 0.00 | 0.00 | 0.00 | 0.00 | 0.00 | 0.00 | 0.00 | 0.00 | 0.00 | 0.00 | 0.00 | 0.00 |
| unclassified_Fibrobacteraceae | 0.01 | 0.00 | 0.05 | 0.00 | 0.00 | 0.00 | 0.00 | 0.00 | 0.00 | 0.00 | 0.00 | 0.04 | 0.00 | 0.00 | 0.01 | 0.00 | 0.00 | 0.00 | 0.00 | 0.00 | 0.00 |
| Aeribacillus | 0.02 | 0.00 | 0.00 | 0.00 | 0.00 | 0.00 | 0.03 | 0.00 | 0.00 | 0.00 | 0.00 | 0.00 | 0.00 | 0.00 | 0.00 | 0.00 | 0.00 | 0.00 | 0.00 | 0.00 | 0.00 |
| Anoxybacillus | 0.03 | 0.02 | 0.02 | 0.28 | 0.00 | 0.00 | 0.03 | 0.00 | 0.01 | 0.00 | 0.00 | 0.00 | 0.90 | 0.14 | 0.04 | 0.01 | 0.00 | 0.01 | 0.00 | 0.00 | 0.00 |
| Bacillus | 0.93 | 1.82 | 3.27 | 4.91 | 8.68 | 3.88 | 3.52 | 0.58 | 0.19 | 1.77 | 0.37 | 0.27 | 0.09 | 0.40 | 0.07 | 0.03 | 0.04 | 0.04 | 0.05 | 0.07 | 0.02 |
| Geobacillus | 0.00 | 0.00 | 0.00 | 0.00 | 0.00 | 0.00 | 0.00 | 0.00 | 0.00 | 0.05 | 0.00 | 0.00 | 0.03 | 0.05 | 0.01 | 0.00 | 0.00 | 0.00 | 0.00 | 0.00 | 0.00 |
| Halobacillus | 0.00 | 0.00 | 0.00 | 0.00 | 0.11 | 0.00 | 0.00 | 0.00 | 0.00 | 0.00 | 0.00 | 0.00 | 0.00 | 0.00 | 0.00 | 0.00 | 0.00 | 0.00 | 0.00 | 0.00 | 0.00 |
| Tepidibacillus | 0.00 | 0.00 | 0.00 | 0.15 | 0.00 | 0.00 | 0.00 | 0.00 | 0.00 | 0.00 | 0.00 | 0.00 | 0.00 | 0.00 | 0.00 | 0.00 | 0.00 | 0.00 | 0.00 | 0.00 | 0.00 |
| Thermicanus | 0.00 | 0.00 | 0.00 | 0.00 | 0.00 | 0.00 | 0.00 | 0.00 | 0.00 | 0.00 | 0.00 | 0.00 | 0.00 | 0.02 | 0.00 | 0.00 | 0.00 | 0.00 | 0.00 | 0.00 | 0.00 |
| Gemella | 0.00 | 0.00 | 0.00 | 0.00 | 0.00 | 0.02 | 0.03 | 0.00 | 0.01 | 0.00 | 0.00 | 0.01 | 0.02 | 0.06 | 0.00 | 0.00 | 0.00 | 0.00 | 0.00 | 0.00 | 0.00 |
| Exiguobacterium | 0.00 | 0.06 | 0.06 | 0.02 | 0.12 | 0.05 | 0.05 | 0.01 | 0.01 | 0.00 | 0.00 | 0.02 | 0.03 | 0.00 | 0.01 | 0.00 | 0.00 | 0.00 | 0.00 | 0.00 | 0.00 |
| Ammoniphilus | 0.00 | 0.00 | 0.00 | 0.13 | 0.00 | 0.00 | 0.00 | 0.00 | 0.00 | 0.00 | 0.00 | 0.00 | 0.00 | 0.00 | 0.00 | 0.00 | 0.00 | 0.00 | 0.00 | 0.00 | 0.00 |
| Brevibacillus | 0.00 | 0.00 | 0.00 | 0.00 | 0.00 | 0.00 | 0.00 | 0.00 | 0.00 | 0.00 | 0.00 | 0.00 | 0.02 | 0.00 | 0.00 | 0.00 | 0.00 | 0.00 | 0.00 | 0.00 | 0.00 |
| Paenibacillus | 0.00 | 0.00 | 0.00 | 0.13 | 0.00 | 0.00 | 0.00 | 0.00 | 0.00 | 0.00 | 0.00 | 0.01 | 0.00 | 0.00 | 0.00 | 0.00 | 0.00 | 0.00 | 0.01 | 0.00 | 0.00 |
| Chryseomicrobium | 0.00 | 0.00 | 0.00 | 0.00 | 0.06 | 0.00 | 0.00 | 0.00 | 0.00 | 0.00 | 0.00 | 0.00 | 0.00 | 0.00 | 0.00 | 0.00 | 0.00 | 0.00 | 0.00 | 0.00 | 0.00 |
| Lysinibacillus | 0.03 | 0.25 | 0.11 | 0.39 | 0.21 | 0.05 | 0.30 | 0.03 | 0.01 | 0.21 | 0.01 | 0.09 | 0.20 | 0.22 | 0.05 | 0.00 | 0.00 | 0.01 | 0.00 | 0.00 | 0.00 |
| Paenisporosarcina | 0.00 | 0.00 | 0.00 | 0.13 | 0.00 | 0.00 | 0.00 | 0.00 | 0.00 | 0.00 | 0.00 | 0.00 | 0.00 | 0.00 | 0.00 | 0.00 | 0.00 | 0.00 | 0.00 | 0.00 | 0.00 |
| Planococcus | 0.00 | 0.00 | 0.11 | 0.00 | 0.00 | 0.00 | 0.02 | 0.00 | 0.00 | 0.00 | 0.00 | 0.00 | 0.00 | 0.00 | 0.00 | 0.00 | 0.00 | 0.00 | 0.00 | 0.00 | 0.00 |
| unclassified_Sporolactobacillaceae | 0.00 | 0.04 | 0.00 | 0.00 | 0.00 | 0.00 | 0.00 | 0.00 | 0.00 | 0.03 | 0.00 | 0.00 | 0.00 | 0.00 | 0.00 | 0.00 | 0.00 | 0.00 | 0.00 | 0.00 | 0.00 |
| Staphylococcus | 0.05 | 0.08 | 0.02 | 0.00 | 0.07 | 0.04 | 0.11 | 0.00 | 0.01 | 0.05 | 0.00 | 0.02 | 0.08 | 0.10 | 0.08 | 0.00 | 0.00 | 0.00 | 0.00 | 0.00 | 0.00 |
| Aerococcus | 0.02 | 0.01 | 0.06 | 0.27 | 0.00 | 0.05 | 0.02 | 0.00 | 0.00 | 0.05 | 0.00 | 0.00 | 0.01 | 0.07 | 0.02 | 0.00 | 0.00 | 0.00 | 0.01 | 0.00 | 0.00 |
| Alloiococcus | 0.00 | 0.00 | 0.00 | 0.00 | 0.01 | 0.00 | 0.00 | 0.00 | 0.00 | 0.00 | 0.00 | 0.00 | 0.00 | 0.00 | 0.00 | 0.00 | 0.00 | 0.00 | 0.00 | 0.00 | 0.00 |
| Granulicatella | 0.00 | 0.00 | 0.02 | 0.00 | 0.00 | 0.00 | 0.00 | 0.01 | 0.00 | 0.04 | 0.00 | 0.00 | 0.00 | 0.00 | 0.00 | 0.00 | 0.00 | 0.00 | 0.00 | 0.00 | 0.00 |
| Trichococcus | 0.00 | 0.00 | 0.00 | 0.00 | 0.00 | 0.00 | 0.11 | 0.00 | 0.00 | 0.00 | 0.00 | 0.00 | 0.07 | 0.00 | 0.01 | 0.00 | 0.00 | 0.00 | 0.00 | 0.00 | 0.00 |
| Enterococcus | 0.01 | 0.04 | 0.08 | 0.06 | 0.19 | 0.20 | 0.04 | 0.01 | 0.01 | 0.03 | 0.02 | 0.03 | 0.05 | 0.01 | 0.02 | 0.00 | 0.00 | 0.00 | 0.00 | 0.00 | 0.00 |
| Lactobacillus | 0.00 | 0.02 | 0.60 | 1.31 | 0.02 | 0.38 | 0.19 | 0.06 | 0.01 | 0.19 | 0.04 | 0.05 | 0.01 | 0.30 | 0.03 | 0.00 | 0.00 | 0.00 | 0.01 | 0.01 | 0.00 |
| Pediococcus | 0.00 | 0.03 | 0.00 | 0.06 | 0.15 | 0.00 | 0.04 | 0.01 | 0.00 | 0.00 | 0.00 | 0.01 | 0.00 | 0.00 | 0.00 | 0.00 | 0.00 | 0.00 | 0.00 | 0.00 | 0.00 |
| Leuconostoc | 0.00 | 0.00 | 0.00 | 0.00 | 0.00 | 0.00 | 0.02 | 0.00 | 0.00 | 0.00 | 0.00 | 0.00 | 0.00 | 0.00 | 0.00 | 0.00 | 0.00 | 0.00 | 0.00 | 0.00 | 0.00 |
| Weissella | 0.00 | 0.00 | 0.00 | 0.00 | 0.00 | 0.00 | 0.00 | 0.00 | 0.00 | 0.00 | 0.00 | 0.02 | 0.00 | 0.00 | 0.00 | 0.00 | 0.00 | 0.00 | 0.00 | 0.00 | 0.00 |
| Lactococcus | 0.00 | 0.04 | 0.05 | 0.00 | 0.18 | 0.00 | 0.00 | 0.00 | 0.00 | 0.00 | 0.00 | 0.00 | 0.00 | 0.00 | 0.00 | 0.00 | 0.00 | 0.00 | 0.00 | 0.00 | 0.00 |
| Streptococcus | 0.04 | 0.21 | 0.07 | 0.32 | 0.27 | 0.18 | 0.11 | 0.02 | 0.01 | 0.15 | 0.01 | 0.01 | 0.05 | 0.17 | 0.03 | 0.00 | 0.00 | 0.00 | 0.00 | 0.01 | 0.00 |
| Candidatus_Arthromitus | 0.00 | 0.00 | 0.00 | 0.00 | 0.00 | 0.00 | 0.05 | 0.00 | 0.00 | 0.00 | 0.00 | 0.00 | 0.00 | 0.00 | 0.00 | 0.00 | 0.00 | 0.00 | 0.00 | 0.00 | 0.00 |
| Clostridium_sensu_stricto_1 | 0.00 | 0.01 | 0.01 | 1.47 | 0.00 | 0.01 | 0.00 | 0.00 | 0.00 | 0.00 | 0.00 | 0.00 | 0.00 | 0.00 | 0.00 | 0.00 | 0.00 | 0.00 | 0.01 | 0.01 | 0.00 |
| Clostridium_sensu_stricto_12 | 0.01 | 0.00 | 0.00 | 0.05 | 0.00 | 0.00 | 0.00 | 0.00 | 0.00 | 0.00 | 0.00 | 0.00 | 0.00 | 0.01 | 0.00 | 0.00 | 0.00 | 0.00 | 0.00 | 0.00 | 0.00 |
| Clostridium_sensu_stricto_13 | 0.00 | 0.00 | 0.00 | 0.42 | 0.00 | 0.00 | 0.00 | 0.00 | 0.00 | 0.00 | 0.00 | 0.00 | 0.00 | 0.00 | 0.00 | 0.00 | 0.00 | 0.00 | 0.00 | 0.00 | 0.00 |
| Clostridium_sensu_stricto_7 | 0.03 | 0.14 | 0.46 | 0.39 | 0.48 | 0.44 | 0.08 | 0.04 | 0.01 | 0.28 | 0.02 | 0.10 | 0.58 | 0.22 | 0.04 | 0.01 | 0.00 | 0.00 | 0.01 | 0.00 | 0.00 |
| Clostridium_sensu_stricto_8 | 0.00 | 0.00 | 0.00 | 0.33 | 0.00 | 0.00 | 0.00 | 0.00 | 0.00 | 0.00 | 0.00 | 0.00 | 0.00 | 0.00 | 0.00 | 0.00 | 0.00 | 0.00 | 0.00 | 0.00 | 0.00 |
| Fonticella | 0.00 | 0.00 | 0.00 | 0.10 | 0.00 | 0.00 | 0.00 | 0.00 | 0.00 | 0.00 | 0.00 | 0.00 | 0.00 | 0.00 | 0.00 | 0.00 | 0.00 | 0.00 | 0.00 | 0.00 | 0.00 |
| unclassified_Clostridiales_vadinBB60_group | 0.00 | 0.00 | 0.00 | 0.00 | 0.05 | 0.00 | 0.02 | 0.00 | 0.00 | 0.00 | 0.00 | 0.00 | 0.00 | 0.00 | 0.00 | 0.00 | 0.00 | 0.00 | 0.00 | 0.00 | 0.00 |
| Defluviitaleaceae_UCG-011 | 0.00 | 0.00 | 0.00 | 0.00 | 0.00 | 0.00 | 0.01 | 0.00 | 0.01 | 0.00 | 0.00 | 0.00 | 0.00 | 0.00 | 0.00 | 0.03 | 0.00 | 0.01 | 0.01 | 0.03 | 0.01 |
| Alkalibacter | 0.00 | 0.00 | 0.00 | 0.00 | 0.00 | 0.00 | 0.02 | 0.00 | 0.00 | 0.00 | 0.00 | 0.00 | 0.00 | 0.00 | 0.00 | 0.00 | 0.00 | 0.00 | 0.00 | 0.00 | 0.00 |
| Anaerococcus | 0.00 | 0.00 | 0.02 | 0.15 | 0.00 | 0.00 | 0.00 | 0.00 | 0.00 | 0.00 | 0.00 | 0.00 | 0.00 | 0.00 | 0.00 | 0.00 | 0.00 | 0.00 | 0.00 | 0.00 | 0.00 |
| Finegoldia | 0.00 | 0.00 | 0.00 | 0.07 | 0.00 | 0.00 | 0.00 | 0.00 | 0.00 | 0.00 | 0.00 | 0.00 | 0.00 | 0.00 | 0.00 | 0.00 | 0.00 | 0.00 | 0.00 | 0.00 | 0.00 |
| Peptoniphilus | 0.00 | 0.00 | 0.07 | 0.00 | 0.00 | 0.00 | 0.00 | 0.00 | 0.00 | 0.00 | 0.00 | 0.00 | 0.00 | 0.00 | 0.00 | 0.00 | 0.00 | 0.00 | 0.00 | 0.00 | 0.00 |
| Fusibacter | 0.07 | 0.04 | 0.05 | 0.04 | 0.75 | 0.32 | 0.16 | 0.52 | 0.62 | 0.11 | 2.52 | 0.58 | 0.16 | 0.06 | 0.22 | 3.35 | 1.00 | 3.01 | 1.23 | 1.02 | 0.21 |
| Guggenheimella | 0.01 | 0.01 | 0.00 | 0.31 | 0.00 | 0.34 | 0.00 | 0.07 | 0.00 | 0.00 | 0.23 | 0.01 | 0.00 | 0.00 | 0.00 | 0.00 | 0.00 | 0.01 | 0.01 | 0.00 | 0.00 |
| unclassified_Family_XVIII | 0.00 | 0.00 | 0.00 | 0.00 | 0.06 | 0.00 | 0.00 | 0.00 | 0.00 | 0.00 | 0.00 | 0.00 | 0.00 | 0.00 | 0.00 | 0.00 | 0.00 | 0.00 | 0.00 | 0.00 | 0.00 |
| Hydrogenispora | 0.00 | 0.00 | 0.00 | 0.57 | 0.00 | 0.00 | 0.00 | 0.00 | 0.00 | 0.00 | 0.00 | 0.00 | 0.00 | 0.00 | 0.00 | 0.00 | 0.00 | 0.00 | 0.00 | 0.00 | 0.00 |
| unclassified_JTB215 | 0.14 | 0.19 | 0.13 | 0.00 | 0.00 | 0.00 | 0.00 | 0.00 | 0.00 | 0.00 | 0.00 | 0.00 | 0.00 | 0.00 | 0.00 | 0.00 | 0.00 | 0.00 | 0.00 | 0.01 | 0.00 |
| unclassified_Lachnospiraceae | 0.00 | 0.08 | 0.01 | 0.12 | 0.01 | 0.06 | 0.06 | 0.00 | 0.01 | 0.00 | 0.00 | 0.01 | 0.03 | 0.05 | 0.04 | 0.00 | 0.00 | 0.00 | 0.00 | 0.00 | 0.00 |
| ASF356 | 0.00 | 0.10 | 0.00 | 0.00 | 0.00 | 0.00 | 0.00 | 0.01 | 0.00 | 0.00 | 0.00 | 0.00 | 0.00 | 0.01 | 0.00 | 0.00 | 0.00 | 0.00 | 0.00 | 0.00 | 0.00 |
| Acetatifactor | 0.00 | 0.00 | 0.00 | 0.00 | 0.00 | 0.00 | 0.00 | 0.00 | 0.00 | 0.00 | 0.00 | 0.00 | 0.00 | 0.03 | 0.00 | 0.00 | 0.00 | 0.00 | 0.00 | 0.00 | 0.00 |
| Blautia | 0.02 | 0.00 | 0.00 | 0.00 | 0.00 | 0.00 | 0.00 | 0.00 | 0.00 | 0.00 | 0.02 | 0.00 | 0.00 | 0.00 | 0.01 | 0.00 | 0.00 | 0.00 | 0.00 | 0.00 | 0.00 |
| Catonella | 0.00 | 0.00 | 0.00 | 0.00 | 0.00 | 0.00 | 0.00 | 0.00 | 0.00 | 0.08 | 0.00 | 0.00 | 0.00 | 0.00 | 0.00 | 0.00 | 0.00 | 0.00 | 0.00 | 0.00 | 0.00 |
| Coprococcus_3 | 0.00 | 0.00 | 0.00 | 0.00 | 0.00 | 0.00 | 0.00 | 0.00 | 0.00 | 0.00 | 0.00 | 0.00 | 0.00 | 0.00 | 0.00 | 0.00 | 0.00 | 0.00 | 0.00 | 0.00 | 0.00 |
| Dorea | 0.00 | 0.00 | 0.00 | 0.00 | 0.00 | 0.00 | 0.01 | 0.00 | 0.00 | 0.00 | 0.00 | 0.00 | 0.00 | 0.00 | 0.00 | 0.00 | 0.00 | 0.00 | 0.00 | 0.00 | 0.00 |
| Epulopiscium | 0.00 | 0.00 | 0.00 | 0.15 | 0.00 | 0.00 | 0.00 | 0.00 | 0.00 | 0.00 | 0.00 | 0.00 | 0.00 | 0.00 | 0.00 | 0.00 | 0.00 | 0.00 | 0.00 | 0.00 | 0.00 |
| GCA-900066575 | 0.00 | 0.00 | 0.05 | 0.00 | 0.07 | 0.00 | 0.05 | 0.00 | 0.00 | 0.03 | 0.00 | 0.00 | 0.00 | 0.00 | 0.00 | 0.00 | 0.00 | 0.00 | 0.00 | 0.00 | 0.00 |
| Johnsonella | 0.00 | 0.00 | 0.00 | 0.00 | 0.00 | 0.00 | 0.00 | 0.00 | 0.00 | 0.01 | 0.00 | 0.00 | 0.00 | 0.00 | 0.00 | 0.00 | 0.00 | 0.00 | 0.00 | 0.00 | 0.00 |
| Lachnoclostridium | 0.01 | 0.06 | 0.02 | 0.00 | 0.00 | 0.00 | 0.05 | 0.00 | 0.00 | 0.01 | 0.02 | 0.00 | 0.00 | 0.00 | 0.01 | 0.00 | 0.00 | 0.00 | 0.00 | 0.00 | 0.00 |
| Lachnospira | 0.00 | 0.00 | 0.00 | 0.00 | 0.00 | 0.00 | 0.00 | 0.00 | 0.00 | 0.00 | 0.00 | 0.00 | 0.00 | 0.00 | 0.00 | 0.00 | 0.00 | 0.00 | 0.00 | 0.00 | 0.00 |
| Lachnospiraceae_NK4A136_group | 0.12 | 0.34 | 0.16 | 0.20 | 0.51 | 0.61 | 0.36 | 0.05 | 0.02 | 0.23 | 0.08 | 0.06 | 0.11 | 0.20 | 0.08 | 0.00 | 0.01 | 0.00 | 0.01 | 0.00 | 0.01 |
| Lachnospiraceae_UCG-001 | 0.00 | 0.03 | 0.00 | 0.00 | 0.00 | 0.00 | 0.00 | 0.00 | 0.00 | 0.00 | 0.00 | 0.00 | 0.00 | 0.00 | 0.00 | 0.00 | 0.00 | 0.00 | 0.00 | 0.00 | 0.00 |
| Lachnospiraceae_UCG-006 | 0.00 | 0.02 | 0.00 | 0.00 | 0.00 | 0.00 | 0.03 | 0.00 | 0.00 | 0.00 | 0.00 | 0.00 | 0.00 | 0.00 | 0.00 | 0.00 | 0.00 | 0.00 | 0.00 | 0.00 | 0.00 |
| Roseburia | 0.00 | 0.01 | 0.04 | 0.06 | 0.00 | 0.00 | 0.01 | 0.00 | 0.00 | 0.03 | 0.00 | 0.00 | 0.00 | 0.00 | 0.02 | 0.00 | 0.00 | 0.00 | 0.00 | 0.00 | 0.00 |
| Stomatobaculum | 0.00 | 0.00 | 0.00 | 0.00 | 0.00 | 0.00 | 0.01 | 0.00 | 0.00 | 0.00 | 0.00 | 0.00 | 0.00 | 0.00 | 0.00 | 0.00 | 0.00 | 0.00 | 0.00 | 0.00 | 0.00 |
| Tyzzerella | 0.00 | 0.00 | 0.00 | 0.14 | 0.00 | 0.00 | 0.04 | 0.01 | 0.00 | 0.00 | 0.00 | 0.00 | 0.00 | 0.00 | 0.00 | 0.00 | 0.00 | 0.00 | 0.00 | 0.00 | 0.00 |
| Tyzzerella_3 | 0.00 | 0.00 | 0.00 | 0.00 | 0.00 | 0.00 | 0.00 | 0.00 | 0.01 | 0.00 | 0.00 | 0.00 | 0.00 | 0.00 | 0.00 | 0.00 | 0.00 | 0.00 | 0.00 | 0.00 | 0.00 |
| [Eubacterium]eligens_group | 0.00 | 0.00 | 0.00 | 0.00 | 0.00 | 0.00 | 0.00 | 0.00 | 0.00 | 0.00 | 0.00 | 0.00 | 0.00 | 0.00 | 0.00 | 0.00 | 0.00 | 0.00 | 0.00 | 0.00 | 0.00 |
| [Eubacterium]fissicatena_group | 0.01 | 0.00 | 0.08 | 0.00 | 0.00 | 0.00 | 0.00 | 0.00 | 0.00 | 0.00 | 0.01 | 0.00 | 0.00 | 0.00 | 0.00 | 0.00 | 0.00 | 0.00 | 0.00 | 0.00 | 0.00 |
| [Eubacterium]xylanophilum_group | 0.00 | 0.09 | 0.00 | 0.00 | 0.00 | 0.00 | 0.01 | 0.00 | 0.00 | 0.03 | 0.00 | 0.00 | 0.00 | 0.00 | 0.00 | 0.00 | 0.00 | 0.00 | 0.00 | 0.00 | 0.00 |
| [Ruminococcus]torques_group | 0.00 | 0.00 | 0.00 | 0.00 | 0.00 | 0.00 | 0.00 | 0.00 | 0.00 | 0.00 | 0.00 | 0.00 | 0.00 | 0.00 | 0.00 | 0.00 | 0.00 | 0.00 | 0.00 | 0.00 | 0.00 |
| Peptococcus | 0.02 | 0.00 | 0.00 | 0.00 | 0.00 | 0.00 | 0.00 | 0.00 | 0.00 | 0.00 | 0.00 | 0.00 | 0.00 | 0.00 | 0.00 | 0.00 | 0.00 | 0.00 | 0.00 | 0.00 | 0.00 |
| Syntrophobotulus | 0.00 | 0.00 | 0.00 | 0.21 | 0.00 | 0.00 | 0.00 | 0.00 | 0.00 | 0.00 | 0.00 | 0.00 | 0.00 | 0.00 | 0.00 | 0.00 | 0.00 | 0.00 | 0.00 | 0.00 | 0.00 |
| unclassified_Peptostreptococcaceae | 0.00 | 0.00 | 0.05 | 0.00 | 0.00 | 0.00 | 0.00 | 0.00 | 0.00 | 0.00 | 0.00 | 0.00 | 0.00 | 0.00 | 0.00 | 0.00 | 0.00 | 0.00 | 0.00 | 0.00 | 0.00 |
| Paraclostridium | 0.00 | 0.00 | 0.00 | 1.60 | 0.00 | 0.01 | 0.00 | 0.00 | 0.00 | 0.02 | 0.00 | 0.00 | 0.00 | 0.01 | 0.01 | 0.01 | 0.00 | 0.00 | 0.00 | 0.00 | 0.00 |
| Peptoclostridium | 0.00 | 0.00 | 0.00 | 0.00 | 0.00 | 0.00 | 0.00 | 0.00 | 0.00 | 0.00 | 0.00 | 0.00 | 0.00 | 0.00 | 0.00 | 0.00 | 0.00 | 0.00 | 0.00 | 0.00 | 0.00 |
| Peptostreptococcus | 0.00 | 0.00 | 0.00 | 0.00 | 0.00 | 0.00 | 0.00 | 0.00 | 0.00 | 0.01 | 0.00 | 0.00 | 0.01 | 0.00 | 0.01 | 0.00 | 0.00 | 0.00 | 0.00 | 0.00 | 0.00 |
| Sporacetigenium | 0.00 | 0.00 | 0.00 | 0.38 | 0.00 | 0.00 | 0.01 | 0.00 | 0.00 | 0.00 | 0.00 | 0.00 | 0.00 | 0.00 | 0.00 | 0.00 | 0.00 | 0.00 | 0.00 | 0.00 | 0.00 |
| Anaerotruncus | 0.01 | 0.00 | 0.00 | 0.00 | 0.00 | 0.00 | 0.00 | 0.00 | 0.00 | 0.00 | 0.00 | 0.00 | 0.00 | 0.00 | 0.01 | 0.00 | 0.00 | 0.00 | 0.00 | 0.00 | 0.00 |
| Faecalibacterium | 0.00 | 0.00 | 0.18 | 0.00 | 0.00 | 0.00 | 0.00 | 0.00 | 0.00 | 0.00 | 0.00 | 0.00 | 0.00 | 0.00 | 0.00 | 0.00 | 0.00 | 0.00 | 0.00 | 0.00 | 0.00 |
| Intestinimonas | 0.00 | 0.00 | 0.00 | 0.00 | 0.00 | 0.00 | 0.09 | 0.00 | 0.00 | 0.00 | 0.00 | 0.01 | 0.05 | 0.00 | 0.00 | 0.00 | 0.00 | 0.00 | 0.00 | 0.00 | 0.00 |
| Negativibacillus | 0.00 | 0.00 | 0.00 | 0.00 | 0.00 | 0.00 | 0.00 | 0.00 | 0.00 | 0.00 | 0.00 | 0.00 | 0.00 | 0.00 | 0.00 | 0.00 | 0.00 | 0.00 | 0.00 | 0.00 | 0.00 |
| Oscillibacter | 0.00 | 0.00 | 0.00 | 0.00 | 0.00 | 0.00 | 0.03 | 0.01 | 0.00 | 0.00 | 0.00 | 0.00 | 0.00 | 0.00 | 0.00 | 0.00 | 0.00 | 0.00 | 0.00 | 0.00 | 0.00 |
| Ruminiclostridium | 0.01 | 0.01 | 0.01 | 0.09 | 0.02 | 0.00 | 0.07 | 0.00 | 0.01 | 0.00 | 0.00 | 0.00 | 0.01 | 0.02 | 0.00 | 0.00 | 0.00 | 0.00 | 0.00 | 0.00 | 0.00 |
| Ruminiclostridium_5 | 0.00 | 0.01 | 0.00 | 0.02 | 0.00 | 0.00 | 0.00 | 0.00 | 0.00 | 0.00 | 0.02 | 0.00 | 0.03 | 0.00 | 0.00 | 0.00 | 0.00 | 0.00 | 0.00 | 0.00 | 0.00 |
| Ruminiclostridium_9 | 0.00 | 0.09 | 0.00 | 0.00 | 0.16 | 0.00 | 0.05 | 0.01 | 0.00 | 0.00 | 0.00 | 0.01 | 0.02 | 0.00 | 0.00 | 0.00 | 0.00 | 0.00 | 0.00 | 0.00 | 0.00 |
| Ruminococcaceae_NK4A214_group | 0.00 | 0.00 | 0.02 | 0.00 | 0.03 | 0.00 | 0.00 | 0.00 | 0.00 | 0.00 | 0.00 | 0.00 | 0.00 | 0.00 | 0.00 | 0.00 | 0.00 | 0.00 | 0.00 | 0.00 | 0.00 |
| Ruminococcaceae_UCG-010 | 0.00 | 0.00 | 0.00 | 0.00 | 0.00 | 0.00 | 0.01 | 0.00 | 0.00 | 0.00 | 0.00 | 0.00 | 0.00 | 0.00 | 0.02 | 0.00 | 0.00 | 0.00 | 0.00 | 0.00 | 0.00 |
| Ruminococcaceae_UCG-014 | 0.01 | 0.00 | 0.02 | 0.06 | 0.12 | 0.00 | 0.00 | 0.00 | 0.00 | 0.00 | 0.00 | 0.00 | 0.04 | 0.05 | 0.01 | 0.00 | 0.00 | 0.00 | 0.00 | 0.00 | 0.00 |
| Ruminococcus_1 | 0.00 | 0.00 | 0.00 | 0.00 | 0.00 | 0.00 | 0.00 | 0.00 | 0.00 | 0.01 | 0.00 | 0.00 | 0.00 | 0.00 | 0.00 | 0.00 | 0.00 | 0.00 | 0.00 | 0.00 | 0.00 |
| [Eubacterium]coprostanoligenes_group | 0.00 | 0.00 | 0.00 | 0.00 | 0.00 | 0.00 | 0.31 | 0.00 | 0.00 | 0.00 | 0.00 | 0.00 | 0.00 | 0.05 | 0.00 | 0.00 | 0.00 | 0.00 | 0.00 | 0.00 | 0.00 |
| unclassified_TSAC18 | 0.00 | 0.00 | 0.00 | 0.00 | 0.00 | 0.00 | 0.00 | 0.00 | 0.00 | 0.00 | 0.00 | 0.00 | 0.00 | 0.00 | 0.00 | 0.00 | 0.00 | 0.00 | 0.00 | 0.00 | 0.00 |
| unclassified_Erysipelotrichaceae | 0.00 | 0.00 | 0.00 | 0.00 | 0.00 | 0.00 | 0.00 | 0.00 | 0.00 | 0.01 | 0.00 | 0.00 | 0.00 | 0.00 | 0.00 | 0.00 | 0.00 | 0.00 | 0.00 | 0.00 | 0.00 |
| Allobaculum | 0.00 | 0.00 | 0.00 | 0.00 | 0.00 | 0.00 | 0.06 | 0.00 | 0.00 | 0.00 | 0.00 | 0.00 | 0.00 | 0.00 | 0.00 | 0.00 | 0.00 | 0.00 | 0.00 | 0.00 | 0.00 |
| Erysipelatoclostridium | 0.00 | 0.00 | 0.00 | 0.00 | 0.00 | 0.00 | 0.04 | 0.00 | 0.00 | 0.00 | 0.00 | 0.00 | 0.00 | 0.00 | 0.00 | 0.00 | 0.00 | 0.00 | 0.00 | 0.00 | 0.00 |
| Turicibacter | 0.00 | 0.00 | 0.00 | 0.00 | 0.00 | 0.00 | 0.00 | 0.00 | 0.00 | 0.00 | 0.00 | 0.00 | 0.00 | 0.00 | 0.00 | 0.00 | 0.00 | 0.00 | 0.00 | 0.00 | 0.00 |
| [Anaerorhabdus]furcosa_group | 0.00 | 0.00 | 0.00 | 0.00 | 0.00 | 0.00 | 0.04 | 0.00 | 0.00 | 0.00 | 0.00 | 0.00 | 0.00 | 0.00 | 0.00 | 0.01 | 0.00 | 0.00 | 0.00 | 0.00 | 0.00 |
| unclassified_Acidaminococcaceae | 0.00 | 0.00 | 0.00 | 0.00 | 0.00 | 0.00 | 0.01 | 0.00 | 0.00 | 0.00 | 0.00 | 0.00 | 0.00 | 0.00 | 0.00 | 0.00 | 0.00 | 0.00 | 0.00 | 0.00 | 0.00 |
| Dialister | 0.00 | 0.00 | 0.00 | 0.00 | 0.00 | 0.00 | 0.00 | 0.00 | 0.00 | 0.00 | 0.00 | 0.00 | 0.00 | 0.00 | 0.00 | 0.00 | 0.00 | 0.00 | 0.00 | 0.00 | 0.00 |
| Megasphaera | 0.00 | 0.00 | 0.02 | 0.00 | 0.00 | 0.00 | 0.00 | 0.00 | 0.00 | 0.00 | 0.00 | 0.00 | 0.00 | 0.00 | 0.00 | 0.00 | 0.00 | 0.00 | 0.00 | 0.00 | 0.00 |
| Selenomonas_3 | 0.00 | 0.00 | 0.00 | 0.00 | 0.00 | 0.00 | 0.00 | 0.00 | 0.00 | 0.00 | 0.00 | 0.00 | 0.00 | 0.01 | 0.00 | 0.00 | 0.00 | 0.00 | 0.00 | 0.00 | 0.00 |
| Veillonella | 0.00 | 0.03 | 0.00 | 0.00 | 0.00 | 0.08 | 0.00 | 0.00 | 0.00 | 0.06 | 0.00 | 0.00 | 0.04 | 0.00 | 0.00 | 0.00 | 0.00 | 0.00 | 0.00 | 0.00 | 0.00 |
| Fusobacterium | 0.05 | 0.00 | 0.01 | 0.00 | 0.16 | 0.00 | 0.06 | 0.01 | 3.09 | 0.00 | 0.00 | 0.01 | 0.06 | 0.00 | 0.00 | 0.01 | 0.01 | 0.00 | 0.01 | 0.01 | 0.03 |
| Propionigenium | 0.01 | 0.01 | 0.01 | 0.00 | 0.00 | 0.01 | 0.00 | 0.00 | 2.45 | 0.00 | 0.16 | 0.89 | 0.02 | 0.01 | 0.01 | 0.41 | 0.18 | 0.12 | 2.76 | 0.03 | 0.17 |
| Psychrilyobacter | 1.09 | 0.40 | 0.61 | 0.10 | 0.10 | 0.32 | 0.11 | 0.23 | 5.55 | 0.10 | 0.16 | 0.75 | 0.22 | 0.15 | 0.24 | 0.20 | 0.60 | 0.46 | 5.65 | 0.21 | 48.20 |
| Leptotrichia | 0.00 | 0.00 | 0.10 | 0.00 | 0.00 | 0.00 | 0.00 | 0.00 | 0.00 | 0.00 | 0.00 | 0.02 | 0.00 | 0.00 | 0.00 | 0.00 | 0.00 | 0.00 | 0.00 | 0.00 | 0.00 |
| unclassified_GAL15 | 0.00 | 0.00 | 0.00 | 0.17 | 0.00 | 0.00 | 0.00 | 0.00 | 0.00 | 0.00 | 0.00 | 0.00 | 0.00 | 0.00 | 0.00 | 0.00 | 0.00 | 0.00 | 0.00 | 0.00 | 0.00 |
| unclassified_BD2-11_terrestrial_group | 0.00 | 0.00 | 0.00 | 0.00 | 0.00 | 0.00 | 0.02 | 0.00 | 0.00 | 0.00 | 0.00 | 0.00 | 0.00 | 0.00 | 0.00 | 0.00 | 0.00 | 0.00 | 0.00 | 0.00 | 0.00 |
| unclassified_Gemmatimonadaceae | 0.00 | 0.00 | 0.01 | 0.00 | 0.01 | 0.01 | 0.01 | 0.00 | 0.00 | 0.00 | 0.00 | 0.00 | 0.02 | 0.00 | 0.00 | 0.01 | 0.00 | 0.00 | 0.00 | 0.01 | 0.00 |
| Gemmatimonas | 0.00 | 0.00 | 0.01 | 0.00 | 0.00 | 0.00 | 0.00 | 0.00 | 0.00 | 0.00 | 0.00 | 0.00 | 0.00 | 0.01 | 0.00 | 0.01 | 0.01 | 0.01 | 0.00 | 0.01 | 0.00 |
| Gemmatirosa | 0.00 | 0.00 | 0.00 | 0.00 | 0.00 | 0.00 | 0.00 | 0.00 | 0.00 | 0.00 | 0.00 | 0.00 | 0.00 | 0.00 | 0.00 | 0.00 | 0.00 | 0.00 | 0.00 | 0.00 | 0.00 |
| unclassified_Longimicrobiaceae | 0.00 | 0.00 | 0.00 | 0.00 | 0.00 | 0.00 | 0.00 | 0.00 | 0.00 | 0.00 | 0.00 | 0.00 | 0.00 | 0.00 | 0.00 | 0.00 | 0.00 | 0.00 | 0.00 | 0.00 | 0.00 |
| unclassified_PAUC43f_marine_benthic_group | 0.00 | 0.00 | 0.00 | 0.00 | 0.00 | 0.00 | 0.02 | 0.00 | 0.00 | 0.00 | 0.00 | 0.00 | 0.00 | 0.00 | 0.00 | 0.00 | 0.00 | 0.00 | 0.00 | 0.00 | 0.00 |
| unclassified_S0134_terrestrial_group | 0.00 | 0.00 | 0.00 | 0.00 | 0.00 | 0.00 | 0.00 | 0.00 | 0.00 | 0.00 | 0.00 | 0.00 | 0.00 | 0.00 | 0.00 | 0.00 | 0.00 | 0.00 | 0.00 | 0.00 | 0.00 |
| unclassified_Hydrogenedensaceae | 0.00 | 0.00 | 0.00 | 0.00 | 0.00 | 0.00 | 0.01 | 0.00 | 0.00 | 0.00 | 0.00 | 0.00 | 0.00 | 0.00 | 0.00 | 0.00 | 0.00 | 0.00 | 0.00 | 0.00 | 0.00 |
| unclassified_Latescibacteraceae | 0.00 | 0.00 | 0.00 | 0.00 | 0.00 | 0.00 | 0.00 | 0.00 | 0.00 | 0.00 | 0.00 | 0.00 | 0.00 | 0.00 | 0.00 | 0.00 | 0.00 | 0.00 | 0.00 | 0.00 | 0.00 |
| unclassified_Margulisbacteria | 0.01 | 0.00 | 0.00 | 0.00 | 0.00 | 0.00 | 0.00 | 0.00 | 0.00 | 0.00 | 0.00 | 0.00 | 0.00 | 0.00 | 0.00 | 0.00 | 0.00 | 0.00 | 0.00 | 0.00 | 0.00 |
| Nitrospina | 0.00 | 0.00 | 0.00 | 0.00 | 0.00 | 0.00 | 0.00 | 0.00 | 0.00 | 0.00 | 0.00 | 0.00 | 0.02 | 0.00 | 0.05 | 0.00 | 0.00 | 0.00 | 0.00 | 0.00 | 0.00 |
| Leptospirillum | 0.01 | 0.01 | 0.01 | 0.00 | 0.00 | 0.00 | 0.00 | 0.00 | 0.00 | 0.00 | 0.00 | 0.00 | 0.00 | 0.00 | 0.00 | 0.00 | 0.00 | 0.00 | 0.00 | 0.00 | 0.00 |
| Nitrospira | 0.00 | 0.00 | 0.00 | 0.21 | 0.00 | 0.00 | 0.00 | 0.00 | 0.00 | 0.00 | 0.02 | 0.00 | 0.00 | 0.00 | 0.00 | 0.00 | 0.00 | 0.00 | 0.00 | 0.00 | 0.00 |
| unclassified_Thermodesulfovibrionia | 0.00 | 0.00 | 0.00 | 0.00 | 0.00 | 0.00 | 0.03 | 0.00 | 0.00 | 0.00 | 0.00 | 0.00 | 0.00 | 0.00 | 0.00 | 0.00 | 0.00 | 0.00 | 0.00 | 0.00 | 0.00 |
| unclassified_Omnitrophicaeota | 0.00 | 0.00 | 0.00 | 0.00 | 0.00 | 0.00 | 0.00 | 0.00 | 0.00 | 0.00 | 0.00 | 0.00 | 0.00 | 0.00 | 0.00 | 0.00 | 0.00 | 0.00 | 0.00 | 0.00 | 0.00 |
| unclassified_Candidatus_Magasanikbacteria | 0.12 | 0.01 | 0.17 | 0.09 | 0.13 | 0.43 | 0.00 | 0.00 | 0.00 | 0.00 | 0.00 | 0.00 | 0.00 | 0.00 | 0.00 | 0.00 | 0.00 | 0.00 | 0.00 | 0.00 | 0.00 |
| Candidatus_Berkelbacteria_bacterium_RIFOXYA2_FULL_43_10 | 0.00 | 0.00 | 0.00 | 0.00 | 0.00 | 0.00 | 0.00 | 0.00 | 0.00 | 0.15 | 0.00 | 0.00 | 0.00 | 0.00 | 0.00 | 0.00 | 0.00 | 0.00 | 0.00 | 0.00 | 0.00 |
| unclassified_Gracilibacteria | 0.00 | 0.00 | 0.03 | 0.00 | 0.00 | 0.00 | 0.00 | 0.00 | 0.00 | 0.02 | 0.07 | 0.14 | 0.01 | 0.02 | 0.02 | 0.10 | 0.07 | 0.22 | 1.20 | 0.46 | 0.06 |
| unclassified_Candidatus_Peregrinibacteria | 0.01 | 0.00 | 0.03 | 0.00 | 0.00 | 0.00 | 0.00 | 0.00 | 0.00 | 0.00 | 0.01 | 0.00 | 0.04 | 0.00 | 0.11 | 0.00 | 0.00 | 0.00 | 0.05 | 0.01 | 0.01 |
| unclassified_JGI_0000069-P22 | 0.13 | 0.38 | 0.22 | 0.00 | 0.00 | 0.00 | 0.00 | 0.11 | 0.04 | 0.00 | 0.00 | 0.04 | 0.13 | 0.00 | 0.05 | 0.00 | 0.01 | 0.05 | 0.01 | 0.01 | 0.00 |
| unclassified_Candidatus_Pacebacteria | 0.07 | 0.00 | 0.13 | 0.06 | 0.21 | 0.00 | 0.00 | 0.00 | 0.00 | 0.00 | 0.00 | 0.00 | 0.00 | 0.00 | 0.05 | 0.00 | 0.00 | 0.00 | 0.00 | 0.00 | 0.00 |
| unclassified_Candidatus_Campbellbacteria | 0.12 | 0.17 | 0.01 | 0.43 | 0.09 | 0.04 | 2.03 | 13.78 | 8.53 | 0.22 | 0.37 | 0.40 | 6.74 | 2.50 | 17.34 | 0.00 | 0.03 | 0.16 | 0.04 | 0.02 | 0.01 |
| Candidatus_Nomurabacteria_bacterium_RIFCSPLOWO2_01_FULL_36_10b | 0.53 | 0.52 | 0.65 | 0.00 | 0.00 | 0.17 | 0.00 | 0.00 | 0.00 | 0.00 | 0.13 | 0.03 | 0.00 | 0.00 | 0.01 | 0.00 | 0.01 | 0.02 | 0.00 | 0.00 | 0.00 |
| unclassified_Candidatus_Kaiserbacteria | 0.04 | 0.09 | 0.00 | 0.64 | 0.00 | 0.67 | 0.00 | 0.00 | 0.00 | 0.00 | 0.00 | 0.10 | 0.00 | 0.04 | 0.00 | 0.00 | 0.02 | 0.12 | 0.00 | 0.00 | 0.00 |
| unclassified_Candidatus_Moranbacteria | 0.00 | 0.00 | 0.00 | 0.00 | 0.00 | 0.00 | 0.00 | 0.00 | 0.00 | 0.00 | 0.00 | 0.00 | 0.00 | 0.00 | 0.00 | 0.00 | 0.00 | 0.00 | 0.00 | 0.00 | 0.00 |
| unclassified_Saccharimonadales | 0.00 | 0.10 | 0.10 | 0.01 | 0.09 | 0.00 | 0.02 | 0.00 | 0.00 | 0.00 | 0.00 | 0.00 | 0.03 | 0.00 | 0.02 | 0.00 | 0.00 | 0.00 | 0.00 | 0.00 | 0.00 |
| unclassified_Saccharimonadaceae | 0.00 | 0.00 | 0.00 | 0.00 | 0.00 | 0.00 | 0.05 | 0.00 | 0.00 | 0.02 | 0.00 | 0.00 | 0.00 | 0.00 | 0.00 | 0.00 | 0.00 | 0.00 | 0.00 | 0.00 | 0.00 |
| Candidatus_Saccharimonas | 0.00 | 0.00 | 0.00 | 0.23 | 0.00 | 0.00 | 0.00 | 0.00 | 0.00 | 0.00 | 0.00 | 0.00 | 0.00 | 0.00 | 0.00 | 0.00 | 0.00 | 0.00 | 0.00 | 0.00 | 0.00 |
| unclassified_WS6_[Dojkabacteria] | 0.00 | 0.00 | 0.00 | 0.00 | 0.00 | 0.00 | 0.02 | 0.00 | 0.00 | 0.00 | 0.00 | 0.00 | 0.00 | 0.00 | 0.00 | 0.00 | 0.00 | 0.00 | 0.00 | 0.00 | 0.00 |
| candidate_division_WS6_bacterium_GW2011_GWE2_33_157 | 0.00 | 0.00 | 0.00 | 0.00 | 0.00 | 0.00 | 0.00 | 0.00 | 0.00 | 0.00 | 0.00 | 0.00 | 0.00 | 0.00 | 0.00 | 0.00 | 0.00 | 0.00 | 0.00 | 0.00 | 0.00 |
| unclassified_BD7-11 | 0.00 | 0.00 | 0.00 | 0.00 | 0.08 | 0.18 | 0.00 | 0.00 | 0.01 | 0.00 | 0.00 | 0.00 | 0.00 | 0.07 | 0.02 | 0.00 | 0.00 | 0.00 | 0.00 | 0.00 | 0.00 |
| Candidatus_Brocadia | 0.00 | 0.00 | 0.00 | 0.00 | 0.07 | 0.00 | 0.03 | 0.00 | 0.00 | 0.00 | 0.00 | 0.00 | 0.00 | 0.00 | 0.00 | 0.00 | 0.00 | 0.00 | 0.00 | 0.00 | 0.00 |
| Candidatus_Kuenenia | 0.00 | 0.00 | 0.00 | 0.00 | 0.15 | 0.00 | 0.00 | 0.00 | 0.00 | 0.00 | 0.00 | 0.00 | 0.00 | 0.00 | 0.00 | 0.00 | 0.00 | 0.00 | 0.00 | 0.00 | 0.00 |
| unclassified_OM190 | 0.01 | 0.02 | 0.08 | 0.05 | 0.04 | 0.01 | 0.18 | 0.02 | 0.07 | 0.01 | 0.05 | 0.06 | 0.02 | 0.01 | 0.02 | 0.01 | 0.02 | 0.02 | 0.01 | 0.02 | 0.01 |
| unclassified_Phycisphaeraceae | 0.00 | 0.00 | 0.00 | 0.00 | 0.00 | 0.00 | 0.00 | 0.00 | 0.00 | 0.00 | 0.04 | 0.00 | 0.00 | 0.00 | 0.00 | 0.00 | 0.00 | 0.00 | 0.00 | 0.00 | 0.00 |
| Phycisphaera | 0.02 | 0.01 | 0.00 | 0.00 | 0.00 | 0.01 | 0.00 | 0.02 | 0.01 | 0.00 | 0.07 | 0.06 | 0.00 | 0.00 | 0.01 | 0.00 | 0.00 | 0.01 | 0.01 | 0.00 | 0.00 |
| SM1A02 | 0.00 | 0.00 | 0.00 | 0.00 | 0.00 | 0.00 | 0.00 | 0.00 | 0.00 | 0.00 | 0.00 | 0.03 | 0.00 | 0.00 | 0.00 | 0.00 | 0.00 | 0.00 | 0.00 | 0.00 | 0.00 |
| unclassified_Pla4_lineage | 0.00 | 0.00 | 0.00 | 0.00 | 0.00 | 0.00 | 0.01 | 0.00 | 0.00 | 0.00 | 0.00 | 0.00 | 0.00 | 0.00 | 0.00 | 0.00 | 0.00 | 0.00 | 0.00 | 0.00 | 0.00 |
| unclassified_Gemmataceae | 0.00 | 0.00 | 0.00 | 0.00 | 0.00 | 0.00 | 0.00 | 0.00 | 0.00 | 0.00 | 0.01 | 0.00 | 0.00 | 0.00 | 0.00 | 0.00 | 0.00 | 0.00 | 0.00 | 0.00 | 0.00 |
| Aquisphaera | 0.00 | 0.00 | 0.00 | 0.00 | 0.00 | 0.00 | 0.00 | 0.00 | 0.00 | 0.00 | 0.00 | 0.00 | 0.00 | 0.00 | 0.00 | 0.00 | 0.00 | 0.00 | 0.00 | 0.00 | 0.00 |
| Pirellula | 0.00 | 0.00 | 0.00 | 0.00 | 0.00 | 0.00 | 0.00 | 0.00 | 0.00 | 0.00 | 0.01 | 0.00 | 0.00 | 0.00 | 0.00 | 0.00 | 0.00 | 0.00 | 0.00 | 0.00 | 0.00 |
| unclassified_Gimesiaceae | 0.00 | 0.00 | 0.00 | 0.00 | 0.00 | 0.00 | 0.00 | 0.00 | 0.00 | 0.00 | 0.00 | 0.00 | 0.00 | 0.00 | 0.00 | 0.00 | 0.00 | 0.00 | 0.00 | 0.00 | 0.00 |
| Fuerstia | 0.00 | 0.00 | 0.00 | 0.00 | 0.00 | 0.00 | 0.00 | 0.00 | 0.00 | 0.00 | 0.00 | 0.00 | 0.00 | 0.02 | 0.00 | 0.00 | 0.00 | 0.00 | 0.00 | 0.00 | 0.00 |
| unclassified_Alphaproteobacteria | 0.00 | 0.00 | 0.00 | 0.00 | 0.00 | 0.00 | 0.01 | 0.00 | 0.00 | 0.00 | 0.00 | 0.01 | 0.00 | 0.00 | 0.00 | 0.00 | 0.00 | 0.00 | 0.00 | 0.00 | 0.00 |
| Commensalibacter | 0.00 | 0.00 | 0.00 | 0.00 | 0.00 | 0.00 | 0.04 | 0.00 | 0.00 | 0.00 | 0.00 | 0.00 | 0.00 | 0.00 | 0.00 | 0.00 | 0.00 | 0.00 | 0.00 | 0.00 | 0.00 |
| Craurococcus | 0.00 | 0.00 | 0.09 | 0.00 | 0.00 | 0.00 | 0.00 | 0.00 | 0.00 | 0.00 | 0.00 | 0.00 | 0.00 | 0.00 | 0.00 | 0.00 | 0.00 | 0.00 | 0.00 | 0.00 | 0.00 |
| Roseomonas | 0.03 | 0.00 | 0.00 | 0.00 | 0.00 | 0.00 | 0.00 | 0.00 | 0.00 | 0.00 | 0.00 | 0.00 | 0.00 | 0.00 | 0.00 | 0.00 | 0.00 | 0.00 | 0.00 | 0.00 | 0.00 |
| unclassified_Caedibacteraceae | 0.00 | 0.00 | 0.00 | 0.00 | 0.00 | 0.00 | 0.01 | 0.00 | 0.00 | 0.00 | 0.00 | 0.00 | 0.00 | 0.00 | 0.00 | 0.00 | 0.00 | 0.00 | 0.00 | 0.00 | 0.00 |
| unclassified_Caulobacteraceae | 0.04 | 0.00 | 0.13 | 0.06 | 0.03 | 0.00 | 0.00 | 0.00 | 0.01 | 0.00 | 0.02 | 0.00 | 0.01 | 0.00 | 0.00 | 0.00 | 0.00 | 0.00 | 0.00 | 0.00 | 0.00 |
| Brevundimonas | 0.10 | 0.06 | 0.19 | 0.11 | 0.44 | 0.18 | 0.02 | 0.02 | 0.01 | 0.07 | 0.02 | 0.02 | 0.09 | 0.31 | 0.06 | 0.01 | 0.02 | 0.02 | 0.01 | 0.01 | 0.01 |
| Caulobacter | 0.03 | 0.06 | 0.00 | 0.00 | 0.10 | 0.00 | 0.02 | 0.00 | 0.00 | 0.00 | 0.02 | 0.00 | 0.03 | 0.01 | 0.01 | 0.00 | 0.00 | 0.00 | 0.00 | 0.00 | 0.00 |
| Phenylobacterium | 0.00 | 0.00 | 0.00 | 0.03 | 0.00 | 0.00 | 0.00 | 0.00 | 0.00 | 0.00 | 0.00 | 0.00 | 0.00 | 0.00 | 0.00 | 0.00 | 0.00 | 0.00 | 0.00 | 0.00 | 0.00 |
| unclassified_Hyphomonadaceae | 0.06 | 0.05 | 0.00 | 0.08 | 0.04 | 0.15 | 0.01 | 0.02 | 0.01 | 0.00 | 0.00 | 0.01 | 0.01 | 0.00 | 0.03 | 0.00 | 0.01 | 0.02 | 0.01 | 0.02 | 0.00 |
| Algimonas | 0.06 | 0.17 | 0.05 | 0.00 | 0.01 | 0.05 | 0.00 | 0.01 | 0.00 | 0.00 | 0.01 | 0.02 | 0.03 | 0.06 | 0.08 | 0.01 | 0.02 | 0.01 | 0.01 | 0.00 | 0.01 |
| Hellea | 0.09 | 0.00 | 0.01 | 0.00 | 0.09 | 0.00 | 0.00 | 0.08 | 0.05 | 0.03 | 0.08 | 0.07 | 0.07 | 0.06 | 0.10 | 0.00 | 0.00 | 0.01 | 0.01 | 0.01 | 0.00 |
| Henriciella | 0.00 | 0.00 | 0.00 | 0.00 | 0.04 | 0.00 | 0.00 | 0.00 | 0.00 | 0.00 | 0.00 | 0.00 | 0.00 | 0.00 | 0.00 | 0.00 | 0.00 | 0.00 | 0.00 | 0.00 | 0.00 |
| Hyphobacterium | 0.00 | 0.00 | 0.02 | 0.00 | 0.00 | 0.00 | 0.00 | 0.00 | 0.00 | 0.00 | 0.00 | 0.00 | 0.00 | 0.00 | 0.00 | 0.00 | 0.00 | 0.00 | 0.00 | 0.00 | 0.00 |
| Hyphomonas | 0.02 | 0.00 | 0.01 | 0.02 | 0.09 | 0.07 | 0.03 | 0.02 | 0.01 | 0.04 | 0.00 | 0.02 | 0.01 | 0.06 | 0.07 | 0.00 | 0.00 | 0.01 | 0.01 | 0.01 | 0.00 |
| Litorimonas | 0.02 | 0.10 | 0.00 | 0.00 | 0.00 | 0.00 | 0.00 | 0.00 | 0.00 | 0.09 | 0.03 | 0.02 | 0.00 | 0.01 | 0.00 | 0.00 | 0.01 | 0.01 | 0.01 | 0.01 | 0.00 |
| Maricaulis | 0.00 | 0.00 | 0.00 | 0.00 | 0.00 | 0.00 | 0.00 | 0.00 | 0.00 | 0.00 | 0.01 | 0.00 | 0.00 | 0.00 | 0.00 | 0.00 | 0.00 | 0.00 | 0.00 | 0.00 | 0.00 |
| Oceanicaulis | 0.00 | 0.00 | 0.00 | 0.00 | 0.00 | 0.05 | 0.00 | 0.00 | 0.00 | 0.00 | 0.00 | 0.00 | 0.00 | 0.00 | 0.00 | 0.00 | 0.00 | 0.00 | 0.00 | 0.00 | 0.00 |
| Robiginitomaculum | 1.15 | 0.82 | 0.45 | 0.36 | 0.32 | 1.87 | 0.79 | 1.56 | 0.31 | 0.28 | 0.42 | 0.42 | 1.18 | 0.70 | 1.94 | 0.01 | 0.04 | 0.05 | 0.04 | 0.03 | 0.02 |
| unclassified_Parvularculaceae | 0.00 | 0.01 | 0.00 | 0.00 | 0.00 | 0.00 | 0.00 | 0.01 | 0.00 | 0.00 | 0.00 | 0.00 | 0.00 | 0.00 | 0.01 | 0.00 | 0.00 | 0.00 | 0.00 | 0.00 | 0.00 |
| Parvularcula | 0.00 | 0.00 | 0.00 | 0.00 | 0.00 | 0.03 | 0.00 | 0.01 | 0.00 | 0.00 | 0.00 | 0.00 | 0.00 | 0.00 | 0.00 | 0.00 | 0.00 | 0.00 | 0.00 | 0.00 | 0.00 |
| unclassified_Elsterales | 0.00 | 0.00 | 0.00 | 0.00 | 0.00 | 0.00 | 0.00 | 0.00 | 0.00 | 0.00 | 0.00 | 0.00 | 0.00 | 0.00 | 0.00 | 0.00 | 0.00 | 0.00 | 0.00 | 0.00 | 0.00 |
| unclassified_Holosporaceae | 0.02 | 0.00 | 0.05 | 0.00 | 0.00 | 0.03 | 0.00 | 0.00 | 0.00 | 0.00 | 0.00 | 0.00 | 0.00 | 0.00 | 0.00 | 0.00 | 0.00 | 0.00 | 0.00 | 0.00 | 0.00 |
| Candidatus_Paraholospora | 0.00 | 0.00 | 0.00 | 0.00 | 0.00 | 0.00 | 0.00 | 0.00 | 0.02 | 0.00 | 0.00 | 0.00 | 0.00 | 0.00 | 0.00 | 0.00 | 0.00 | 0.00 | 0.00 | 0.00 | 0.00 |
| Holospora | 0.00 | 0.00 | 0.00 | 0.00 | 0.00 | 0.00 | 0.02 | 0.01 | 0.01 | 0.00 | 0.00 | 0.00 | 0.00 | 0.00 | 0.00 | 0.00 | 0.00 | 0.00 | 0.00 | 0.00 | 0.00 |
| unclassified_Kordiimonadales | 0.10 | 0.03 | 0.01 | 0.01 | 0.06 | 0.00 | 0.05 | 0.18 | 0.06 | 0.00 | 0.11 | 0.12 | 0.08 | 0.01 | 0.08 | 0.01 | 0.04 | 0.06 | 0.01 | 0.02 | 0.02 |
| bacterium_endosymbiont_of_Osedax_mucofloris | 0.04 | 0.05 | 0.03 | 0.00 | 0.00 | 0.07 | 0.00 | 0.00 | 0.00 | 0.00 | 0.02 | 0.01 | 0.01 | 0.00 | 0.00 | 0.00 | 0.00 | 0.00 | 0.00 | 0.00 | 0.00 |
| Kordiimonas | 0.00 | 0.02 | 0.00 | 0.00 | 0.00 | 0.06 | 0.00 | 0.00 | 0.00 | 0.00 | 0.00 | 0.00 | 0.00 | 0.00 | 0.00 | 0.00 | 0.00 | 0.00 | 0.00 | 0.00 | 0.00 |
| unclassified_Micavibrionales | 0.34 | 0.28 | 0.51 | 0.01 | 0.02 | 0.06 | 0.02 | 0.07 | 0.11 | 0.07 | 0.16 | 0.14 | 0.16 | 0.02 | 0.10 | 0.02 | 0.05 | 0.07 | 0.02 | 0.02 | 0.02 |
| unclassified_Micavibrionaceae | 0.57 | 0.22 | 0.36 | 0.14 | 0.30 | 0.23 | 0.17 | 1.19 | 0.39 | 0.25 | 0.41 | 0.45 | 0.35 | 0.31 | 0.47 | 0.02 | 0.13 | 0.17 | 0.13 | 0.06 | 0.04 |
| unclassified_NRL2 | 0.00 | 0.00 | 0.00 | 0.00 | 0.00 | 0.00 | 0.00 | 0.00 | 0.00 | 0.06 | 0.04 | 0.01 | 0.01 | 0.00 | 0.00 | 0.00 | 0.00 | 0.01 | 0.00 | 0.00 | 0.00 |
| unclassified_Paracaedibacteraceae | 0.13 | 0.19 | 0.16 | 0.08 | 0.11 | 0.00 | 0.07 | 0.22 | 0.24 | 0.00 | 0.00 | 0.00 | 0.05 | 0.00 | 0.07 | 0.01 | 0.01 | 0.00 | 0.00 | 0.01 | 0.00 |
| Candidatus_Captivus | 0.00 | 0.00 | 0.00 | 0.00 | 0.00 | 0.00 | 0.00 | 0.00 | 0.00 | 0.00 | 0.00 | 0.01 | 0.00 | 0.00 | 0.00 | 0.00 | 0.00 | 0.00 | 0.00 | 0.00 | 0.00 |
| unclassified_Parvibaculales | 0.00 | 0.00 | 0.01 | 0.00 | 0.00 | 0.01 | 0.00 | 0.01 | 0.12 | 0.00 | 0.00 | 0.00 | 0.00 | 0.00 | 0.03 | 0.00 | 0.00 | 0.01 | 0.00 | 0.00 | 0.00 |
| unclassified_PS1_clade | 0.22 | 0.42 | 0.22 | 0.34 | 0.16 | 0.46 | 0.10 | 0.21 | 0.18 | 0.14 | 0.21 | 0.23 | 0.21 | 0.26 | 0.43 | 0.06 | 0.11 | 0.14 | 0.08 | 0.11 | 0.06 |
| unclassified_Parvibaculaceae | 0.02 | 0.16 | 0.05 | 0.13 | 0.10 | 0.10 | 0.01 | 0.02 | 0.02 | 0.00 | 0.05 | 0.01 | 0.09 | 0.01 | 0.05 | 0.00 | 0.00 | 0.00 | 0.00 | 0.00 | 0.00 |
| unclassified_Puniceispirillales | 0.00 | 0.00 | 0.00 | 0.00 | 0.00 | 0.00 | 0.00 | 0.00 | 0.01 | 0.00 | 0.01 | 0.01 | 0.00 | 0.00 | 0.00 | 0.00 | 0.00 | 0.00 | 0.00 | 0.00 | 0.00 |
| unclassified_Beijerinckiaceae | 0.00 | 0.00 | 0.07 | 0.00 | 0.00 | 0.00 | 0.00 | 0.00 | 0.00 | 0.00 | 0.00 | 0.00 | 0.00 | 0.00 | 0.00 | 0.00 | 0.00 | 0.00 | 0.00 | 0.00 | 0.00 |
| Bosea | 0.07 | 0.06 | 0.10 | 0.04 | 0.06 | 0.00 | 0.01 | 0.01 | 0.00 | 0.02 | 0.02 | 0.01 | 0.03 | 0.01 | 0.01 | 0.00 | 0.00 | 0.00 | 0.00 | 0.00 | 0.00 |
| Methylobacterium | 0.05 | 0.09 | 0.11 | 0.26 | 0.25 | 0.03 | 0.01 | 0.02 | 0.01 | 0.11 | 0.01 | 0.01 | 0.03 | 0.04 | 0.05 | 0.00 | 0.00 | 0.00 | 0.01 | 0.00 | 0.00 |
| Psychroglaciecola | 0.00 | 0.00 | 0.08 | 0.00 | 0.00 | 0.00 | 0.00 | 0.00 | 0.00 | 0.00 | 0.00 | 0.00 | 0.00 | 0.00 | 0.00 | 0.00 | 0.00 | 0.00 | 0.00 | 0.00 | 0.00 |
| Roseiarcus | 0.00 | 0.00 | 0.00 | 0.00 | 0.00 | 0.00 | 0.00 | 0.00 | 0.00 | 0.00 | 0.00 | 0.00 | 0.00 | 0.00 | 0.00 | 0.00 | 0.00 | 0.00 | 0.00 | 0.00 | 0.00 |
| unclassified_Devosiaceae | 0.07 | 0.37 | 0.17 | 0.24 | 0.09 | 0.44 | 0.11 | 0.20 | 0.05 | 0.02 | 0.08 | 0.04 | 0.13 | 0.15 | 0.14 | 0.00 | 0.01 | 0.01 | 0.01 | 0.01 | 0.00 |
| Cucumibacter | 0.00 | 0.07 | 0.00 | 0.00 | 0.00 | 0.00 | 0.00 | 0.00 | 0.00 | 0.00 | 0.00 | 0.00 | 0.00 | 0.00 | 0.00 | 0.00 | 0.00 | 0.00 | 0.00 | 0.00 | 0.00 |
| Devosia | 0.03 | 0.00 | 0.02 | 0.04 | 0.00 | 0.08 | 0.02 | 0.00 | 0.00 | 0.00 | 0.00 | 0.00 | 0.00 | 0.00 | 0.01 | 0.00 | 0.00 | 0.00 | 0.00 | 0.00 | 0.00 |
| Maritalea | 0.10 | 0.31 | 0.04 | 0.87 | 0.09 | 0.97 | 0.07 | 0.32 | 0.15 | 0.19 | 0.21 | 0.12 | 0.30 | 0.04 | 0.22 | 0.02 | 0.07 | 0.16 | 0.10 | 0.04 | 0.02 |
| Filomicrobium | 0.00 | 0.00 | 0.00 | 0.00 | 0.00 | 0.02 | 0.02 | 0.04 | 0.00 | 0.00 | 0.00 | 0.01 | 0.05 | 0.00 | 0.01 | 0.00 | 0.00 | 0.01 | 0.00 | 0.01 | 0.00 |
| Hyphomicrobium | 0.00 | 0.00 | 0.00 | 0.00 | 0.00 | 0.00 | 0.00 | 0.00 | 0.00 | 0.00 | 0.00 | 0.00 | 0.00 | 0.00 | 0.01 | 0.00 | 0.00 | 0.00 | 0.00 | 0.00 | 0.00 |
| unclassified_Methyloligellaceae | 0.05 | 0.00 | 0.00 | 0.00 | 0.00 | 0.00 | 0.00 | 0.00 | 0.00 | 0.00 | 0.00 | 0.00 | 0.00 | 0.00 | 0.00 | 0.00 | 0.00 | 0.00 | 0.00 | 0.00 | 0.00 |
| unclassified_Rhizobiaceae | 0.28 | 0.43 | 0.17 | 0.05 | 0.03 | 0.19 | 0.11 | 0.24 | 0.13 | 0.18 | 0.11 | 0.10 | 0.46 | 0.13 | 0.32 | 0.01 | 0.06 | 0.09 | 0.05 | 0.04 | 0.02 |
| Ahrensia | 0.11 | 0.46 | 0.10 | 0.29 | 0.38 | 0.06 | 0.00 | 0.72 | 0.09 | 0.00 | 0.13 | 0.04 | 0.00 | 0.01 | 0.01 | 0.00 | 0.02 | 0.03 | 0.02 | 0.01 | 0.01 |
| Allorhizobium-Neorhizobium-Pararhizobium-Rhizobium | 0.05 | 0.05 | 0.13 | 0.00 | 0.07 | 0.02 | 0.00 | 0.01 | 0.00 | 0.02 | 0.01 | 0.01 | 0.01 | 0.04 | 0.00 | 0.01 | 0.00 | 0.01 | 0.01 | 0.00 | 0.01 |
| Aureimonas | 0.00 | 0.00 | 0.00 | 0.00 | 0.00 | 0.00 | 0.00 | 0.00 | 0.00 | 0.00 | 0.00 | 0.00 | 0.01 | 0.00 | 0.00 | 0.00 | 0.00 | 0.00 | 0.00 | 0.00 | 0.00 |
| Cohaesibacter | 0.64 | 0.48 | 0.54 | 0.39 | 0.44 | 0.57 | 0.10 | 0.33 | 0.15 | 0.52 | 0.97 | 0.90 | 0.14 | 0.06 | 0.16 | 0.05 | 0.61 | 1.33 | 0.41 | 0.28 | 0.21 |
| Ensifer | 0.05 | 0.22 | 0.09 | 0.12 | 0.12 | 0.03 | 0.01 | 0.00 | 0.00 | 0.00 | 0.02 | 0.00 | 0.01 | 0.07 | 0.00 | 0.00 | 0.00 | 0.01 | 0.01 | 0.00 | 0.00 |
| Hoeflea | 1.97 | 2.45 | 1.37 | 0.34 | 0.29 | 1.47 | 0.37 | 0.51 | 0.36 | 0.24 | 0.30 | 0.54 | 0.23 | 0.37 | 0.48 | 0.02 | 0.07 | 0.11 | 0.09 | 0.05 | 0.02 |
| Mesorhizobium | 0.02 | 0.00 | 0.00 | 0.00 | 0.00 | 0.05 | 0.01 | 0.02 | 0.00 | 0.00 | 0.02 | 0.02 | 0.00 | 0.00 | 0.00 | 0.00 | 0.00 | 0.00 | 0.01 | 0.00 | 0.00 |
| unclassified_Rhizobiales_Incertae_Sedis | 0.00 | 0.00 | 0.00 | 0.00 | 0.00 | 0.00 | 0.00 | 0.00 | 0.01 | 0.00 | 0.00 | 0.00 | 0.00 | 0.00 | 0.00 | 0.00 | 0.00 | 0.00 | 0.00 | 0.00 | 0.00 |
| Anderseniella | 0.01 | 0.00 | 0.00 | 0.00 | 0.00 | 0.02 | 0.00 | 0.01 | 0.02 | 0.00 | 0.03 | 0.00 | 0.00 | 0.00 | 0.00 | 0.00 | 0.00 | 0.01 | 0.00 | 0.00 | 0.00 |
| Bauldia | 0.03 | 0.00 | 0.00 | 0.00 | 0.00 | 0.00 | 0.01 | 0.00 | 0.00 | 0.00 | 0.00 | 0.01 | 0.00 | 0.00 | 0.00 | 0.00 | 0.00 | 0.00 | 0.00 | 0.00 | 0.00 |
| Nordella | 0.00 | 0.00 | 0.00 | 0.00 | 0.00 | 0.00 | 0.00 | 0.00 | 0.00 | 0.00 | 0.00 | 0.00 | 0.00 | 0.00 | 0.00 | 0.00 | 0.00 | 0.00 | 0.00 | 0.00 | 0.00 |
| Phreatobacter | 0.00 | 0.00 | 0.00 | 0.00 | 0.00 | 0.06 | 0.00 | 0.01 | 0.00 | 0.00 | 0.00 | 0.00 | 0.00 | 0.00 | 0.00 | 0.00 | 0.00 | 0.00 | 0.00 | 0.00 | 0.00 |
| Rhodomicrobium | 0.00 | 0.00 | 0.00 | 0.00 | 0.00 | 0.00 | 0.00 | 0.00 | 0.00 | 0.00 | 0.00 | 0.00 | 0.00 | 0.00 | 0.00 | 0.00 | 0.00 | 0.00 | 0.00 | 0.00 | 0.00 |
| Labrenzia | 0.09 | 0.04 | 0.08 | 0.06 | 0.23 | 0.53 | 0.06 | 0.11 | 0.02 | 0.18 | 0.33 | 0.17 | 0.09 | 0.00 | 0.05 | 0.01 | 0.07 | 0.13 | 0.16 | 0.10 | 0.05 |
| Pseudovibrio | 0.05 | 0.08 | 0.02 | 0.03 | 0.00 | 0.19 | 0.00 | 0.01 | 0.00 | 0.00 | 0.01 | 0.02 | 0.16 | 0.03 | 0.12 | 0.00 | 0.00 | 0.01 | 0.01 | 0.00 | 0.00 |
| unclassified_Xanthobacteraceae | 0.00 | 0.00 | 0.00 | 0.00 | 0.00 | 0.00 | 0.04 | 0.00 | 0.00 | 0.00 | 0.00 | 0.00 | 0.00 | 0.00 | 0.00 | 0.00 | 0.00 | 0.00 | 0.00 | 0.00 | 0.00 |
| Bradyrhizobium | 0.01 | 0.04 | 0.05 | 0.00 | 0.00 | 0.00 | 0.00 | 0.00 | 0.00 | 0.00 | 0.00 | 0.00 | 0.00 | 0.00 | 0.00 | 0.00 | 0.00 | 0.00 | 0.00 | 0.00 | 0.00 |
| Pseudolabrys | 0.00 | 0.00 | 0.00 | 0.00 | 0.00 | 0.00 | 0.01 | 0.00 | 0.00 | 0.00 | 0.00 | 0.00 | 0.00 | 0.00 | 0.00 | 0.00 | 0.00 | 0.00 | 0.00 | 0.00 | 0.00 |
| unclassified_Rhodobacteraceae | 0.01 | 0.25 | 0.00 | 0.02 | 0.04 | 0.00 | 0.00 | 0.03 | 0.00 | 0.00 | 0.00 | 0.01 | 0.03 | 0.00 | 0.01 | 0.00 | 0.01 | 0.01 | 0.01 | 0.00 | 0.00 |
| Actibacterium | 0.02 | 0.00 | 0.00 | 0.00 | 0.00 | 0.03 | 0.01 | 0.04 | 0.04 | 0.00 | 0.00 | 0.01 | 0.00 | 0.00 | 0.01 | 0.00 | 0.00 | 0.01 | 0.00 | 0.00 | 0.00 |
| Albirhodobacter | 1.51 | 0.99 | 0.55 | 0.44 | 0.18 | 0.96 | 0.06 | 0.15 | 0.14 | 0.03 | 0.13 | 0.19 | 0.09 | 0.01 | 0.05 | 0.02 | 0.03 | 0.05 | 0.04 | 0.03 | 0.02 |
| Aliiroseovarius | 0.05 | 0.02 | 0.03 | 0.03 | 0.01 | 0.01 | 0.01 | 0.05 | 0.03 | 0.01 | 0.02 | 0.02 | 0.01 | 0.01 | 0.01 | 0.01 | 0.01 | 0.02 | 0.01 | 0.02 | 0.00 |
| Amaricoccus | 0.06 | 0.00 | 0.00 | 0.00 | 0.00 | 0.00 | 0.00 | 0.00 | 0.00 | 0.00 | 0.00 | 0.00 | 0.00 | 0.00 | 0.00 | 0.00 | 0.00 | 0.00 | 0.00 | 0.00 | 0.00 |
| Celeribacter | 0.02 | 0.00 | 0.15 | 0.00 | 0.01 | 0.05 | 0.01 | 0.04 | 0.07 | 0.00 | 0.02 | 0.01 | 0.02 | 0.00 | 0.01 | 0.00 | 0.02 | 0.05 | 0.03 | 0.01 | 0.01 |
| Donghicola | 0.91 | 1.11 | 0.32 | 0.19 | 0.31 | 1.27 | 0.53 | 0.60 | 0.93 | 0.22 | 0.33 | 0.35 | 0.54 | 0.45 | 0.26 | 0.13 | 0.31 | 0.64 | 0.25 | 0.23 | 0.11 |
| Jannaschia | 0.03 | 0.14 | 0.00 | 0.00 | 0.00 | 0.08 | 0.00 | 0.00 | 0.01 | 0.00 | 0.06 | 0.01 | 0.00 | 0.00 | 0.01 | 0.00 | 0.00 | 0.02 | 0.01 | 0.00 | 0.00 |
| Litoreibacter | 0.82 | 1.38 | 0.37 | 0.04 | 0.14 | 0.50 | 0.09 | 0.18 | 0.30 | 0.05 | 0.30 | 0.15 | 0.21 | 0.07 | 0.10 | 0.01 | 0.05 | 0.14 | 0.21 | 0.06 | 0.03 |
| Loktanella | 0.57 | 0.67 | 0.31 | 0.30 | 0.14 | 0.32 | 0.04 | 0.47 | 0.10 | 0.06 | 0.07 | 0.06 | 0.20 | 0.02 | 0.12 | 0.02 | 0.03 | 0.05 | 0.04 | 0.05 | 0.02 |
| Marivita | 0.08 | 0.22 | 0.01 | 0.01 | 0.01 | 0.01 | 0.02 | 0.03 | 0.05 | 0.13 | 0.09 | 0.17 | 0.03 | 0.02 | 0.01 | 0.01 | 0.21 | 0.48 | 0.25 | 0.07 | 0.03 |
| Nautella | 1.23 | 0.74 | 0.56 | 0.30 | 0.21 | 0.48 | 0.37 | 1.20 | 0.83 | 0.39 | 0.61 | 0.85 | 1.07 | 0.36 | 0.61 | 0.73 | 1.08 | 2.24 | 4.54 | 7.14 | 1.19 |
| Oceanicella | 0.00 | 0.00 | 0.00 | 0.00 | 0.00 | 0.00 | 0.02 | 0.00 | 0.00 | 0.00 | 0.00 | 0.00 | 0.00 | 0.00 | 0.00 | 0.00 | 0.00 | 0.00 | 0.00 | 0.00 | 0.00 |
| Oceaniovalibus | 0.02 | 0.01 | 0.01 | 0.01 | 0.02 | 0.01 | 0.00 | 0.02 | 0.01 | 0.00 | 0.01 | 0.04 | 0.01 | 0.00 | 0.01 | 0.01 | 0.02 | 0.05 | 0.01 | 0.03 | 0.01 |
| Paracoccus | 0.02 | 0.00 | 0.06 | 0.04 | 0.07 | 0.01 | 0.01 | 0.00 | 0.01 | 0.00 | 0.00 | 0.00 | 0.00 | 0.03 | 0.00 | 0.00 | 0.00 | 0.00 | 0.00 | 0.00 | 0.00 |
| Phaeobacter | 0.01 | 0.00 | 0.00 | 0.01 | 0.01 | 0.00 | 0.01 | 0.00 | 0.00 | 0.02 | 0.00 | 0.00 | 0.00 | 0.01 | 0.00 | 0.00 | 0.10 | 0.08 | 0.00 | 0.01 | 0.01 |
| Planktotalea | 1.76 | 0.84 | 0.50 | 0.09 | 0.08 | 0.99 | 0.29 | 1.04 | 0.87 | 0.03 | 0.28 | 0.20 | 1.80 | 0.44 | 0.78 | 0.02 | 0.07 | 0.16 | 0.10 | 0.07 | 0.02 |
| Pontivivens | 0.03 | 0.03 | 0.03 | 0.02 | 0.02 | 0.02 | 0.02 | 0.05 | 0.04 | 0.03 | 0.04 | 0.05 | 0.02 | 0.04 | 0.02 | 0.03 | 0.04 | 0.05 | 0.03 | 0.03 | 0.02 |
| Roseobacter_clade_NAC11-7_lineage | 0.00 | 0.00 | 0.16 | 0.00 | 0.00 | 0.00 | 0.00 | 0.02 | 0.01 | 0.00 | 0.00 | 0.00 | 0.00 | 0.00 | 0.00 | 0.00 | 0.00 | 0.09 | 0.01 | 0.01 | 0.00 |
| Rubellimicrobium | 0.00 | 0.00 | 0.00 | 0.00 | 0.00 | 0.00 | 0.00 | 0.00 | 0.00 | 0.07 | 0.00 | 0.00 | 0.00 | 0.00 | 0.00 | 0.00 | 0.00 | 0.00 | 0.00 | 0.00 | 0.00 |
| Rubribacterium | 0.00 | 0.00 | 0.00 | 0.00 | 0.00 | 0.00 | 0.02 | 0.00 | 0.00 | 0.00 | 0.00 | 0.00 | 0.00 | 0.00 | 0.00 | 0.00 | 0.00 | 0.00 | 0.00 | 0.00 | 0.00 |
| Ruegeria | 0.81 | 1.21 | 0.27 | 0.44 | 0.12 | 0.66 | 0.09 | 0.47 | 0.58 | 0.02 | 0.23 | 0.24 | 0.29 | 0.21 | 0.20 | 0.05 | 0.06 | 0.10 | 0.19 | 0.10 | 0.03 |
| Sedimentitalea | 0.96 | 0.84 | 0.59 | 0.17 | 0.11 | 0.30 | 0.90 | 4.33 | 3.02 | 0.19 | 0.65 | 0.58 | 1.26 | 0.44 | 0.74 | 0.11 | 0.64 | 0.95 | 0.27 | 0.32 | 0.07 |
| Silicimonas | 0.00 | 0.00 | 0.09 | 0.00 | 0.00 | 0.00 | 0.00 | 0.00 | 0.03 | 0.00 | 0.00 | 0.00 | 0.00 | 0.00 | 0.00 | 0.00 | 0.00 | 0.01 | 0.00 | 0.00 | 0.00 |
| Sulfitobacter | 1.49 | 1.23 | 0.34 | 0.13 | 0.13 | 0.52 | 0.18 | 0.36 | 0.60 | 0.08 | 0.26 | 0.27 | 0.35 | 0.21 | 0.15 | 0.07 | 0.12 | 0.28 | 0.16 | 0.14 | 0.04 |
| Thalassobius | 0.23 | 0.13 | 0.10 | 0.01 | 0.05 | 0.12 | 0.04 | 0.25 | 0.24 | 0.00 | 0.14 | 0.05 | 0.06 | 0.02 | 0.05 | 0.02 | 0.00 | 0.05 | 0.04 | 0.05 | 0.01 |
| unclassified_Rhodospirillales | 0.17 | 0.17 | 0.04 | 0.58 | 0.12 | 0.14 | 0.01 | 0.01 | 0.00 | 0.01 | 0.00 | 0.01 | 0.04 | 0.01 | 0.05 | 0.04 | 0.06 | 0.05 | 1.08 | 0.46 | 0.08 |
| Azospirillum_sp.47_25 | 0.00 | 0.00 | 0.00 | 0.00 | 0.00 | 0.00 | 0.01 | 0.00 | 0.00 | 0.00 | 0.00 | 0.00 | 0.00 | 0.00 | 0.00 | 0.00 | 0.00 | 0.00 | 0.00 | 0.00 | 0.00 |
| unclassified_Magnetospiraceae | 0.02 | 0.01 | 0.00 | 0.02 | 0.02 | 0.22 | 0.00 | 0.03 | 0.03 | 0.00 | 0.11 | 0.07 | 0.03 | 0.00 | 0.01 | 0.01 | 0.00 | 0.01 | 0.00 | 0.01 | 0.00 |
| Magnetospira | 0.00 | 0.00 | 0.00 | 0.00 | 0.00 | 0.02 | 0.00 | 0.00 | 0.00 | 0.00 | 0.00 | 0.00 | 0.00 | 0.00 | 0.01 | 0.00 | 0.00 | 0.00 | 0.00 | 0.00 | 0.00 |
| unclassified_Magnetospirillaceae | 0.00 | 0.02 | 0.00 | 0.00 | 0.01 | 0.00 | 0.00 | 0.00 | 0.00 | 0.00 | 0.01 | 0.00 | 0.00 | 0.01 | 0.01 | 0.00 | 0.00 | 0.00 | 0.00 | 0.00 | 0.00 |
| Defluviicoccus | 0.00 | 0.00 | 0.00 | 0.00 | 0.00 | 0.00 | 0.03 | 0.00 | 0.00 | 0.00 | 0.00 | 0.00 | 0.00 | 0.00 | 0.00 | 0.00 | 0.00 | 0.00 | 0.00 | 0.00 | 0.00 |
| Candidatus_Riegeria | 0.00 | 0.00 | 0.00 | 0.00 | 0.00 | 0.00 | 0.00 | 0.00 | 0.00 | 0.00 | 0.00 | 0.00 | 0.00 | 0.00 | 0.00 | 0.01 | 0.00 | 0.00 | 0.02 | 0.01 | 0.00 |
| unclassified_Terasakiellaceae | 0.14 | 0.23 | 0.04 | 0.04 | 0.06 | 0.04 | 0.03 | 0.02 | 0.03 | 4.77 | 4.59 | 5.17 | 0.04 | 0.12 | 0.08 | 0.15 | 2.37 | 2.24 | 1.18 | 1.94 | 0.71 |
| Aestuariispira | 0.00 | 0.00 | 0.00 | 0.00 | 0.03 | 0.00 | 0.00 | 0.00 | 0.00 | 0.06 | 0.26 | 0.30 | 0.00 | 0.00 | 0.00 | 0.01 | 0.03 | 0.04 | 0.03 | 0.03 | 0.03 |
| Terasakiella | 0.02 | 0.01 | 0.02 | 0.12 | 0.01 | 0.08 | 0.01 | 0.01 | 0.01 | 0.18 | 1.92 | 1.78 | 0.05 | 0.01 | 0.02 | 0.14 | 1.88 | 1.04 | 0.16 | 0.10 | 0.41 |
| Thalassospira | 0.02 | 0.06 | 0.00 | 0.00 | 0.00 | 0.00 | 0.00 | 0.00 | 0.00 | 0.00 | 0.00 | 0.00 | 0.00 | 0.00 | 0.00 | 0.00 | 0.00 | 0.00 | 0.00 | 0.00 | 0.00 |
| unclassified_Kiloniellaceae | 0.00 | 0.05 | 0.00 | 0.00 | 0.00 | 0.00 | 0.00 | 0.01 | 0.00 | 0.00 | 0.02 | 0.01 | 0.00 | 0.00 | 0.00 | 0.00 | 0.00 | 0.00 | 0.00 | 0.00 | 0.00 |
| Kiloniella | 0.03 | 0.00 | 0.00 | 0.02 | 0.00 | 0.04 | 0.01 | 0.02 | 0.01 | 0.51 | 0.62 | 0.85 | 0.15 | 0.00 | 0.05 | 0.01 | 0.10 | 0.15 | 0.08 | 0.10 | 0.05 |
| Limibacillus | 0.00 | 0.00 | 0.00 | 0.00 | 0.00 | 0.00 | 0.03 | 0.00 | 0.00 | 0.00 | 0.00 | 0.00 | 0.00 | 0.00 | 0.00 | 0.00 | 0.00 | 0.00 | 0.00 | 0.00 | 0.00 |
| unclassified_Rickettsiales | 0.00 | 0.00 | 0.00 | 0.00 | 0.00 | 0.12 | 0.06 | 0.01 | 0.01 | 0.00 | 0.01 | 0.00 | 0.01 | 0.00 | 0.06 | 0.00 | 0.00 | 0.00 | 0.00 | 0.00 | 0.00 |
| unclassified_AB1 | 0.25 | 0.15 | 0.08 | 0.06 | 0.12 | 0.45 | 0.02 | 0.05 | 0.03 | 0.03 | 0.07 | 0.05 | 0.07 | 0.08 | 0.07 | 0.01 | 0.02 | 0.01 | 0.02 | 0.01 | 0.01 |
| Sinorickettsia_chlamys | 0.41 | 0.29 | 0.24 | 0.09 | 0.02 | 0.17 | 0.14 | 0.34 | 0.10 | 0.07 | 0.08 | 0.07 | 0.54 | 0.37 | 0.64 | 0.01 | 0.03 | 0.03 | 0.07 | 0.02 | 0.01 |
| Candidatus_Neoehrlichia | 0.00 | 0.00 | 0.00 | 0.00 | 0.00 | 0.00 | 0.00 | 0.00 | 0.00 | 0.00 | 0.00 | 0.00 | 0.00 | 0.00 | 0.00 | 0.00 | 0.00 | 0.00 | 0.00 | 0.00 | 0.00 |
| unclassified_Midichloriaceae | 0.00 | 0.00 | 0.00 | 0.00 | 0.00 | 0.00 | 0.00 | 0.02 | 0.00 | 0.00 | 0.00 | 0.00 | 0.00 | 0.00 | 0.01 | 0.00 | 0.00 | 0.00 | 0.00 | 0.00 | 0.00 |
| Candidatus_Anadelfobacter | 0.00 | 0.00 | 0.00 | 0.00 | 0.00 | 0.00 | 0.00 | 0.00 | 0.01 | 0.00 | 0.00 | 0.00 | 0.00 | 0.00 | 0.00 | 0.00 | 0.00 | 0.00 | 0.00 | 0.00 | 0.00 |
| Candidatus_Jidaibacter | 0.03 | 0.00 | 0.00 | 0.00 | 0.00 | 0.00 | 0.00 | 0.00 | 0.01 | 0.00 | 0.00 | 0.03 | 0.00 | 0.00 | 0.00 | 0.00 | 0.00 | 0.00 | 0.00 | 0.00 | 0.00 |
| unclassified_Rickettsiaceae | 0.50 | 0.46 | 0.15 | 0.00 | 0.00 | 0.13 | 0.04 | 0.01 | 0.03 | 0.15 | 0.21 | 0.14 | 0.04 | 0.10 | 0.25 | 0.00 | 0.01 | 0.02 | 0.01 | 0.00 | 0.00 |
| Candidatus_Megaira | 0.02 | 0.00 | 0.05 | 0.00 | 0.00 | 0.01 | 0.00 | 0.02 | 0.00 | 0.00 | 0.00 | 0.00 | 0.02 | 0.05 | 0.01 | 0.00 | 0.00 | 0.00 | 0.00 | 0.00 | 0.00 |
| unclassified_SM2D12 | 0.31 | 0.14 | 0.28 | 0.09 | 0.11 | 0.15 | 0.05 | 0.12 | 0.06 | 0.04 | 0.05 | 0.07 | 0.54 | 0.26 | 0.73 | 0.01 | 0.02 | 0.02 | 0.02 | 0.01 | 0.00 |
| marine_metagenome | 0.01 | 0.00 | 0.00 | 0.00 | 0.00 | 0.00 | 0.00 | 0.00 | 0.00 | 0.00 | 0.00 | 0.00 | 0.00 | 0.00 | 0.00 | 0.00 | 0.00 | 0.00 | 0.00 | 0.00 | 0.00 |
| Sneathiella | 0.00 | 0.00 | 0.00 | 0.00 | 0.00 | 0.00 | 0.00 | 0.00 | 0.00 | 0.02 | 0.00 | 0.02 | 0.00 | 0.00 | 0.00 | 0.00 | 0.00 | 0.01 | 0.00 | 0.00 | 0.00 |
| Altererythrobacter | 0.34 | 0.66 | 0.14 | 0.25 | 0.09 | 0.29 | 0.03 | 0.38 | 0.01 | 0.01 | 0.06 | 0.11 | 0.05 | 0.02 | 0.07 | 0.01 | 0.00 | 0.01 | 0.02 | 0.00 | 0.01 |
| DSSF69 | 0.00 | 0.00 | 0.00 | 0.00 | 0.00 | 0.00 | 0.04 | 0.00 | 0.00 | 0.00 | 0.00 | 0.00 | 0.00 | 0.00 | 0.00 | 0.00 | 0.00 | 0.00 | 0.00 | 0.00 | 0.00 |
| Erythrobacter | 0.12 | 0.05 | 0.12 | 0.14 | 0.01 | 0.07 | 0.05 | 0.02 | 0.02 | 0.00 | 0.02 | 0.00 | 0.03 | 0.07 | 0.00 | 0.00 | 0.00 | 0.01 | 0.01 | 0.00 | 0.00 |
| Parasphingopyxis | 0.00 | 0.01 | 0.00 | 0.00 | 0.00 | 0.11 | 0.00 | 0.00 | 0.01 | 0.00 | 0.00 | 0.00 | 0.00 | 0.00 | 0.00 | 0.00 | 0.00 | 0.00 | 0.00 | 0.00 | 0.00 |
| Rhizorhapis | 0.00 | 0.00 | 0.00 | 0.00 | 0.00 | 0.00 | 0.00 | 0.00 | 0.00 | 0.00 | 0.00 | 0.00 | 0.00 | 0.00 | 0.01 | 0.00 | 0.00 | 0.00 | 0.00 | 0.00 | 0.00 |
| Sphingobium | 0.00 | 0.04 | 0.00 | 0.05 | 0.00 | 0.13 | 0.00 | 0.00 | 0.00 | 0.04 | 0.00 | 0.00 | 0.00 | 0.00 | 0.00 | 0.00 | 0.00 | 0.00 | 0.00 | 0.00 | 0.00 |
| Sphingomonas | 0.04 | 0.28 | 0.28 | 0.44 | 0.11 | 0.17 | 0.05 | 0.02 | 0.01 | 0.14 | 0.02 | 0.04 | 0.18 | 0.01 | 0.03 | 0.01 | 0.02 | 0.02 | 0.02 | 0.03 | 0.01 |
| Sphingorhabdus | 0.27 | 0.67 | 0.71 | 0.33 | 0.23 | 1.14 | 0.07 | 2.78 | 0.13 | 0.19 | 0.27 | 0.15 | 0.10 | 0.07 | 0.08 | 0.02 | 0.03 | 0.04 | 0.04 | 0.04 | 0.01 |
| unclassified_Deltaproteobacteria | 0.00 | 0.00 | 0.00 | 0.00 | 0.00 | 0.00 | 0.00 | 0.00 | 0.00 | 0.00 | 0.00 | 0.00 | 0.00 | 0.00 | 0.00 | 0.00 | 0.00 | 0.00 | 0.00 | 0.00 | 0.00 |
| unclassified_Bacteriovoracaceae | 0.01 | 0.05 | 0.01 | 0.02 | 0.00 | 0.01 | 0.12 | 0.02 | 0.02 | 0.03 | 0.90 | 0.00 | 0.06 | 0.07 | 0.13 | 0.30 | 0.02 | 0.10 | 0.17 | 0.15 | 0.04 |
| Halobacteriovorax | 0.19 | 0.21 | 0.10 | 0.04 | 0.09 | 0.21 | 0.14 | 0.23 | 0.11 | 0.09 | 0.10 | 0.09 | 0.15 | 0.06 | 0.10 | 0.03 | 7.90 | 12.10 | 2.74 | 0.20 | 1.13 |
| Peredibacter | 0.99 | 0.97 | 0.39 | 0.50 | 0.13 | 0.90 | 0.14 | 0.42 | 0.23 | 0.35 | 0.38 | 0.57 | 0.25 | 0.29 | 0.23 | 0.05 | 0.08 | 0.10 | 0.71 | 1.00 | 0.15 |
| Bdellovibrio | 0.10 | 0.22 | 0.21 | 0.10 | 0.04 | 0.31 | 0.07 | 0.28 | 0.07 | 0.23 | 0.06 | 0.08 | 0.08 | 0.07 | 0.13 | 0.03 | 0.05 | 0.07 | 0.04 | 0.02 | 0.02 |
| OM27_clade | 0.08 | 0.01 | 0.00 | 0.00 | 0.00 | 0.00 | 0.09 | 0.00 | 0.01 | 0.01 | 0.00 | 0.00 | 0.00 | 0.00 | 0.02 | 0.00 | 0.00 | 0.00 | 0.00 | 0.00 | 0.00 |
| unclassified_Bradymonadales | 0.00 | 0.00 | 0.00 | 0.00 | 0.00 | 0.00 | 0.00 | 0.00 | 0.00 | 0.00 | 0.00 | 0.00 | 0.00 | 0.00 | 0.00 | 0.00 | 0.00 | 0.00 | 0.00 | 0.00 | 0.00 |
| Desulfatiglans | 0.00 | 0.00 | 0.00 | 0.00 | 0.00 | 0.00 | 0.04 | 0.00 | 0.00 | 0.00 | 0.00 | 0.00 | 0.00 | 0.00 | 0.00 | 0.00 | 0.00 | 0.00 | 0.00 | 0.00 | 0.00 |
| unclassified_Desulfobacteraceae | 0.00 | 0.00 | 0.00 | 0.00 | 0.00 | 0.00 | 0.03 | 0.00 | 0.00 | 0.00 | 0.00 | 0.00 | 0.00 | 0.00 | 0.00 | 0.00 | 0.00 | 0.00 | 0.00 | 0.00 | 0.00 |
| Desulfatitalea | 0.00 | 0.00 | 0.00 | 0.00 | 0.00 | 0.00 | 0.01 | 0.00 | 0.00 | 0.00 | 0.00 | 0.00 | 0.00 | 0.00 | 0.00 | 0.00 | 0.00 | 0.00 | 0.00 | 0.00 | 0.00 |
| Desulfobacter | 0.00 | 0.04 | 0.00 | 0.00 | 0.00 | 0.00 | 0.00 | 0.00 | 0.00 | 0.00 | 0.00 | 0.00 | 0.00 | 0.00 | 0.00 | 0.00 | 0.00 | 0.00 | 0.00 | 0.00 | 0.00 |
| Desulfococcus | 0.00 | 0.00 | 0.00 | 0.00 | 0.00 | 0.00 | 0.01 | 0.00 | 0.00 | 0.00 | 0.00 | 0.00 | 0.00 | 0.00 | 0.00 | 0.00 | 0.00 | 0.00 | 0.00 | 0.00 | 0.00 |
| SEEP-SRB1 | 0.00 | 0.00 | 0.00 | 0.00 | 0.00 | 0.00 | 0.05 | 0.00 | 0.00 | 0.00 | 0.00 | 0.00 | 0.00 | 0.00 | 0.00 | 0.00 | 0.00 | 0.00 | 0.00 | 0.00 | 0.00 |
| Sva0081_sediment_group | 0.00 | 0.00 | 0.00 | 0.00 | 0.00 | 0.00 | 0.07 | 0.00 | 0.00 | 0.00 | 0.00 | 0.00 | 0.00 | 0.00 | 0.00 | 0.00 | 0.00 | 0.00 | 0.00 | 0.00 | 0.00 |
| Desulfobulbus | 0.00 | 0.00 | 0.00 | 0.00 | 0.00 | 0.00 | 0.00 | 0.00 | 0.00 | 0.00 | 0.01 | 0.00 | 0.00 | 0.00 | 0.00 | 0.00 | 0.00 | 0.00 | 0.00 | 0.00 | 0.00 |
| Desulfopila | 0.00 | 0.03 | 0.00 | 0.00 | 0.00 | 0.00 | 0.00 | 0.00 | 0.00 | 0.00 | 0.00 | 0.00 | 0.00 | 0.00 | 0.00 | 0.00 | 0.00 | 0.00 | 0.00 | 0.00 | 0.00 |
| Desulforhopalus | 0.00 | 0.00 | 0.00 | 0.01 | 0.05 | 0.00 | 0.00 | 0.01 | 0.00 | 0.00 | 0.01 | 0.00 | 0.00 | 0.00 | 0.00 | 0.00 | 0.00 | 0.00 | 0.00 | 0.00 | 0.00 |
| Desulfotalea | 0.00 | 0.00 | 0.00 | 0.00 | 0.00 | 0.08 | 0.00 | 0.01 | 0.00 | 0.00 | 0.00 | 0.00 | 0.00 | 0.00 | 0.00 | 0.00 | 0.00 | 0.00 | 0.00 | 0.00 | 0.00 |
| Desulfurivibrio | 0.00 | 0.00 | 0.00 | 0.00 | 0.00 | 0.00 | 0.00 | 0.00 | 0.00 | 0.00 | 0.00 | 0.00 | 0.00 | 0.00 | 0.00 | 0.00 | 0.00 | 0.00 | 0.00 | 0.00 | 0.00 |
| SEEP-SRB4 | 0.00 | 0.00 | 0.00 | 0.00 | 0.04 | 0.00 | 0.00 | 0.00 | 0.00 | 0.00 | 0.00 | 0.00 | 0.00 | 0.00 | 0.00 | 0.00 | 0.00 | 0.00 | 0.00 | 0.00 | 0.00 |
| unclassified_Desulfovibrionaceae | 0.00 | 0.00 | 0.00 | 0.00 | 0.00 | 0.00 | 0.00 | 0.00 | 0.00 | 0.02 | 0.00 | 0.00 | 0.00 | 0.00 | 0.00 | 0.00 | 0.00 | 0.00 | 0.00 | 0.00 | 0.00 |
| Desulfovibrio | 0.00 | 0.00 | 0.05 | 0.16 | 0.08 | 0.03 | 0.04 | 0.01 | 0.00 | 0.00 | 0.01 | 0.00 | 0.00 | 0.01 | 0.00 | 0.04 | 0.00 | 0.00 | 0.01 | 0.01 | 0.00 |
| Halodesulfovibrio | 0.05 | 0.10 | 0.05 | 1.34 | 0.78 | 1.79 | 0.08 | 0.08 | 1.48 | 0.01 | 0.01 | 0.02 | 0.02 | 0.02 | 0.05 | 0.08 | 0.01 | 0.02 | 0.06 | 0.02 | 0.02 |
| Lawsonia | 0.00 | 0.03 | 0.00 | 0.00 | 0.00 | 0.00 | 0.00 | 0.00 | 0.00 | 0.00 | 0.00 | 0.00 | 0.00 | 0.00 | 0.00 | 0.00 | 0.00 | 0.00 | 0.00 | 0.00 | 0.00 |
| Desulfuromusa | 0.01 | 0.00 | 0.00 | 0.00 | 0.00 | 0.00 | 0.00 | 0.00 | 0.00 | 0.00 | 0.00 | 0.00 | 0.00 | 0.00 | 0.00 | 0.00 | 0.00 | 0.00 | 0.00 | 0.00 | 0.00 |
| Geobacter | 0.00 | 0.00 | 0.00 | 0.00 | 0.00 | 0.00 | 0.04 | 0.00 | 0.00 | 0.00 | 0.00 | 0.00 | 0.00 | 0.00 | 0.00 | 0.00 | 0.00 | 0.00 | 0.00 | 0.00 | 0.00 |
| unclassified_Sva1033 | 0.00 | 0.00 | 0.00 | 0.00 | 0.00 | 0.00 | 0.01 | 0.00 | 0.00 | 0.00 | 0.00 | 0.00 | 0.00 | 0.00 | 0.00 | 0.00 | 0.00 | 0.00 | 0.00 | 0.00 | 0.00 |
| unclassified_MBNT15 | 0.00 | 0.00 | 0.00 | 0.00 | 0.00 | 0.00 | 0.01 | 0.00 | 0.00 | 0.00 | 0.01 | 0.00 | 0.00 | 0.00 | 0.00 | 0.00 | 0.00 | 0.00 | 0.00 | 0.00 | 0.00 |
| Nitrospirae_bacterium_CG2_30_70_394 | 0.00 | 0.00 | 0.00 | 0.00 | 0.00 | 0.00 | 0.02 | 0.00 | 0.00 | 0.00 | 0.00 | 0.00 | 0.00 | 0.00 | 0.00 | 0.00 | 0.00 | 0.00 | 0.00 | 0.00 | 0.00 |
| unclassified_BIrii41 | 0.00 | 0.00 | 0.02 | 0.00 | 0.00 | 0.00 | 0.08 | 0.00 | 0.00 | 0.00 | 0.00 | 0.00 | 0.00 | 0.00 | 0.00 | 0.00 | 0.00 | 0.00 | 0.00 | 0.00 | 0.00 |
| Anaeromyxobacter_dehalogenans | 0.00 | 0.00 | 0.00 | 0.24 | 0.00 | 0.00 | 0.00 | 0.00 | 0.00 | 0.00 | 0.00 | 0.00 | 0.00 | 0.00 | 0.00 | 0.00 | 0.00 | 0.00 | 0.00 | 0.00 | 0.00 |
| Haliangium | 0.00 | 0.01 | 0.00 | 0.00 | 0.00 | 0.00 | 0.01 | 0.00 | 0.00 | 0.00 | 0.00 | 0.00 | 0.00 | 0.00 | 0.00 | 0.00 | 0.00 | 0.00 | 0.00 | 0.00 | 0.00 |
| unclassified_MidBa8 | 0.00 | 0.00 | 0.00 | 0.00 | 0.00 | 0.00 | 0.01 | 0.00 | 0.00 | 0.00 | 0.00 | 0.00 | 0.00 | 0.00 | 0.00 | 0.00 | 0.00 | 0.00 | 0.00 | 0.00 | 0.00 |
| unclassified_Nannocystaceae | 0.00 | 0.00 | 0.02 | 0.00 | 0.00 | 0.00 | 0.00 | 0.00 | 0.00 | 0.00 | 0.00 | 0.00 | 0.00 | 0.00 | 0.01 | 0.00 | 0.00 | 0.00 | 0.00 | 0.00 | 0.00 |
| Nannocystis | 0.01 | 0.00 | 0.01 | 0.01 | 0.01 | 0.01 | 0.01 | 0.00 | 0.00 | 0.00 | 0.01 | 0.01 | 0.01 | 0.00 | 0.00 | 0.01 | 0.01 | 0.01 | 0.01 | 0.00 | 0.01 |
| unclassified_P3OB-42 | 0.01 | 0.00 | 0.00 | 0.00 | 0.00 | 0.00 | 0.00 | 0.00 | 0.00 | 0.00 | 0.00 | 0.00 | 0.00 | 0.00 | 0.00 | 0.00 | 0.00 | 0.00 | 0.00 | 0.00 | 0.00 |
| Sorangium | 0.00 | 0.00 | 0.00 | 0.00 | 0.00 | 0.00 | 0.02 | 0.00 | 0.00 | 0.00 | 0.00 | 0.00 | 0.00 | 0.00 | 0.00 | 0.00 | 0.00 | 0.00 | 0.00 | 0.00 | 0.00 |
| unclassified_Sandaracinaceae | 0.08 | 0.01 | 0.02 | 0.00 | 0.02 | 0.07 | 0.02 | 0.02 | 0.00 | 0.00 | 0.03 | 0.01 | 0.03 | 0.00 | 0.01 | 0.00 | 0.00 | 0.01 | 0.01 | 0.01 | 0.00 |
| Sandaracinus | 0.00 | 0.00 | 0.00 | 0.00 | 0.02 | 0.00 | 0.00 | 0.00 | 0.00 | 0.00 | 0.00 | 0.00 | 0.00 | 0.00 | 0.00 | 0.00 | 0.00 | 0.00 | 0.00 | 0.00 | 0.00 |
| unclassified_bacteriap25 | 0.00 | 0.00 | 0.00 | 0.00 | 0.00 | 0.00 | 0.00 | 0.00 | 0.00 | 0.00 | 0.00 | 0.00 | 0.01 | 0.00 | 0.01 | 0.00 | 0.00 | 0.00 | 0.00 | 0.00 | 0.00 |
| Deltaproteobacteria_bacterium_GWA2_45_12 | 0.00 | 0.00 | 0.00 | 0.00 | 0.00 | 0.00 | 0.00 | 0.00 | 0.00 | 0.60 | 0.15 | 0.13 | 0.01 | 0.00 | 0.02 | 0.00 | 0.01 | 0.03 | 0.10 | 0.01 | 0.01 |
| unclassified_mle1-27 | 0.00 | 0.00 | 0.00 | 0.33 | 0.00 | 0.00 | 0.00 | 0.00 | 0.00 | 0.00 | 0.00 | 0.00 | 0.00 | 0.00 | 0.00 | 0.00 | 0.00 | 0.00 | 0.00 | 0.00 | 0.00 |
| unclassified_NB1-j | 0.00 | 0.00 | 0.00 | 0.00 | 0.00 | 0.00 | 0.04 | 0.00 | 0.00 | 0.00 | 0.00 | 0.00 | 0.00 | 0.00 | 0.00 | 0.00 | 0.00 | 0.00 | 0.00 | 0.00 | 0.00 |
| unclassified_0319-6G20 | 0.04 | 0.00 | 0.06 | 0.00 | 0.03 | 0.05 | 0.01 | 0.01 | 0.00 | 0.01 | 0.00 | 0.01 | 0.02 | 0.00 | 0.00 | 0.00 | 0.00 | 0.00 | 0.00 | 0.00 | 0.00 |
| unclassified_Oligoflexaceae | 0.02 | 0.03 | 0.00 | 0.00 | 0.00 | 0.05 | 0.00 | 0.00 | 0.00 | 0.00 | 0.00 | 0.00 | 0.00 | 0.00 | 0.01 | 0.00 | 0.00 | 0.00 | 0.00 | 0.00 | 0.00 |
| unclassified_PB19 | 0.01 | 0.03 | 0.01 | 0.01 | 0.00 | 0.11 | 0.01 | 0.01 | 0.07 | 0.02 | 0.01 | 0.05 | 0.00 | 0.05 | 0.01 | 0.03 | 0.01 | 0.02 | 0.01 | 0.01 | 0.01 |
| Deltaproteobacteria_bacterium_SMP-3 | 0.02 | 0.00 | 0.00 | 0.00 | 0.00 | 0.02 | 0.00 | 0.00 | 0.01 | 0.00 | 0.07 | 0.05 | 0.00 | 0.00 | 0.00 | 0.00 | 0.00 | 0.02 | 0.00 | 0.00 | 0.00 |
| marine_metagenome | 0.00 | 0.01 | 0.01 | 0.00 | 0.00 | 0.00 | 0.00 | 0.00 | 0.00 | 0.00 | 0.00 | 0.00 | 0.00 | 0.00 | 0.00 | 0.00 | 0.00 | 0.00 | 0.00 | 0.00 | 0.00 |
| unclassified_SAR324_clade[Marine_group_B] | 0.02 | 0.00 | 0.00 | 0.00 | 0.00 | 0.00 | 0.02 | 0.00 | 0.35 | 0.02 | 0.01 | 0.00 | 0.02 | 0.00 | 0.00 | 0.01 | 0.01 | 0.00 | 0.00 | 0.00 | 0.00 |
| unclassified_Sva0485 | 0.00 | 0.00 | 0.00 | 0.00 | 0.00 | 0.00 | 0.00 | 0.00 | 0.00 | 0.00 | 0.00 | 0.00 | 0.00 | 0.00 | 0.00 | 0.00 | 0.00 | 0.00 | 0.00 | 0.00 | 0.00 |
| Desulfobacca | 0.00 | 0.00 | 0.00 | 0.00 | 0.00 | 0.00 | 0.04 | 0.00 | 0.00 | 0.00 | 0.00 | 0.00 | 0.00 | 0.00 | 0.00 | 0.00 | 0.00 | 0.00 | 0.00 | 0.00 | 0.00 |
| Smithella | 0.01 | 0.01 | 0.01 | 0.01 | 0.00 | 0.00 | 0.00 | 0.00 | 0.00 | 0.00 | 0.01 | 0.00 | 0.00 | 0.00 | 0.00 | 0.00 | 0.00 | 0.00 | 0.00 | 0.00 | 0.00 |
| Syntrophus | 0.00 | 0.00 | 0.00 | 0.00 | 0.00 | 0.00 | 0.02 | 0.00 | 0.00 | 0.00 | 0.00 | 0.00 | 0.00 | 0.00 | 0.00 | 0.00 | 0.00 | 0.00 | 0.00 | 0.00 | 0.00 |
| unclassified_Syntrophobacteraceae | 0.00 | 0.00 | 0.00 | 0.00 | 0.00 | 0.00 | 0.06 | 0.00 | 0.00 | 0.00 | 0.00 | 0.00 | 0.00 | 0.00 | 0.00 | 0.00 | 0.00 | 0.00 | 0.00 | 0.00 | 0.00 |
| Syntrophobacter | 0.00 | 0.00 | 0.00 | 0.00 | 0.00 | 0.00 | 0.00 | 0.00 | 0.00 | 0.00 | 0.00 | 0.00 | 0.00 | 0.00 | 0.00 | 0.00 | 0.00 | 0.00 | 0.00 | 0.00 | 0.00 |
| unclassified_Gammaproteobacteria | 0.36 | 0.09 | 0.09 | 0.18 | 0.16 | 0.48 | 0.03 | 0.02 | 0.01 | 0.02 | 0.05 | 0.01 | 0.04 | 0.04 | 0.09 | 0.00 | 0.01 | 0.01 | 0.01 | 0.00 | 0.01 |
| unclassified_211ds20 | 0.00 | 0.00 | 0.00 | 0.00 | 0.00 | 0.00 | 0.01 | 0.00 | 0.00 | 0.00 | 0.00 | 0.00 | 0.00 | 0.00 | 0.00 | 0.00 | 0.00 | 0.00 | 0.00 | 0.00 | 0.00 |
| Aeromonas | 0.02 | 0.04 | 0.00 | 0.00 | 0.13 | 0.03 | 0.03 | 0.00 | 0.00 | 0.00 | 0.00 | 0.05 | 0.02 | 0.04 | 0.00 | 0.00 | 0.00 | 0.00 | 0.00 | 0.00 | 0.00 |
| Tolumonas | 0.00 | 0.00 | 0.00 | 0.00 | 0.00 | 0.00 | 0.03 | 0.00 | 0.00 | 0.00 | 0.00 | 0.00 | 0.00 | 0.00 | 0.00 | 0.00 | 0.00 | 0.00 | 0.00 | 0.00 | 0.00 |
| unclassified_Alteromonadaceae | 0.00 | 0.00 | 0.01 | 0.00 | 0.00 | 0.01 | 0.00 | 0.00 | 0.00 | 0.04 | 0.10 | 0.06 | 0.05 | 0.07 | 0.12 | 0.01 | 0.05 | 0.04 | 0.00 | 0.01 | 0.01 |
| Alishewanella | 0.00 | 0.00 | 0.00 | 0.00 | 0.00 | 0.00 | 0.00 | 0.00 | 0.00 | 0.00 | 0.00 | 0.00 | 0.05 | 0.08 | 0.01 | 0.00 | 0.00 | 0.00 | 0.00 | 0.00 | 0.00 |
| Alteromonas | 0.00 | 0.00 | 0.00 | 0.00 | 0.01 | 0.00 | 0.00 | 0.00 | 0.00 | 0.01 | 0.00 | 0.00 | 0.01 | 0.00 | 0.00 | 0.02 | 0.01 | 0.01 | 0.01 | 0.03 | 0.01 |
| Rheinheimera | 0.11 | 0.25 | 0.30 | 0.35 | 0.27 | 0.42 | 0.04 | 0.01 | 0.00 | 0.04 | 0.00 | 0.00 | 0.09 | 0.30 | 0.06 | 0.01 | 0.00 | 0.01 | 0.00 | 0.00 | 0.00 |
| Colwellia | 0.24 | 0.04 | 0.31 | 0.02 | 0.01 | 0.01 | 0.01 | 0.07 | 0.04 | 0.01 | 0.01 | 0.00 | 0.02 | 0.01 | 0.03 | 0.01 | 0.02 | 0.01 | 0.01 | 0.01 | 0.02 |
| Litorilituus | 0.01 | 0.09 | 0.01 | 0.00 | 0.01 | 0.00 | 0.00 | 0.01 | 0.00 | 0.00 | 0.01 | 0.02 | 0.00 | 0.01 | 0.01 | 0.00 | 0.03 | 0.01 | 0.01 | 0.02 | 0.02 |
| Thalassotalea | 0.01 | 0.00 | 0.05 | 0.00 | 0.00 | 0.00 | 0.00 | 0.00 | 0.01 | 0.00 | 0.04 | 0.04 | 0.00 | 0.00 | 0.00 | 0.00 | 0.06 | 0.04 | 0.01 | 0.00 | 0.02 |
| Aliidiomarina | 0.00 | 0.00 | 0.00 | 0.03 | 0.00 | 0.00 | 0.00 | 0.00 | 0.00 | 0.00 | 0.00 | 0.00 | 0.00 | 0.00 | 0.00 | 0.00 | 0.00 | 0.00 | 0.00 | 0.00 | 0.00 |
| Idiomarina | 0.01 | 0.00 | 0.00 | 0.00 | 0.00 | 0.03 | 0.00 | 0.01 | 0.01 | 0.03 | 0.00 | 0.01 | 0.06 | 0.12 | 0.10 | 0.00 | 0.00 | 0.01 | 0.00 | 0.00 | 0.00 |
| Marinobacter | 0.03 | 0.00 | 0.00 | 0.00 | 0.00 | 0.00 | 0.03 | 0.01 | 0.00 | 0.00 | 0.01 | 0.00 | 0.03 | 0.00 | 0.00 | 0.00 | 0.00 | 0.00 | 0.00 | 0.01 | 0.00 |
| Moritella | 0.00 | 0.00 | 0.00 | 0.00 | 0.00 | 0.00 | 0.00 | 0.00 | 0.00 | 0.00 | 0.00 | 0.00 | 0.00 | 0.00 | 0.00 | 0.00 | 0.00 | 0.00 | 0.04 | 0.00 | 0.00 |
| unclassified_Pseudoalteromonadaceae | 0.00 | 0.00 | 0.00 | 0.00 | 0.00 | 0.00 | 0.00 | 0.00 | 0.00 | 0.00 | 0.00 | 0.00 | 0.00 | 0.00 | 0.00 | 0.00 | 0.05 | 0.08 | 0.03 | 0.02 | 0.04 |
| Algicola | 0.01 | 0.01 | 0.00 | 0.01 | 0.00 | 0.00 | 0.02 | 0.01 | 0.01 | 0.81 | 4.01 | 3.77 | 0.02 | 0.01 | 0.01 | 0.02 | 0.01 | 0.02 | 0.01 | 0.02 | 0.01 |
| Pseudoalteromonas | 0.27 | 0.20 | 0.30 | 0.10 | 0.10 | 0.14 | 11.18 | 4.56 | 1.27 | 0.19 | 0.19 | 0.26 | 18.72 | 14.52 | 8.25 | 0.16 | 0.37 | 0.30 | 0.27 | 0.22 | 0.10 |
| Psychromonas | 0.02 | 0.03 | 0.04 | 0.00 | 0.00 | 0.00 | 0.00 | 0.00 | 0.02 | 0.00 | 0.00 | 0.00 | 0.00 | 0.00 | 0.00 | 0.00 | 0.00 | 0.00 | 0.00 | 0.00 | 0.00 |
| Psychrobium | 0.00 | 0.00 | 0.00 | 0.00 | 0.00 | 0.00 | 0.00 | 0.00 | 0.00 | 0.00 | 0.00 | 0.00 | 0.00 | 0.00 | 0.00 | 0.00 | 0.13 | 0.05 | 0.00 | 0.00 | 0.00 |
| Shewanella | 1.47 | 0.53 | 1.57 | 0.02 | 0.11 | 0.05 | 0.15 | 0.03 | 0.05 | 0.02 | 0.11 | 0.19 | 0.12 | 0.13 | 0.08 | 0.08 | 0.09 | 0.06 | 0.06 | 0.06 | 0.03 |
| unclassified_Arenicellaceae | 0.00 | 0.04 | 0.04 | 0.00 | 0.00 | 0.00 | 0.01 | 0.01 | 0.00 | 0.00 | 0.00 | 0.01 | 0.00 | 0.00 | 0.00 | 0.00 | 0.00 | 0.00 | 0.00 | 0.00 | 0.00 |
| Arenicella | 0.00 | 0.01 | 0.01 | 0.01 | 0.00 | 0.01 | 0.00 | 0.01 | 0.00 | 0.00 | 0.01 | 0.00 | 0.01 | 0.00 | 0.00 | 0.01 | 0.00 | 0.00 | 0.00 | 0.00 | 0.00 |
| HTCC5015 | 0.07 | 0.10 | 0.05 | 0.01 | 0.00 | 0.00 | 0.05 | 0.12 | 0.19 | 0.00 | 0.04 | 0.05 | 0.04 | 0.08 | 0.04 | 0.00 | 0.06 | 0.04 | 0.03 | 0.00 | 0.02 |
| Perspicuibacter | 0.00 | 0.04 | 0.00 | 0.00 | 0.00 | 0.00 | 0.00 | 0.00 | 0.00 | 0.00 | 0.00 | 0.00 | 0.00 | 0.00 | 0.00 | 0.00 | 0.00 | 0.00 | 0.00 | 0.00 | 0.00 |
| unclassified_B2M28 | 0.00 | 0.00 | 0.00 | 0.00 | 0.00 | 0.00 | 0.05 | 0.00 | 0.00 | 0.00 | 0.00 | 0.00 | 0.00 | 0.00 | 0.00 | 0.00 | 0.00 | 0.00 | 0.00 | 0.00 | 0.00 |
| Laribacter | 0.00 | 0.09 | 0.00 | 0.00 | 0.00 | 0.00 | 0.00 | 0.00 | 0.00 | 0.00 | 0.00 | 0.00 | 0.00 | 0.00 | 0.00 | 0.00 | 0.00 | 0.00 | 0.00 | 0.00 | 0.00 |
| Achromobacter | 0.00 | 0.00 | 0.00 | 0.05 | 0.00 | 0.00 | 0.01 | 0.00 | 0.00 | 0.00 | 0.00 | 0.00 | 0.00 | 0.00 | 0.00 | 0.00 | 0.00 | 0.00 | 0.00 | 0.00 | 0.00 |
| Aquabacterium | 0.03 | 0.07 | 0.07 | 0.00 | 0.08 | 0.14 | 0.01 | 0.00 | 0.11 | 0.00 | 0.00 | 0.01 | 0.02 | 0.01 | 0.01 | 0.00 | 0.00 | 0.00 | 0.00 | 0.00 | 0.00 |
| Burkholderia-Caballeronia-Paraburkholderia | 0.00 | 0.00 | 0.00 | 0.01 | 0.00 | 0.00 | 0.00 | 0.00 | 0.00 | 0.00 | 0.00 | 0.00 | 0.00 | 0.00 | 0.00 | 0.00 | 0.00 | 0.00 | 0.00 | 0.00 | 0.00 |
| Castellaniella | 0.00 | 0.00 | 0.00 | 0.00 | 0.00 | 0.00 | 0.00 | 0.00 | 0.00 | 0.02 | 0.00 | 0.01 | 0.00 | 0.00 | 0.00 | 0.00 | 0.00 | 0.00 | 0.00 | 0.00 | 0.00 |
| Comamonas | 0.00 | 0.03 | 0.00 | 0.00 | 0.14 | 0.00 | 0.03 | 0.00 | 0.00 | 0.00 | 0.00 | 0.00 | 0.01 | 0.02 | 0.01 | 0.00 | 0.00 | 0.00 | 0.00 | 0.00 | 0.00 |
| Cupriavidus | 0.00 | 0.00 | 0.00 | 0.07 | 0.00 | 0.00 | 0.00 | 0.00 | 0.00 | 0.00 | 0.00 | 0.00 | 0.00 | 0.00 | 0.00 | 0.00 | 0.00 | 0.00 | 0.01 | 0.00 | 0.00 |
| Curvibacter | 0.00 | 0.00 | 0.00 | 0.00 | 0.00 | 0.00 | 0.01 | 0.00 | 0.00 | 0.00 | 0.00 | 0.00 | 0.00 | 0.02 | 0.00 | 0.00 | 0.00 | 0.00 | 0.00 | 0.00 | 0.00 |
| Delftia | 0.23 | 0.24 | 0.36 | 0.39 | 0.36 | 0.28 | 0.28 | 0.21 | 0.16 | 0.28 | 0.21 | 0.28 | 0.25 | 0.32 | 0.21 | 0.36 | 0.22 | 0.26 | 0.24 | 0.39 | 0.17 |
| GKS98_freshwater_group | 0.01 | 0.00 | 0.00 | 0.00 | 0.00 | 0.00 | 0.00 | 0.00 | 0.00 | 0.00 | 0.00 | 0.00 | 0.00 | 0.00 | 0.00 | 0.00 | 0.00 | 0.00 | 0.00 | 0.00 | 0.00 |
| Hydrogenophaga | 0.01 | 0.00 | 0.00 | 0.02 | 0.10 | 0.00 | 0.01 | 0.00 | 0.00 | 0.02 | 0.00 | 0.00 | 0.00 | 0.00 | 0.00 | 0.00 | 0.00 | 0.00 | 0.00 | 0.00 | 0.00 |
| Limnobacter | 0.06 | 0.01 | 0.00 | 0.01 | 0.00 | 0.00 | 0.01 | 0.00 | 0.00 | 0.00 | 0.00 | 0.01 | 0.00 | 0.00 | 0.01 | 0.00 | 0.00 | 0.00 | 0.00 | 0.00 | 0.00 |
| Massilia | 0.03 | 0.04 | 0.16 | 0.56 | 0.09 | 0.00 | 0.01 | 0.00 | 0.00 | 0.00 | 0.01 | 0.01 | 0.00 | 0.09 | 0.01 | 0.00 | 0.00 | 0.00 | 0.01 | 0.01 | 0.00 |
| Oxalobacter | 0.00 | 0.00 | 0.00 | 0.00 | 0.00 | 0.00 | 0.01 | 0.00 | 0.00 | 0.00 | 0.00 | 0.00 | 0.00 | 0.00 | 0.00 | 0.00 | 0.00 | 0.00 | 0.00 | 0.00 | 0.00 |
| Paenalcaligenes | 0.00 | 0.00 | 0.00 | 0.00 | 0.00 | 0.00 | 0.01 | 0.00 | 0.00 | 0.00 | 0.00 | 0.00 | 0.00 | 0.01 | 0.00 | 0.00 | 0.00 | 0.00 | 0.00 | 0.00 | 0.00 |
| Parasutterella | 0.00 | 0.00 | 0.00 | 0.00 | 0.00 | 0.00 | 0.01 | 0.00 | 0.00 | 0.00 | 0.00 | 0.00 | 0.00 | 0.00 | 0.00 | 0.00 | 0.00 | 0.00 | 0.00 | 0.00 | 0.00 |
| Paucibacter | 0.01 | 0.05 | 0.03 | 0.05 | 0.00 | 0.02 | 0.03 | 0.00 | 0.01 | 0.06 | 0.00 | 0.01 | 0.02 | 0.05 | 0.00 | 0.00 | 0.00 | 0.00 | 0.00 | 0.00 | 0.00 |
| Polaromonas | 0.00 | 0.00 | 0.00 | 0.04 | 0.00 | 0.00 | 0.00 | 0.00 | 0.00 | 0.00 | 0.00 | 0.00 | 0.00 | 0.00 | 0.00 | 0.00 | 0.00 | 0.00 | 0.00 | 0.00 | 0.00 |
| Pusillimonas | 0.00 | 0.00 | 0.00 | 0.00 | 0.00 | 0.00 | 0.00 | 0.00 | 0.00 | 0.00 | 0.00 | 0.00 | 0.00 | 0.00 | 0.00 | 0.00 | 0.00 | 0.00 | 0.00 | 0.00 | 0.00 |
| Ralstonia | 0.66 | 2.12 | 1.25 | 1.75 | 1.04 | 0.78 | 1.05 | 0.52 | 0.08 | 1.14 | 0.11 | 0.09 | 1.32 | 0.77 | 0.11 | 0.03 | 0.01 | 0.02 | 0.02 | 0.03 | 0.01 |
| Rhodoferax | 0.00 | 0.00 | 0.00 | 0.00 | 0.00 | 0.00 | 0.00 | 0.00 | 0.00 | 0.00 | 0.00 | 0.00 | 0.03 | 0.00 | 0.00 | 0.00 | 0.00 | 0.00 | 0.00 | 0.00 | 0.00 |
| Schlegelella | 0.00 | 0.00 | 0.00 | 0.00 | 0.00 | 0.00 | 0.01 | 0.00 | 0.00 | 0.00 | 0.00 | 0.00 | 0.00 | 0.00 | 0.00 | 0.00 | 0.00 | 0.00 | 0.00 | 0.00 | 0.00 |
| Tepidimonas | 0.00 | 0.00 | 0.00 | 0.00 | 0.00 | 0.00 | 0.00 | 0.00 | 0.00 | 0.00 | 0.00 | 0.00 | 0.00 | 0.02 | 0.00 | 0.00 | 0.00 | 0.00 | 0.00 | 0.00 | 0.00 |
| Variovorax | 0.00 | 0.00 | 0.00 | 0.07 | 0.06 | 0.00 | 0.01 | 0.00 | 0.00 | 0.03 | 0.01 | 0.00 | 0.00 | 0.03 | 0.00 | 0.00 | 0.00 | 0.00 | 0.00 | 0.00 | 0.00 |
| Vogesella | 0.00 | 0.00 | 0.00 | 0.00 | 0.00 | 0.00 | 0.01 | 0.00 | 0.00 | 0.00 | 0.00 | 0.00 | 0.00 | 0.00 | 0.00 | 0.00 | 0.00 | 0.00 | 0.00 | 0.00 | 0.00 |
| Thiobacillus | 0.01 | 0.00 | 0.00 | 0.18 | 0.00 | 0.00 | 0.00 | 0.00 | 0.00 | 0.00 | 0.00 | 0.00 | 0.00 | 0.01 | 0.00 | 0.00 | 0.00 | 0.00 | 0.00 | 0.00 | 0.01 |
| unclassified_Methylophilaceae | 0.00 | 0.00 | 0.00 | 0.00 | 0.00 | 0.00 | 0.11 | 0.00 | 0.00 | 0.00 | 0.00 | 0.00 | 0.00 | 0.00 | 0.00 | 0.00 | 0.00 | 0.00 | 0.00 | 0.00 | 0.00 |
| Methylophilus | 0.01 | 0.06 | 0.02 | 0.04 | 0.06 | 0.01 | 0.00 | 0.00 | 0.00 | 0.07 | 0.00 | 0.01 | 0.00 | 0.00 | 0.00 | 0.00 | 0.00 | 0.00 | 0.00 | 0.00 | 0.00 |
| Methylotenera | 1.04 | 0.41 | 0.51 | 0.31 | 0.18 | 1.73 | 0.13 | 1.02 | 0.35 | 0.25 | 0.47 | 0.35 | 0.53 | 0.14 | 0.16 | 0.05 | 0.07 | 0.19 | 0.18 | 0.05 | 0.03 |
| unclassified_Neisseriaceae | 0.00 | 0.00 | 0.00 | 0.00 | 0.00 | 0.00 | 0.00 | 0.00 | 0.00 | 0.00 | 0.00 | 0.00 | 0.00 | 0.00 | 0.00 | 0.00 | 0.00 | 0.00 | 0.00 | 0.00 | 0.00 |
| Neisseria | 0.00 | 0.09 | 0.00 | 0.08 | 0.10 | 0.00 | 0.04 | 0.01 | 0.00 | 0.09 | 0.02 | 0.01 | 0.00 | 0.00 | 0.03 | 0.00 | 0.00 | 0.00 | 0.00 | 0.00 | 0.00 |
| Snodgrassella | 0.00 | 0.01 | 1.04 | 0.71 | 0.16 | 0.30 | 0.01 | 0.01 | 0.00 | 0.10 | 0.00 | 0.02 | 0.03 | 0.21 | 0.03 | 0.00 | 0.01 | 0.01 | 0.00 | 0.00 | 0.00 |
| MND1 | 0.00 | 0.00 | 0.00 | 0.07 | 0.00 | 0.00 | 0.00 | 0.00 | 0.00 | 0.00 | 0.00 | 0.00 | 0.02 | 0.00 | 0.00 | 0.00 | 0.00 | 0.00 | 0.00 | 0.00 | 0.00 |
| Nitrosomonas | 0.00 | 0.00 | 0.00 | 0.00 | 0.00 | 0.00 | 0.00 | 0.00 | 0.00 | 0.00 | 0.00 | 0.01 | 0.00 | 0.00 | 0.00 | 0.00 | 0.00 | 0.00 | 0.00 | 0.00 | 0.00 |
| Azoarcus | 0.00 | 0.00 | 0.00 | 0.00 | 0.00 | 0.00 | 0.00 | 0.00 | 0.00 | 0.00 | 0.00 | 0.00 | 0.00 | 0.00 | 0.00 | 0.00 | 0.00 | 0.00 | 0.00 | 0.00 | 0.00 |
| C39 | 0.00 | 0.00 | 0.00 | 0.00 | 0.00 | 0.00 | 0.00 | 0.00 | 0.00 | 0.00 | 0.00 | 0.00 | 0.00 | 0.03 | 0.00 | 0.00 | 0.00 | 0.00 | 0.00 | 0.00 | 0.00 |
| Dechloromonas | 0.00 | 0.02 | 0.02 | 0.00 | 0.05 | 0.00 | 0.01 | 0.00 | 0.00 | 0.01 | 0.00 | 0.00 | 0.01 | 0.03 | 0.00 | 0.00 | 0.00 | 0.00 | 0.00 | 0.00 | 0.00 |
| Denitromonas | 0.00 | 0.03 | 0.00 | 0.00 | 0.00 | 0.00 | 0.00 | 0.00 | 0.00 | 0.00 | 0.00 | 0.00 | 0.00 | 0.00 | 0.00 | 0.00 | 0.00 | 0.00 | 0.00 | 0.00 | 0.00 |
| Sulfuritalea | 0.00 | 0.00 | 0.00 | 0.00 | 0.00 | 0.00 | 0.00 | 0.00 | 0.00 | 0.00 | 0.00 | 0.00 | 0.00 | 0.03 | 0.00 | 0.00 | 0.00 | 0.01 | 0.00 | 0.00 | 0.00 |
| Zoogloea | 0.01 | 0.00 | 0.00 | 0.00 | 0.00 | 0.00 | 0.02 | 0.00 | 0.00 | 0.00 | 0.00 | 0.00 | 0.01 | 0.00 | 0.00 | 0.00 | 0.00 | 0.00 | 0.00 | 0.00 | 0.00 |
| unclassified_TRA3-20 | 0.00 | 0.00 | 0.00 | 0.00 | 0.00 | 0.00 | 0.01 | 0.00 | 0.00 | 0.00 | 0.00 | 0.00 | 0.00 | 0.00 | 0.00 | 0.00 | 0.00 | 0.00 | 0.00 | 0.00 | 0.00 |
| unclassified_Cellvibrionaceae | 0.13 | 0.01 | 0.53 | 0.00 | 0.01 | 0.01 | 0.00 | 0.00 | 0.00 | 0.00 | 0.01 | 0.00 | 0.01 | 0.00 | 0.04 | 0.00 | 0.00 | 0.00 | 0.01 | 0.00 | 0.00 |
| Aestuariicella | 0.01 | 0.01 | 0.01 | 0.00 | 0.01 | 0.02 | 0.01 | 0.01 | 0.00 | 0.01 | 0.01 | 0.00 | 0.00 | 0.00 | 0.01 | 0.01 | 0.01 | 0.00 | 0.01 | 0.00 | 0.01 |
| Cellvibrio | 0.00 | 0.00 | 0.00 | 0.07 | 0.00 | 0.00 | 0.00 | 0.00 | 0.00 | 0.00 | 0.00 | 0.00 | 0.00 | 0.00 | 0.00 | 0.00 | 0.00 | 0.00 | 0.00 | 0.00 | 0.00 |
| Marinagarivorans | 0.00 | 0.00 | 0.00 | 0.00 | 0.00 | 0.00 | 0.00 | 0.00 | 0.00 | 0.00 | 0.00 | 0.00 | 0.00 | 0.00 | 0.01 | 0.00 | 0.00 | 0.00 | 0.00 | 0.00 | 0.00 |
| Pseudoteredinibacter | 0.02 | 0.00 | 0.00 | 0.00 | 0.00 | 0.00 | 0.00 | 0.05 | 0.01 | 0.00 | 0.03 | 0.01 | 0.00 | 0.01 | 0.02 | 0.00 | 0.01 | 0.01 | 0.00 | 0.00 | 0.00 |
| unclassified_Halieaceae | 0.00 | 0.00 | 0.01 | 0.00 | 0.01 | 0.00 | 0.00 | 0.00 | 0.01 | 0.00 | 0.00 | 0.00 | 0.00 | 0.00 | 0.00 | 0.00 | 0.00 | 0.01 | 0.00 | 0.00 | 0.00 |
| Haliea | 0.01 | 0.00 | 0.00 | 0.00 | 0.00 | 0.03 | 0.00 | 0.00 | 0.00 | 0.00 | 0.00 | 0.00 | 0.00 | 0.00 | 0.00 | 0.00 | 0.00 | 0.00 | 0.00 | 0.00 | 0.00 |
| Halioglobus | 0.04 | 0.03 | 0.01 | 0.03 | 0.10 | 0.28 | 0.02 | 0.07 | 0.05 | 0.02 | 0.02 | 0.02 | 0.05 | 0.01 | 0.02 | 0.03 | 0.01 | 0.02 | 0.02 | 0.03 | 0.01 |
| Marimicrobium | 0.00 | 0.00 | 0.00 | 0.00 | 0.00 | 0.00 | 0.00 | 0.00 | 0.00 | 0.00 | 0.01 | 0.00 | 0.00 | 0.00 | 0.00 | 0.00 | 0.00 | 0.00 | 0.00 | 0.00 | 0.00 |
| Microbulbifer | 0.00 | 0.00 | 0.00 | 0.00 | 0.00 | 0.00 | 0.01 | 0.00 | 0.00 | 0.00 | 0.00 | 0.00 | 0.00 | 0.00 | 0.00 | 0.00 | 0.00 | 0.00 | 0.00 | 0.00 | 0.00 |
| C1-B045 | 0.00 | 0.00 | 0.05 | 0.00 | 0.00 | 0.00 | 0.00 | 0.00 | 0.00 | 0.00 | 0.00 | 0.00 | 0.00 | 0.00 | 0.00 | 0.00 | 0.00 | 0.00 | 0.00 | 0.00 | 0.00 |
| Porticoccus | 0.01 | 0.00 | 0.01 | 0.07 | 0.04 | 0.05 | 0.00 | 0.01 | 0.00 | 0.00 | 0.01 | 0.01 | 0.01 | 0.00 | 0.00 | 0.00 | 0.00 | 0.01 | 0.01 | 0.01 | 0.00 |
| SAR92_clade | 0.00 | 0.00 | 0.00 | 0.00 | 0.06 | 0.00 | 0.00 | 0.00 | 0.00 | 0.00 | 0.00 | 0.00 | 0.00 | 0.00 | 0.00 | 0.00 | 0.00 | 0.00 | 0.00 | 0.00 | 0.00 |
| unclassified_Spongiibacteraceae | 0.00 | 0.00 | 0.00 | 0.00 | 0.00 | 0.00 | 0.00 | 0.00 | 0.00 | 0.00 | 0.00 | 0.00 | 0.01 | 0.00 | 0.00 | 0.00 | 0.00 | 0.00 | 0.00 | 0.00 | 0.00 |
| BD1-7_clade | 0.02 | 0.00 | 0.01 | 0.00 | 0.00 | 0.00 | 0.00 | 0.01 | 0.02 | 0.00 | 0.01 | 0.01 | 0.01 | 0.01 | 0.01 | 0.00 | 0.01 | 0.01 | 0.00 | 0.01 | 0.01 |
| Spongiibacter | 0.00 | 0.00 | 0.00 | 0.00 | 0.00 | 0.00 | 0.00 | 0.00 | 0.00 | 0.00 | 0.00 | 0.02 | 0.00 | 0.00 | 0.01 | 0.00 | 0.00 | 0.00 | 0.00 | 0.00 | 0.00 |
| Coxiella | 0.29 | 0.16 | 0.14 | 0.11 | 0.06 | 0.03 | 0.01 | 0.02 | 0.01 | 0.01 | 0.00 | 0.00 | 0.04 | 0.01 | 0.04 | 0.00 | 0.00 | 0.00 | 0.00 | 0.00 | 0.00 |
| unclassified_Diplorickettsiaceae | 0.00 | 0.00 | 0.02 | 0.00 | 0.00 | 0.00 | 0.00 | 0.00 | 0.00 | 0.00 | 0.00 | 0.00 | 0.00 | 0.00 | 0.00 | 0.00 | 0.00 | 0.00 | 0.00 | 0.00 | 0.00 |
| Aquicella | 0.13 | 0.26 | 0.15 | 0.06 | 0.02 | 0.17 | 0.08 | 0.03 | 0.02 | 0.01 | 0.02 | 0.04 | 0.03 | 0.01 | 0.06 | 0.01 | 0.02 | 0.01 | 0.01 | 0.02 | 0.01 |
| Diplorickettsia | 0.00 | 0.00 | 0.00 | 0.00 | 0.00 | 0.00 | 0.00 | 0.00 | 0.00 | 0.00 | 0.00 | 0.00 | 0.00 | 0.00 | 0.03 | 0.00 | 0.00 | 0.00 | 0.00 | 0.00 | 0.00 |
| unclassified_EC3 | 0.00 | 0.00 | 0.00 | 0.00 | 0.00 | 0.00 | 0.00 | 0.01 | 0.03 | 0.00 | 0.00 | 0.01 | 0.02 | 0.00 | 0.02 | 0.00 | 0.00 | 0.00 | 0.00 | 0.00 | 0.00 |
| unclassified_EPR3968-O8a-Bc78 | 0.00 | 0.00 | 0.00 | 0.00 | 0.00 | 0.00 | 0.00 | 0.00 | 0.00 | 0.00 | 0.00 | 0.00 | 0.00 | 0.01 | 0.00 | 0.00 | 0.00 | 0.00 | 0.00 | 0.00 | 0.00 |
| unclassified_Ectothiorhodospiraceae | 0.01 | 0.04 | 0.00 | 0.00 | 0.00 | 0.04 | 0.00 | 0.00 | 0.00 | 0.00 | 0.00 | 0.00 | 0.00 | 0.00 | 0.00 | 0.00 | 0.00 | 0.00 | 0.00 | 0.00 | 0.00 |
| Thiogranum | 0.00 | 0.00 | 0.00 | 0.00 | 0.00 | 0.00 | 0.01 | 0.00 | 0.00 | 0.00 | 0.00 | 0.00 | 0.00 | 0.00 | 0.00 | 0.00 | 0.00 | 0.00 | 0.00 | 0.00 | 0.00 |
| Thioalkalispira | 0.00 | 0.00 | 0.00 | 0.00 | 0.00 | 0.00 | 0.03 | 0.00 | 0.00 | 0.00 | 0.00 | 0.00 | 0.00 | 0.00 | 0.00 | 0.00 | 0.00 | 0.00 | 0.00 | 0.00 | 0.00 |
| Citrobacter | 0.05 | 0.15 | 0.11 | 0.14 | 0.22 | 0.11 | 0.10 | 0.02 | 0.02 | 0.16 | 0.02 | 0.01 | 0.07 | 0.32 | 0.04 | 0.00 | 0.00 | 0.00 | 0.01 | 0.00 | 0.01 |
| Escherichia-Shigella | 0.00 | 0.00 | 0.02 | 0.00 | 0.00 | 0.00 | 0.03 | 0.00 | 0.00 | 0.00 | 0.00 | 0.00 | 0.00 | 0.00 | 0.00 | 0.00 | 0.00 | 0.00 | 0.00 | 0.00 | 0.00 |
| Plesiomonas | 0.00 | 0.00 | 0.00 | 0.00 | 0.00 | 0.12 | 0.03 | 0.00 | 0.00 | 0.00 | 0.00 | 0.00 | 0.00 | 0.00 | 0.00 | 0.02 | 0.00 | 0.00 | 0.00 | 0.00 | 0.00 |
| Shimwellia | 0.00 | 0.00 | 0.00 | 0.00 | 0.01 | 0.00 | 0.00 | 0.00 | 0.00 | 0.00 | 0.00 | 0.00 | 0.00 | 0.00 | 0.00 | 0.00 | 0.00 | 0.00 | 0.00 | 0.00 | 0.00 |
| unclassified_F9P41300-M23 | 0.01 | 0.00 | 0.00 | 0.00 | 0.00 | 0.00 | 0.01 | 0.00 | 0.00 | 0.00 | 0.00 | 0.01 | 0.00 | 0.00 | 0.00 | 0.00 | 0.00 | 0.00 | 0.00 | 0.00 | 0.00 |
| unclassified_Francisellaceae | 0.00 | 0.00 | 0.00 | 0.00 | 0.00 | 0.00 | 0.00 | 0.00 | 0.00 | 0.00 | 0.00 | 0.00 | 0.00 | 0.02 | 0.01 | 0.00 | 0.00 | 0.00 | 0.00 | 0.00 | 0.00 |
| Francisella | 0.10 | 0.10 | 0.06 | 0.03 | 0.09 | 0.10 | 3.36 | 0.85 | 0.67 | 1.00 | 1.39 | 1.46 | 5.91 | 3.36 | 7.50 | 0.05 | 0.01 | 0.03 | 0.04 | 0.05 | 0.02 |
| unclassified_Gammaproteobacteria_Incertae_Sedis | 0.01 | 0.00 | 0.00 | 0.00 | 0.00 | 0.00 | 0.01 | 0.00 | 0.00 | 0.00 | 0.00 | 0.00 | 0.00 | 0.00 | 0.00 | 0.00 | 0.00 | 0.00 | 0.00 | 0.00 | 0.00 |
| Alkalimarinus | 0.00 | 0.00 | 0.00 | 0.00 | 0.00 | 0.00 | 0.00 | 0.00 | 0.02 | 0.00 | 0.01 | 0.03 | 0.02 | 0.00 | 0.00 | 0.00 | 0.00 | 0.00 | 0.00 | 0.00 | 0.00 |
| Candidatus_Berkiella | 0.01 | 0.00 | 0.00 | 0.00 | 0.01 | 0.02 | 0.03 | 0.00 | 0.00 | 0.00 | 0.00 | 0.02 | 0.00 | 0.00 | 0.01 | 0.00 | 0.01 | 0.01 | 0.01 | 0.00 | 0.00 |
| Marinicella | 0.06 | 0.07 | 0.08 | 0.07 | 0.06 | 0.05 | 0.06 | 0.07 | 0.05 | 0.04 | 0.16 | 0.06 | 0.06 | 0.07 | 0.04 | 0.04 | 0.04 | 0.06 | 0.06 | 0.06 | 0.04 |
| unclassified_JTB23 | 0.00 | 0.00 | 0.00 | 0.00 | 0.00 | 0.00 | 0.00 | 0.00 | 0.00 | 0.00 | 0.00 | 0.00 | 0.00 | 0.00 | 0.02 | 0.00 | 0.00 | 0.00 | 0.00 | 0.00 | 0.00 |
| unclassified_KI89A_clade | 0.03 | 0.00 | 0.00 | 0.00 | 0.00 | 0.00 | 0.02 | 0.00 | 0.00 | 0.00 | 0.00 | 0.00 | 0.00 | 0.00 | 0.00 | 0.00 | 0.00 | 0.00 | 0.00 | 0.00 | 0.00 |
| unclassified_Legionellaceae | 0.56 | 0.19 | 0.26 | 0.03 | 0.01 | 0.08 | 0.16 | 0.04 | 0.05 | 0.05 | 0.05 | 0.10 | 0.06 | 0.13 | 0.18 | 0.01 | 0.02 | 0.05 | 0.13 | 0.02 | 0.01 |
| Legionella | 0.01 | 0.00 | 0.04 | 0.02 | 0.01 | 0.00 | 0.09 | 0.04 | 0.01 | 0.29 | 0.20 | 0.35 | 0.31 | 0.16 | 0.90 | 0.01 | 0.01 | 0.01 | 0.01 | 0.01 | 0.00 |
| unclassified_MBAE14 | 0.25 | 0.11 | 0.23 | 0.01 | 0.01 | 0.01 | 0.01 | 0.01 | 0.02 | 0.01 | 0.00 | 0.00 | 0.01 | 0.00 | 0.01 | 0.01 | 0.00 | 0.01 | 0.04 | 0.04 | 0.01 |
| gamma_proteobacterium_enrichment_culture_clone_TT-311 | 0.00 | 0.00 | 0.00 | 0.00 | 0.00 | 0.00 | 0.00 | 0.00 | 0.00 | 0.00 | 0.02 | 0.00 | 0.00 | 0.00 | 0.00 | 0.00 | 0.00 | 0.00 | 0.00 | 0.00 | 0.00 |
| unclassified_Methylomonaceae | 0.00 | 0.00 | 0.00 | 0.00 | 0.00 | 0.00 | 0.00 | 0.00 | 0.00 | 0.00 | 0.00 | 0.00 | 0.00 | 0.00 | 0.00 | 0.00 | 0.00 | 0.00 | 0.00 | 0.00 | 0.00 |
| Crenothrix | 0.00 | 0.00 | 0.00 | 0.07 | 0.00 | 0.00 | 0.00 | 0.00 | 0.00 | 0.00 | 0.00 | 0.00 | 0.00 | 0.00 | 0.00 | 0.00 | 0.00 | 0.00 | 0.00 | 0.00 | 0.00 |
| Methylomonas | 0.00 | 0.00 | 0.00 | 0.00 | 0.00 | 0.00 | 0.02 | 0.00 | 0.00 | 0.00 | 0.00 | 0.00 | 0.00 | 0.00 | 0.00 | 0.00 | 0.00 | 0.00 | 0.00 | 0.00 | 0.00 |
| unclassified_Milano-WF1B-44 | 0.01 | 0.00 | 0.04 | 0.00 | 0.00 | 0.05 | 0.01 | 0.03 | 0.02 | 0.02 | 0.00 | 0.01 | 0.01 | 0.00 | 0.01 | 0.01 | 0.00 | 0.00 | 0.00 | 0.00 | 0.00 |
| unclassified_Methylophagaceae | 0.00 | 0.00 | 0.00 | 0.00 | 0.00 | 0.00 | 0.00 | 0.00 | 0.00 | 0.00 | 0.00 | 0.00 | 0.00 | 0.00 | 0.00 | 0.00 | 0.00 | 0.00 | 0.00 | 0.00 | 0.00 |
| Methylophaga | 0.05 | 0.00 | 0.06 | 0.42 | 0.31 | 0.60 | 0.02 | 0.03 | 0.03 | 0.03 | 0.04 | 0.09 | 0.04 | 0.00 | 0.01 | 0.00 | 0.00 | 0.01 | 0.01 | 0.01 | 0.00 |
| Alcanivorax | 0.00 | 0.00 | 0.00 | 0.00 | 0.00 | 0.05 | 0.00 | 0.00 | 0.00 | 0.00 | 0.00 | 0.01 | 0.00 | 0.00 | 0.00 | 0.00 | 0.00 | 0.00 | 0.00 | 0.00 | 0.00 |
| Endozoicomonas | 0.10 | 0.36 | 0.30 | 0.15 | 0.20 | 0.17 | 0.19 | 0.09 | 0.08 | 0.14 | 0.12 | 0.15 | 0.10 | 0.16 | 0.11 | 0.11 | 0.11 | 0.08 | 0.10 | 0.16 | 0.07 |
| Kistimonas | 0.00 | 0.00 | 0.00 | 0.00 | 0.00 | 0.00 | 0.00 | 0.00 | 0.00 | 0.00 | 0.00 | 0.00 | 0.00 | 0.00 | 0.00 | 0.00 | 0.00 | 0.00 | 0.00 | 0.00 | 0.00 |
| Cobetia | 0.01 | 0.00 | 0.00 | 0.00 | 0.00 | 0.00 | 0.00 | 0.00 | 0.00 | 0.00 | 0.00 | 0.00 | 0.00 | 0.00 | 0.00 | 0.00 | 0.00 | 0.00 | 0.00 | 0.00 | 0.00 |
| Halomonas | 0.01 | 0.01 | 0.00 | 0.00 | 0.00 | 0.03 | 0.06 | 0.00 | 0.00 | 0.03 | 0.01 | 0.00 | 0.01 | 0.06 | 0.01 | 0.00 | 0.00 | 0.00 | 0.00 | 0.00 | 0.00 |
| Oceanospirillum | 0.00 | 0.00 | 0.00 | 0.00 | 0.00 | 0.00 | 0.00 | 0.00 | 0.00 | 0.00 | 0.00 | 0.00 | 0.01 | 0.00 | 0.00 | 0.00 | 0.27 | 0.06 | 0.00 | 0.00 | 0.00 |
| Aliikangiella | 0.00 | 0.00 | 0.03 | 0.01 | 0.00 | 0.08 | 0.00 | 0.00 | 0.00 | 0.00 | 0.00 | 0.00 | 0.00 | 0.00 | 0.00 | 0.00 | 0.00 | 0.00 | 0.00 | 0.00 | 0.00 |
| Kangiella | 0.06 | 0.04 | 0.04 | 0.03 | 0.08 | 0.09 | 0.00 | 0.04 | 0.01 | 0.01 | 0.02 | 0.01 | 0.03 | 0.00 | 0.02 | 0.00 | 0.01 | 0.00 | 0.01 | 0.01 | 0.00 |
| Marinomonas | 0.00 | 0.00 | 0.00 | 0.01 | 0.00 | 0.00 | 0.00 | 0.00 | 0.00 | 0.00 | 0.00 | 0.00 | 0.00 | 0.00 | 0.00 | 0.00 | 0.00 | 0.00 | 0.00 | 0.00 | 0.00 |
| unclassified_Nitrincolaceae | 0.00 | 0.00 | 0.00 | 0.00 | 0.00 | 0.02 | 0.00 | 0.00 | 0.00 | 0.00 | 0.00 | 0.00 | 0.00 | 0.00 | 0.00 | 0.00 | 0.00 | 0.00 | 0.00 | 0.00 | 0.00 |
| Amphritea | 0.19 | 0.16 | 0.31 | 0.18 | 0.21 | 0.17 | 0.17 | 0.11 | 0.09 | 11.42 | 6.22 | 10.71 | 0.43 | 0.37 | 0.20 | 5.44 | 3.97 | 1.83 | 10.42 | 18.15 | 4.35 |
| Marinobacterium | 0.03 | 0.00 | 0.00 | 0.01 | 0.00 | 0.03 | 0.00 | 0.00 | 0.00 | 0.00 | 0.01 | 0.00 | 0.00 | 0.00 | 0.00 | 0.00 | 0.00 | 0.00 | 0.00 | 0.00 | 0.00 |
| Neptuniibacter | 0.03 | 0.02 | 0.04 | 0.04 | 0.03 | 0.11 | 0.08 | 0.20 | 0.10 | 0.59 | 0.63 | 1.02 | 0.16 | 0.17 | 0.18 | 0.05 | 1.65 | 1.51 | 0.61 | 0.57 | 0.52 |
| Neptunomonas | 0.02 | 0.03 | 0.03 | 0.01 | 0.01 | 0.02 | 0.01 | 0.01 | 0.00 | 0.01 | 0.01 | 0.02 | 0.02 | 0.01 | 0.01 | 0.01 | 0.01 | 0.02 | 0.01 | 0.03 | 0.01 |
| Oleiphilus | 0.07 | 0.01 | 0.03 | 0.00 | 0.00 | 0.00 | 0.00 | 0.00 | 0.00 | 0.00 | 0.00 | 0.00 | 0.00 | 0.00 | 0.01 | 0.00 | 0.00 | 0.00 | 0.00 | 0.00 | 0.00 |
| Pseudohongiella | 0.12 | 0.14 | 0.00 | 0.00 | 0.00 | 0.01 | 0.02 | 0.04 | 0.02 | 0.00 | 0.01 | 0.00 | 0.05 | 0.00 | 0.01 | 0.00 | 0.00 | 0.00 | 0.00 | 0.00 | 0.00 |
| unclassified_Saccharospirillaceae | 0.01 | 0.00 | 0.00 | 0.00 | 0.00 | 0.00 | 0.00 | 0.00 | 0.00 | 0.00 | 0.00 | 0.00 | 0.00 | 0.00 | 0.00 | 0.00 | 0.00 | 0.00 | 0.00 | 0.00 | 0.00 |
| Bermanella | 0.01 | 0.00 | 0.00 | 0.00 | 0.00 | 0.00 | 0.00 | 0.00 | 0.00 | 0.00 | 0.00 | 0.00 | 0.00 | 0.00 | 0.00 | 0.00 | 0.00 | 0.00 | 0.00 | 0.00 | 0.00 |
| Oceanobacter | 0.02 | 0.00 | 0.00 | 0.00 | 0.00 | 0.00 | 0.00 | 0.00 | 0.00 | 0.00 | 0.00 | 0.00 | 0.00 | 0.00 | 0.00 | 0.00 | 0.00 | 0.00 | 0.00 | 0.00 | 0.00 |
| Oleibacter | 0.00 | 0.00 | 0.00 | 0.00 | 0.00 | 0.00 | 0.02 | 0.07 | 0.03 | 0.00 | 0.00 | 0.00 | 0.01 | 0.00 | 0.00 | 0.00 | 0.00 | 0.00 | 0.00 | 0.00 | 0.00 |
| Oleispira | 0.01 | 0.01 | 0.03 | 0.00 | 0.00 | 0.00 | 0.00 | 0.02 | 0.00 | 0.00 | 0.00 | 0.00 | 0.04 | 0.00 | 0.03 | 0.00 | 0.00 | 0.00 | 0.00 | 0.00 | 0.00 |
| Saccharospirillum | 0.01 | 0.00 | 0.00 | 0.00 | 0.00 | 0.00 | 0.00 | 0.00 | 0.00 | 0.00 | 0.00 | 0.00 | 0.00 | 0.00 | 0.00 | 0.00 | 0.00 | 0.00 | 0.00 | 0.00 | 0.00 |
| Thalassolituus | 0.00 | 0.00 | 0.00 | 0.00 | 0.00 | 0.02 | 0.00 | 0.00 | 0.00 | 0.05 | 0.10 | 0.13 | 0.00 | 0.00 | 0.00 | 0.00 | 0.01 | 0.00 | 0.00 | 0.00 | 0.00 |
| unclassified_PLTA13 | 0.00 | 0.00 | 0.00 | 0.00 | 0.00 | 0.00 | 0.01 | 0.00 | 0.00 | 0.00 | 0.00 | 0.00 | 0.00 | 0.00 | 0.00 | 0.00 | 0.00 | 0.00 | 0.00 | 0.00 | 0.00 |
| Actinobacillus | 0.01 | 0.01 | 0.00 | 0.07 | 0.04 | 0.06 | 0.02 | 0.01 | 0.00 | 0.01 | 0.00 | 0.02 | 0.02 | 0.04 | 0.01 | 0.00 | 0.00 | 0.00 | 0.00 | 0.00 | 0.00 |
| Aggregatibacter | 0.00 | 0.00 | 0.00 | 0.00 | 0.00 | 0.00 | 0.00 | 0.00 | 0.00 | 0.00 | 0.00 | 0.00 | 0.00 | 0.02 | 0.00 | 0.00 | 0.00 | 0.00 | 0.00 | 0.00 | 0.00 |
| Haemophilus | 0.00 | 0.05 | 0.01 | 0.02 | 0.00 | 0.00 | 0.04 | 0.02 | 0.00 | 0.04 | 0.00 | 0.00 | 0.01 | 0.00 | 0.01 | 0.00 | 0.00 | 0.00 | 0.00 | 0.00 | 0.00 |
| Candidatus_Endoecteinascidia | 0.51 | 0.34 | 0.17 | 0.44 | 0.35 | 0.50 | 0.04 | 0.02 | 0.00 | 0.00 | 0.02 | 0.02 | 0.01 | 0.01 | 0.06 | 0.01 | 0.00 | 0.00 | 0.00 | 0.00 | 0.01 |
| Acinetobacter | 1.24 | 2.78 | 2.80 | 3.98 | 4.98 | 3.23 | 2.53 | 0.50 | 0.40 | 2.94 | 0.64 | 0.85 | 1.93 | 4.14 | 0.95 | 0.10 | 0.08 | 0.09 | 0.17 | 0.08 | 0.05 |
| Alkanindiges | 0.00 | 0.00 | 0.00 | 0.00 | 0.03 | 0.00 | 0.00 | 0.00 | 0.00 | 0.00 | 0.00 | 0.00 | 0.00 | 0.00 | 0.00 | 0.00 | 0.00 | 0.00 | 0.00 | 0.00 | 0.00 |
| Cavicella | 0.00 | 0.00 | 0.00 | 0.00 | 0.02 | 0.00 | 0.00 | 0.00 | 0.00 | 0.00 | 0.00 | 0.00 | 0.00 | 0.00 | 0.00 | 0.00 | 0.00 | 0.00 | 0.00 | 0.00 | 0.00 |
| Enhydrobacter | 0.05 | 0.24 | 0.03 | 0.01 | 0.11 | 0.11 | 0.04 | 0.00 | 0.00 | 0.03 | 0.00 | 0.02 | 0.13 | 0.10 | 0.00 | 0.00 | 0.00 | 0.00 | 0.01 | 0.00 | 0.00 |
| Paraperlucidibaca | 0.00 | 0.00 | 0.03 | 0.00 | 0.00 | 0.00 | 0.00 | 0.00 | 0.00 | 0.00 | 0.00 | 0.00 | 0.00 | 0.00 | 0.00 | 0.00 | 0.00 | 0.00 | 0.00 | 0.00 | 0.00 |
| Perlucidibaca | 0.01 | 0.00 | 0.00 | 0.00 | 0.00 | 0.00 | 0.00 | 0.00 | 0.00 | 0.00 | 0.00 | 0.00 | 0.00 | 0.00 | 0.00 | 0.00 | 0.00 | 0.00 | 0.00 | 0.00 | 0.00 |
| Psychrobacter | 0.01 | 0.03 | 0.00 | 0.01 | 0.01 | 0.04 | 0.08 | 0.00 | 0.00 | 0.01 | 0.00 | 0.00 | 0.02 | 0.00 | 0.04 | 0.00 | 0.01 | 0.01 | 0.01 | 0.01 | 0.00 |
| Pseudomonas | 11.85 | 24.48 | 30.13 | 31.81 | 51.43 | 35.40 | 37.52 | 7.65 | 5.27 | 32.65 | 8.90 | 10.01 | 11.22 | 26.02 | 5.83 | 0.94 | 0.54 | 0.61 | 1.13 | 0.96 | 0.47 |
| Alkanibacter | 0.00 | 0.00 | 0.00 | 0.00 | 0.00 | 0.00 | 0.01 | 0.00 | 0.00 | 0.00 | 0.00 | 0.00 | 0.00 | 0.00 | 0.00 | 0.00 | 0.00 | 0.00 | 0.00 | 0.00 | 0.00 |
| unclassified_Steroidobacteraceae | 0.00 | 0.00 | 0.00 | 0.00 | 0.00 | 0.00 | 0.03 | 0.00 | 0.00 | 0.00 | 0.00 | 0.00 | 0.04 | 0.00 | 0.00 | 0.00 | 0.00 | 0.00 | 0.00 | 0.00 | 0.00 |
| Steroidobacter | 0.00 | 0.00 | 0.00 | 0.03 | 0.00 | 0.00 | 0.00 | 0.00 | 0.00 | 0.00 | 0.00 | 0.00 | 0.00 | 0.00 | 0.00 | 0.00 | 0.00 | 0.00 | 0.00 | 0.00 | 0.00 |
| Woeseia | 0.03 | 0.05 | 0.05 | 0.00 | 0.00 | 0.00 | 0.05 | 0.00 | 0.01 | 0.01 | 0.00 | 0.01 | 0.05 | 0.00 | 0.01 | 0.00 | 0.00 | 0.00 | 0.00 | 0.00 | 0.00 |
| Granulosicoccus | 0.03 | 0.02 | 0.00 | 0.00 | 0.00 | 0.00 | 0.00 | 0.00 | 0.00 | 0.00 | 0.00 | 0.01 | 0.00 | 0.00 | 0.00 | 0.00 | 0.00 | 0.00 | 0.00 | 0.00 | 0.00 |
| endosymbionts | 0.00 | 0.02 | 0.00 | 0.00 | 0.00 | 0.00 | 0.00 | 0.00 | 0.00 | 0.00 | 0.00 | 0.00 | 0.00 | 0.00 | 0.00 | 0.00 | 0.00 | 0.00 | 0.00 | 0.00 | 0.00 |
| Cocleimonas | 0.00 | 0.00 | 0.00 | 0.00 | 0.11 | 0.00 | 0.02 | 0.00 | 0.00 | 0.00 | 0.00 | 0.00 | 0.01 | 0.00 | 0.00 | 0.00 | 0.00 | 0.00 | 0.00 | 0.00 | 0.00 |
| Leucothrix | 0.01 | 0.03 | 0.03 | 0.00 | 0.00 | 0.02 | 0.01 | 0.00 | 0.23 | 0.00 | 0.00 | 0.00 | 0.01 | 0.00 | 0.00 | 0.00 | 0.00 | 0.01 | 0.00 | 0.00 | 0.00 |
| Photobacterium | 0.21 | 0.37 | 0.43 | 0.01 | 0.01 | 0.00 | 0.01 | 0.00 | 0.17 | 0.01 | 0.01 | 0.00 | 0.02 | 0.02 | 0.04 | 0.44 | 0.07 | 0.10 | 0.56 | 0.05 | 0.03 |
| Vibrio | 2.81 | 0.91 | 2.04 | 0.72 | 1.16 | 1.00 | 0.60 | 0.41 | 1.77 | 1.15 | 3.14 | 1.60 | 0.70 | 0.73 | 0.66 | 80.07 | 3.00 | 14.00 | 18.15 | 28.38 | 17.92 |
| Dokdonella | 0.00 | 0.00 | 0.00 | 0.00 | 0.00 | 0.00 | 0.00 | 0.00 | 0.00 | 0.02 | 0.00 | 0.00 | 0.00 | 0.00 | 0.00 | 0.00 | 0.00 | 0.00 | 0.00 | 0.00 | 0.00 |
| Metallibacterium | 0.00 | 0.00 | 0.00 | 0.00 | 0.01 | 0.00 | 0.00 | 0.00 | 0.00 | 0.00 | 0.00 | 0.00 | 0.00 | 0.00 | 0.00 | 0.00 | 0.00 | 0.00 | 0.00 | 0.00 | 0.00 |
| unclassified_Xanthomonadaceae | 0.00 | 0.00 | 0.00 | 0.00 | 0.00 | 0.00 | 0.00 | 0.00 | 0.00 | 0.00 | 0.00 | 0.00 | 0.00 | 0.00 | 0.01 | 0.00 | 0.00 | 0.00 | 0.00 | 0.00 | 0.00 |
| Lysobacter | 0.00 | 0.00 | 0.00 | 0.01 | 0.00 | 0.00 | 0.00 | 0.00 | 0.00 | 0.00 | 0.00 | 0.00 | 0.00 | 0.00 | 0.00 | 0.00 | 0.00 | 0.00 | 0.00 | 0.00 | 0.00 |
| Stenotrophomonas | 0.09 | 0.22 | 0.16 | 0.08 | 0.29 | 0.10 | 0.10 | 0.04 | 0.05 | 0.18 | 0.06 | 0.05 | 0.08 | 0.28 | 0.07 | 0.06 | 0.07 | 0.09 | 0.07 | 0.07 | 0.05 |
| unclassified_pItb-vmat-80 | 0.00 | 0.00 | 0.00 | 0.00 | 0.00 | 0.00 | 0.00 | 0.00 | 0.00 | 0.02 | 0.00 | 0.00 | 0.00 | 0.00 | 0.00 | 0.00 | 0.00 | 0.00 | 0.00 | 0.00 | 0.00 |
| unclassified_Leptospiraceae | 0.00 | 0.00 | 0.00 | 0.00 | 0.00 | 0.00 | 0.00 | 0.00 | 0.00 | 0.00 | 0.00 | 0.00 | 0.00 | 0.00 | 0.00 | 0.00 | 0.00 | 0.00 | 0.00 | 0.00 | 0.00 |
| Brevinema | 0.00 | 0.00 | 0.00 | 0.00 | 0.03 | 0.00 | 0.00 | 0.00 | 0.00 | 0.00 | 0.00 | 0.00 | 0.00 | 0.00 | 0.00 | 0.00 | 0.00 | 0.00 | 0.00 | 0.00 | 0.00 |
| unclassified_Spirochaetaceae | 0.00 | 0.00 | 0.00 | 0.00 | 0.00 | 0.00 | 0.00 | 0.00 | 0.00 | 0.02 | 0.00 | 0.00 | 0.00 | 0.00 | 0.00 | 0.00 | 0.00 | 0.00 | 0.00 | 0.00 | 0.00 |
| GWE2-31-10 | 0.00 | 0.00 | 0.00 | 0.00 | 0.00 | 0.00 | 0.00 | 0.00 | 0.00 | 0.00 | 0.00 | 0.01 | 0.00 | 0.00 | 0.00 | 0.00 | 0.00 | 0.00 | 0.00 | 0.00 | 0.00 |
| Sediminispirochaeta | 0.00 | 0.00 | 0.00 | 0.00 | 0.00 | 0.00 | 0.02 | 0.00 | 0.00 | 0.00 | 0.00 | 0.00 | 0.00 | 0.00 | 0.00 | 0.00 | 0.00 | 0.00 | 0.00 | 0.00 | 0.00 |
| Sphaerochaeta | 0.00 | 0.00 | 0.00 | 0.00 | 0.00 | 0.00 | 0.00 | 0.00 | 0.00 | 0.00 | 0.00 | 0.00 | 0.00 | 0.00 | 0.00 | 0.00 | 0.00 | 0.00 | 0.01 | 0.02 | 0.00 |
| Spirochaeta_2 | 0.00 | 0.00 | 0.00 | 0.00 | 0.00 | 0.00 | 0.02 | 0.00 | 0.00 | 0.00 | 0.00 | 0.00 | 0.00 | 0.00 | 0.00 | 0.06 | 0.00 | 0.01 | 0.62 | 0.18 | 0.02 |
| Treponema_2 | 0.02 | 0.00 | 0.00 | 0.26 | 0.00 | 0.05 | 0.00 | 0.00 | 0.01 | 0.00 | 0.00 | 0.00 | 0.00 | 0.00 | 0.01 | 0.00 | 0.00 | 0.00 | 0.00 | 0.00 | 0.00 |
| Dethiosulfovibrio | 0.00 | 0.00 | 0.08 | 0.00 | 0.00 | 0.00 | 0.00 | 0.00 | 0.00 | 0.00 | 0.00 | 0.00 | 0.00 | 0.00 | 0.00 | 0.00 | 0.00 | 0.00 | 0.00 | 0.00 | 0.00 |
| unclassified_Mollicutes_RF39 | 0.00 | 0.00 | 0.00 | 0.06 | 0.00 | 0.00 | 0.04 | 0.00 | 0.00 | 0.00 | 0.00 | 0.00 | 0.00 | 0.03 | 0.00 | 0.00 | 0.00 | 0.00 | 0.00 | 0.00 | 0.00 |
| unclassified_Mycoplasmataceae | 0.00 | 0.00 | 0.00 | 0.00 | 0.00 | 0.00 | 0.01 | 0.00 | 0.00 | 0.00 | 0.00 | 0.00 | 0.00 | 0.00 | 0.00 | 0.00 | 0.00 | 0.00 | 0.00 | 0.00 | 0.00 |
| Mycoplasma | 0.13 | 0.29 | 0.08 | 0.02 | 0.09 | 0.15 | 0.02 | 0.02 | 0.03 | 0.03 | 0.02 | 0.02 | 0.02 | 0.03 | 0.02 | 0.05 | 0.04 | 0.03 | 0.03 | 0.03 | 0.03 |
| Candidatus_Udaeobacter | 0.00 | 0.00 | 0.00 | 0.00 | 0.01 | 0.00 | 0.01 | 0.00 | 0.00 | 0.00 | 0.00 | 0.00 | 0.00 | 0.00 | 0.00 | 0.00 | 0.01 | 0.01 | 0.00 | 0.01 | 0.01 |
| FukuN18_freshwater_group | 0.00 | 0.00 | 0.00 | 0.00 | 0.00 | 0.00 | 0.00 | 0.00 | 0.00 | 0.00 | 0.00 | 0.00 | 0.00 | 0.00 | 0.00 | 0.00 | 0.00 | 0.00 | 0.00 | 0.00 | 0.00 |
| Terrimicrobium | 0.01 | 0.00 | 0.00 | 0.00 | 0.00 | 0.00 | 0.00 | 0.00 | 0.00 | 0.00 | 0.00 | 0.00 | 0.00 | 0.00 | 0.00 | 0.00 | 0.00 | 0.00 | 0.00 | 0.00 | 0.00 |
| Alterococcus | 0.00 | 0.00 | 0.00 | 0.31 | 0.00 | 0.00 | 0.00 | 0.00 | 0.00 | 0.00 | 0.00 | 0.00 | 0.00 | 0.00 | 0.00 | 0.00 | 0.00 | 0.00 | 0.00 | 0.00 | 0.00 |
| Cephaloticoccus | 0.08 | 0.02 | 0.04 | 0.00 | 0.00 | 0.05 | 0.02 | 0.14 | 0.02 | 0.00 | 0.04 | 0.05 | 0.07 | 0.00 | 0.05 | 0.00 | 0.00 | 0.01 | 0.00 | 0.00 | 0.01 |
| Diplosphaera | 0.00 | 0.00 | 0.00 | 0.00 | 0.00 | 0.09 | 0.00 | 0.02 | 0.00 | 0.00 | 0.01 | 0.00 | 0.00 | 0.00 | 0.00 | 0.00 | 0.00 | 0.00 | 0.00 | 0.00 | 0.00 |
| unclassified_Puniceicoccaceae | 0.00 | 0.00 | 0.00 | 0.00 | 0.00 | 0.00 | 0.00 | 0.00 | 0.00 | 0.00 | 0.00 | 0.00 | 0.00 | 0.00 | 0.00 | 0.00 | 0.01 | 0.01 | 0.01 | 0.00 | 0.00 |
| Cerasicoccus | 0.00 | 0.00 | 0.00 | 0.00 | 0.00 | 0.00 | 0.00 | 0.00 | 0.00 | 0.00 | 0.01 | 0.00 | 0.00 | 0.00 | 0.00 | 0.00 | 0.02 | 0.04 | 0.01 | 0.00 | 0.01 |
| Pelagicoccus | 0.00 | 0.00 | 0.00 | 0.00 | 0.00 | 0.00 | 0.00 | 0.00 | 0.00 | 0.00 | 0.00 | 0.00 | 0.00 | 0.00 | 0.01 | 0.00 | 0.00 | 0.00 | 0.00 | 0.00 | 0.00 |
| Verruc-01 | 0.01 | 0.06 | 0.00 | 0.00 | 0.00 | 0.00 | 0.00 | 0.00 | 0.00 | 0.21 | 0.06 | 0.19 | 0.00 | 0.00 | 0.06 | 0.00 | 0.00 | 0.00 | 0.00 | 0.00 | 0.00 |
| ADurb.Bin063-1 | 0.00 | 0.00 | 0.00 | 0.00 | 0.00 | 0.00 | 0.00 | 0.00 | 0.00 | 0.00 | 0.00 | 0.00 | 0.00 | 0.00 | 0.00 | 0.00 | 0.00 | 0.00 | 0.00 | 0.00 | 0.00 |
| Akkermansia | 0.06 | 0.01 | 0.00 | 0.00 | 0.00 | 0.15 | 0.00 | 0.00 | 0.00 | 0.04 | 0.00 | 0.00 | 0.00 | 0.00 | 0.00 | 0.00 | 0.00 | 0.00 | 0.00 | 0.00 | 0.00 |
| unclassified_DEV007 | 0.01 | 0.00 | 0.00 | 0.01 | 0.00 | 0.01 | 0.00 | 0.01 | 0.05 | 0.00 | 0.12 | 0.14 | 0.01 | 0.08 | 0.06 | 0.00 | 0.01 | 0.04 | 0.03 | 0.01 | 0.01 |
| DBS1 | 0.03 | 0.01 | 0.03 | 0.02 | 0.00 | 0.00 | 0.02 | 0.06 | 0.61 | 0.00 | 0.06 | 0.12 | 0.01 | 0.03 | 0.03 | 0.01 | 0.02 | 0.02 | 0.04 | 0.02 | 0.01 |
| Luteolibacter | 0.00 | 0.01 | 0.00 | 0.00 | 0.00 | 0.00 | 0.01 | 0.00 | 0.00 | 0.00 | 0.00 | 0.00 | 0.00 | 0.00 | 0.00 | 0.00 | 0.00 | 0.00 | 0.00 | 0.00 | 0.00 |
| Persicirhabdus | 0.15 | 0.20 | 0.05 | 0.02 | 0.06 | 0.03 | 0.09 | 0.47 | 1.78 | 0.06 | 1.09 | 0.43 | 0.13 | 0.02 | 0.07 | 0.02 | 0.04 | 0.15 | 0.07 | 0.04 | 0.02 |
| Roseibacillus | 0.00 | 0.00 | 0.00 | 0.00 | 0.00 | 0.03 | 0.07 | 0.01 | 0.15 | 0.00 | 0.04 | 0.02 | 0.02 | 0.00 | 0.00 | 0.00 | 0.00 | 0.03 | 0.00 | 0.00 | 0.00 |
| Rubritalea | 0.02 | 0.00 | 0.01 | 0.01 | 0.00 | 0.03 | 0.02 | 0.80 | 1.68 | 0.09 | 0.59 | 1.42 | 0.13 | 0.05 | 0.08 | 0.01 | 0.04 | 0.09 | 0.10 | 0.06 | 0.03 |
| unclassified_WPS-2 | 0.05 | 0.01 | 0.11 | 0.00 | 0.00 | 0.17 | 0.07 | 0.05 | 0.11 | 0.00 | 0.13 | 0.07 | 0.06 | 0.00 | 0.00 | 0.00 | 0.00 | 0.00 | 0.02 | 0.00 | 0.00 |
| unclassified_Zixibacteria | 0.00 | 0.00 | 0.00 | 0.00 | 0.00 | 0.00 | 0.01 | 0.00 | 0.00 | 0.00 | 0.00 | 0.00 | 0.00 | 0.00 | 0.00 | 0.00 | 0.00 | 0.00 | 0.00 | 0.00 | 0.00 |

**Table S3. All bacterial taxa (log-transformed LDA>2) identified by LEfSe analysis of *M. coruscus* datasets.**

| **Biomarker** | **Class** | | | **log_10_(LDA score)** | ***P*-value** |
| --- | --- | --- | --- | --- | --- |
| Bacteria.Bacteroidetes.Bacteroidia.Flavobacteriales.Flavobacteriaceae.Tenacibaculum | CTR | Live | 21℃ | 4.213 | 1.18E-02 |
| Bacteria.Proteobacteria.Alphaproteobacteria.Rhizobiales |  |  | 21℃ | 4.163 | 1.58E-02 |
| Bacteria.Proteobacteria.Alphaproteobacteria.Rhizobiales.Rhizobiaceae |  |  | 21℃ | 4.090 | 3.48E-02 |
| Bacteria.Firmicutes.Bacilli.Bacillales.Bacillaceae.Bacillus |  |  | 27℃ | 4.409 | 7.20E-03 |
| Bacteria.Proteobacteria.Gammaproteobacteria.Pseudomonadales.Moraxellaceae.Acinetobacter |  |  | 27℃ | 4.272 | 1.22E-02 |
| Bacteria.Firmicutes.Bacilli |  |  | 27℃ | 4.552 | 1.13E-02 |
| Bacteria.Proteobacteria.Gammaproteobacteria.Pseudomonadales.Pseudomonadaceae |  |  | 27℃ | 5.261 | 1.97E-02 |
| Bacteria.Proteobacteria.Gammaproteobacteria.Pseudomonadales.Pseudomonadaceae.Pseudomonas |  |  | 27℃ | 5.268 | 1.97E-02 |
| Bacteria.Proteobacteria.Gammaproteobacteria.Pseudomonadales |  |  | 27℃ | 5.307 | 1.84E-02 |
| Bacteria.Proteobacteria.Gammaproteobacteria.Betaproteobacteriales |  |  | 27℃ | 4.170 | 9.52E-03 |
| Bacteria.Proteobacteria.Gammaproteobacteria.Pseudomonadales.Pseudomonadaceae.Pseudomonas.Pseudomonas_plecoglossicida |  |  | 27℃ | 5.262 | 1.96E-02 |
| Bacteria.Firmicutes.Bacilli.Bacillales.Bacillaceae |  |  | 27℃ | 4.438 | 1.03E-02 |
| Bacteria.Proteobacteria.Gammaproteobacteria.Pseudomonadales.Moraxellaceae |  |  | 27℃ | 4.288 | 1.15E-02 |
| Bacteria.Proteobacteria.Gammaproteobacteria.Pseudomonadales.Moraxellaceae.Acinetobacter.Acinetobacter_calcoaceticus |  |  | 27℃ | 4.097 | 1.78E-02 |
| Bacteria.Firmicutes.Bacilli.Bacillales |  |  | 27℃ | 4.505 | 1.23E-02 |
| Bacteria.Firmicutes.Bacilli.Bacillales.Bacillaceae.Bacillus.Bacillus_velezensis |  |  | 27℃ | 4.370 | 7.84E-03 |
| Bacteria.Actinobacteria |  |  | 27℃ | 4.053 | 1.25E-02 |
| Bacteria.Bacteroidetes.Bacteroidia.Bacteroidales.Bacteroidaceae.Bacteroides | *V. cyclitrophicus*_8 |  | 21℃ | 4.129 | 1.92E-02 |
| Bacteria.Bacteroidetes.Bacteroidia.Bacteroidales.Bacteroidaceae |  |  | 21℃ | 4.134 | 1.92E-02 |
| Bacteria.Bacteroidetes.Bacteroidia.Bacteroidales |  |  | 21℃ | 4.677 | 4.52E-02 |
| Bacteria.Bacteroidetes.Bacteroidia.Flavobacteriales.Flavobacteriaceae.Polaribacter_4 |  |  | 21℃ | 4.431 | 8.19E-03 |
| Bacteria.Proteobacteria.Alphaproteobacteria.Rhodospirillales.Terasakiellaceae |  |  | 27℃ | 4.413 | 6.17E-03 |
| Bacteria.Proteobacteria.Alphaproteobacteria.Rhodospirillales |  |  | 27℃ | 4.434 | 6.84E-03 |
| Bacteria.Proteobacteria.Gammaproteobacteria.Francisellales | *V. cyclitrophicus*_9 |  | 21℃ | 4.456 | 8.64E-03 |
| Bacteria.Epsilonbacteraeota.Campylobacteria.Campylobacterales.Arcobacteraceae.Arcobacter |  |  | 21℃ | 4.932 | 2.64E-02 |
| Bacteria.Proteobacteria.Gammaproteobacteria.Alteromonadales |  |  | 21℃ | 4.804 | 7.70E-03 |
| Bacteria.Proteobacteria.Gammaproteobacteria.Francisellales.Francisellaceae |  |  | 21℃ | 4.467 | 8.64E-03 |
| Bacteria.Epsilonbacteraeota.Campylobacteria.Campylobacterales.Arcobacteraceae |  |  | 21℃ | 4.939 | 2.64E-02 |
| Bacteria.Epsilonbacteraeota |  |  | 21℃ | 4.904 | 2.64E-02 |
| Bacteria.Patescibacteria |  |  | 21℃ | 4.646 | 1.21E-02 |
| Bacteria.Proteobacteria.Gammaproteobacteria.Francisellales.Francisellaceae.Francisella |  |  | 21℃ | 4.422 | 8.64E-03 |
| Bacteria.Proteobacteria.Gammaproteobacteria.Alteromonadales.Pseudoalteromonadaceae.Pseudoalteromonas |  |  | 21℃ | 4.801 | 1.34E-02 |
| Bacteria.Patescibacteria.Parcubacteria.Candidatus_Campbellbacteria |  |  | 21℃ | 4.679 | 5.05E-03 |
| Bacteria.Epsilonbacteraeota.Campylobacteria |  |  | 21℃ | 4.908 | 2.64E-02 |
| Bacteria.Patescibacteria.Parcubacteria |  |  | 21℃ | 4.669 | 7.86E-03 |
| Bacteria.Proteobacteria.Gammaproteobacteria.Alteromonadales.Pseudoalteromonadaceae |  |  | 21℃ | 4.808 | 6.24E-03 |
| Bacteria.Epsilonbacteraeota.Campylobacteria.Campylobacterales |  |  | 21℃ | 4.923 | 2.64E-02 |
| Bacteria.Proteobacteria.Gammaproteobacteria.Vibrionales.Vibrionaceae |  |  | 27℃ | 5.125 | 1.37E-02 |
| Bacteria.Proteobacteria.Gammaproteobacteria.Vibrionales |  |  | 27℃ | 5.099 | 1.37E-02 |
| Bacteria.Firmicutes.Clostridia.Clostridiales.Family_XII |  |  | 27℃ | 4.035 | 2.58E-02 |
| Bacteria.Firmicutes.Clostridia.Clostridiales.Family_XII.Fusibacter |  |  | 27℃ | 4.042 | 4.64E-02 |
| Bacteria.Proteobacteria.Gammaproteobacteria.Vibrionales.Vibrionaceae.Vibrio |  |  | 27℃ | 5.106 | 1.66E-02 |
| Bacteria.Bacteroidetes.Bacteroidia.Flavobacteriales.Flavobacteriaceae._Polaribacter_huanghezhanensis |  |  | 27℃ | 4.466 | 2.02E-02 |
| Bacteria.Bacteroidetes.Bacteroidia.Flavobacteriales.Cryomorphaceae |  |  | 27℃ | 4.683 | 2.35E-02 |
| Bacteria.Proteobacteria.Gammaproteobacteria.Oceanospirillales |  | Dead | 27℃ | 4.713 | 9.79E-03 |
| Bacteria.Proteobacteria.Gammaproteobacteria.Oceanospirillales.Nitrincolaceae.Amphritea |  |  | 27℃ | 4.683 | 6.39E-03 |
| Bacteria.Proteobacteria.Gammaproteobacteria.Oceanospirillales.Nitrincolaceae |  |  | 27℃ | 4.712 | 7.68E-03 |
